# Effects of thiol substitution in deep-eutectic solvents (DESs) as solvents for metal oxides†‡

**DOI:** 10.1039/d0ra03696j

**Published:** 2020-06-19

**Authors:** Giacomo Damilano, Antero Laitinen, Pia Willberg-Keyriläinen, Tiina Lavonen, Riina Häkkinen, Wim Dehaen, Koen Binnemans, Lauri Kuutti

**Affiliations:** KU Leuven, Department of Chemistry Celestijnenlaan 200F – P. O. Box 2404 B-3001 Leuven Belgium; VTT Technical Research Centre of Finland Ltd Tietotie 4E FI-02150 VTT Finland lauri.kuutti@vtt.fi +358 20 722 2936

## Abstract

This study deals with an investigation of how substitution of an alcohol group by a thiol group in mixtures of choline chloride with a series of bio-sourceable molecules affects the physico-chemical properties of the mixtures and their ability to dissolve metal oxides. All of the thiol mixtures studied showed a higher affinity and selectivity for late transition metals and the physical properties of the mixtures were improved compared to their alcohol analogues (*i.e.* lower viscosity, wider liquid range). The metal solubility was assessed *via* determination of the final concentration of the metal oxides dissolved in thiol mixtures *via* inductively coupled plasma optical emission spectroscopy (ICP-OES). The thiol function selectively improved the solubilities of the late transition metal oxides (*i.e.* copper and zinc), which are valuable metals often present as residue in industrial waste. The solubility of iron oxides was much lower than that of the valuable metals, which is a significant benefit in industrial applications. The different solubilization behaviour of metal oxides in the thiol and alcohol mixtures was further investigated *via* UV-vis absorption and infrared spectroscopy. This study allowed the potential of these deep-eutectic solvents for the selective recovery of metals to be assessed.

## Abstract 2

The study compared thiol LTTM to their alcoholic analogues. This study set the basis for the discovery of this alternate domain of thiol LTTMs.

## Graphical abstract

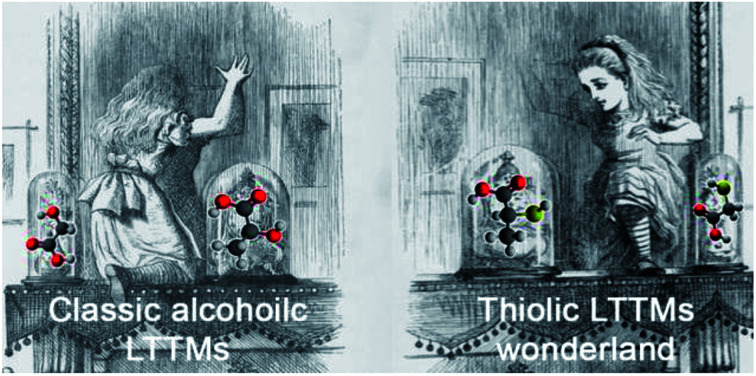

## Introduction

Efficient extraction and recovery of metals from ores and industrial process residues is fundamental to meet the societal demands for raw materials. Metals are central to all current and foremost advanced technologies; therefore, they are also a substantial fraction of the electric and electronic waste. This entails metal recovery and processing to assume a crucial role in supplying the basic amount of raw materials, either *via* processing of ores or *via* the recycling of industrial and electronic waste.

Classic hydrometallurgical processes often use a large volume of water to achieve separation, which is an effective but not efficient procedure. Water is a harmless green solvent but it must be purified prior to its disposal. Although viable, the purification process is impractical due to the large volumes of waste water produced in hydrometallurgical processes. In order to shift to a more sustainable process, non-aqueous systems are being studied as alternative media for solvometallurgical processes.

Solvometallurgy is the branch of extractive metallurgy that attempts an almost complete substitution of water with an organic solvent.^1^ Besides reducing the consumption of water, an accurate choice of the organic solvent greatly improves the process selectivity and reactivity, allowing for the development of more efficient and sustainable processes. Amongst many, ionic liquids (ILs) and deep-eutectic solvents (DESs) are appealing as solvents for solvometallurgical applications.^2,3^ The interest in this category of solvents arose from both their affirmation as an environmentally benign technology, due to the low volatility and high stability, and from their fine tunability to the needs of the process *via* an appropriate choice of the solvent mixtures.

As the solvent is the most abundant component of a system, its low cost is often crucial to the attainment of a financially viable process. The large scale viability, reduced costs, and ease of production are reasons to often prefer deep-eutectic solvents over ionic liquids. Abbott *et al.* firstly introduced the definition of a deep-eutectic solvent as a eutectic mixture of two or more components with a depression in the freezing-point at about or below 100 °C (arbitrary temperature) and a composition close to the one of the eutectic point.^2,4,5^

The growing amount of research concerning these mixtures is urging for a more accurate definition for these materials. A number of bio-sourceable materials was investigated in the literature as mixture components. For many of these mixtures, a depression of the solidification transition temperature was recorded, but not a freezing-point. In the absence of a freezing point, we can no longer discuss about eutectic mixtures nor about deep-eutectic solvents, because the definition of the latter is dependent on the former. Therefore, low-transition-temperature mixtures (LTTMs) appears to be a more suitable term to describe these mixtures.^6^

Abbott *et al.* studied a wide array of choline chloride mixtures with various components such as oxalic acid, ethylene glycol, malonic acid, and urea.^2–9^ Both choline chloride DESs and DESs resulted in an effective metal dissolution, showing often remarkably high metal solubilities for a non-aqueous system.^3,7,10–13^ In these medias, the high metal solubility (*i.e.* metal salts, oxides and hydroxides) is also contributing to their high efficacy in applications such as metal extraction and recovery. For example, often a selective metal recovery can be attained *via* the watering down of the system, which alters the metal solubilities. Moreover, the relatively wide electrochemical window, the chemical stability, and thermal stability allow for the use of electro-winning, a simple metal recovery technique, from these mixtures.

Although, the viscosity of the pure non-aqueous solvent systems is sometimes low, the dissolution of metals in these mixtures often elicits a steep increase of their viscosity. Avoiding this is therefore of utmost importance for process scale-up economies because the adhesion of such viscous media on the undissolved materials and reactor walls causes substantial losses, that could be up to 40 wt%. Previous studies on the use of ILs and DESs in tribological applications showed a correlation between the decrease in viscosity and the inclusion of organo-sulfur groups in ionic liquids and deep-eutectic solvents.^14^ As the hydrogen bond interactions are the likely cause of the atypical features of low temperature-transition mixtures, the reduction in viscosity could be rationalised by a loosening of the hydrogen bond network due to a weakening of the electrostatic interactions formed with the organo-sulfur atoms.^4,6^

In general, most of the studied mixtures includes amino, ammonium, carboxylic acid, or alcohol groups in the molecular structure but rarely organo-sulfur groups (*e.g.* thio-urea DESs).^15,16^ However, in complexation chemistry many sulfur compounds have demonstrated a high selectivity and affinity for precious and late transition metals (*i.e.* group 10 and 12).^17–22^ This can be explained by the concept of hard and soft acid and bases (HSAB concept).^23^ Often these metals are also the target of industrial waste recycle processes because they contain most of the residual metal value. For example, both thio-glycolic and thio-lactic acid have been exploited in various fields (*i.e.* cosmetics, metal titration, metal complexation, waste-water treatment).^19,20,24–29^ They are synthesised easily from their bio-sourceable oxy-analogues and have a significant affinity for late transition metals (*i.e.* copper and zinc).^24^

In this paper, we describe how the introduction of thiol groups in bio-sourceable alcohols and acids can influence the physico-chemical properties of the mixture and the solubilities of metal oxides in such solvents.

## Experimental

### Chemicals and solvents

Choline chloride (ChCl) (≥98%, 67-48-1), dl-malic acid (MA) (≥98%, 6915-15-7), dl-thiomalic acid (TMA) (>98.0%, 70-49-5), dl-lactic acid (LA) (90% solution in water, 50-21-5), dl-thiolactic acid (TLA) (>97.0%, 79-42-5), glycolic acid (GA) (≥99%, 79-14-1), thioglycolic acid (TGA) (≥98%, 68-11-1), cadmium(ii) oxide (99.5%, 1306-19-0), cobalt(ii) oxide (99.99%, 1307-96-6ý), nickel(ii) oxide (99.8%, 1313-99-1), lead(ii) oxide (99.999%, 1317-36-8), iron(ii) oxide (99.7%, 1345-25-1), iron(ii, iii) mixed oxide (98%, 1309-37-1), zinc(ii) oxide (99.99%, 1314-13-2) were purchased from Sigma-Aldrich (Espoo, Finland). dl-Dithiothreitol (DTT) (>99%, 3483-12-3) was purchased from Apollo Scientific (Manchester, United Kingdom). Lithium cobalt(iii) oxide (≥98%, 12 190-79-3ý) and gold(iii) oxide (99%, 1303-58-8) were purchased from STREM (Helsinki, Finland). Copper(ii) oxide (≥97.5%, 1317-39-1ý) was purchased from VWR (Helsinki, Finland). Copper(i) oxide (99.7%, 1317-39-1ý), and silver(i) oxide (>99.0%, 20667-12-3) were purchased from Fluka AG (Bucks, Switzerland). Choline chloride and lactic acid were dried prior to use. Choline chloride was dried at high vacuum for at least 24 h prior to use. All the other chemicals were used without further purification.

### Instrumentation and materials

Differential scanning calorimetry (DSC) analyses was carried out on a DSC apparatus (Mettler-Toledo DSC820 STARe System, Switzerland) in the temperature range from −120 to 25 °C and a heating rate of 10 °C min^−1^. Rheological analyses were carried out with a cone and plate rheometer (Anton-Paar Physica MCR 301, Germany) equipped with a conic spindle (CP50-2/TG). The instrument was equipped with a thermal control unit to allow the investigation at temperatures between 25 and 60 °C. The viscosity was measured in triplicates as a function of the shear rate. NMR measurements were performed on a NMR spectrometer (Bruker Avance III HD) operating at a ^1^H frequency of 500 MHz at 22 °C. Fourier transform infrared (FTIR) measurements were carried out using a FTIR spectrometer (Thermo Fisher Nicolet iS50, USA) and recorded on a diamond-ATR (attenuated total reflectance) module with a resolution of 2 cm^−1^. UV-vis spectra were carried out on a UV-vis double beam spectrophotometer (Shimadzu UV-2600, Japan) with quartz cuvettes (10 × 10 mm). Solution samples were assayed for Ag, Au, Co, Cd, Cu, Fe, Ni, Pb, and Zn content on an inductively coupled plasma optical emission spectroscopy (ICP-OES) apparatus (5100 SVDV, Agilent Technologies). ICP-OES measurements were performed using multi-element standards (Inorganic Ventures and SPEX) for calibration lines and control samples. Centrifugation was carried out on a refrigerated centrifuge (Eppendorf 5804 R) at 4000 and 40 °C rpm for 45 min. For the estimation of the pH, pH-indicator strips (ColorpHast, Merck) were used with a sensitivity of 1 unit over a pH range from 0 to 14. Disposable syringe filters (Whatman, PVDF membrane in PP housing, 25 mm Ø, 0.45 μm pore Ø) were used for sample filtration.

### Methodology

The DESs were synthesised *via* magnetic stirring until an homogeneous mixture was formed (vide Table 1 for further details). The prepared DESs were characterized prior to use and preserved at 6 °C. Dissolution was realised *via* stirring the DES (2 mL) with a small amount of metal oxide (100 mg). If the metal was fully dissolved, additional metal oxide was added (100 mg) until no more dissolution was observed. The addition of precious metals to the thiol-acids was done under a high flow of inert gas to impede the ignition of the gas released by the reaction of the thiol DES with the precious metal oxide. After dissolution, the sample was centrifuged and filtered to remove undissolved residues. The filtrate was collected and tested *via* ICP-OES. Solution samples were assayed for Au, Cd, Co, Ag, Cu, Fe, Li, Ni, Pb, and Zn content. Samples were diluted 1 : 100 and 1 : 1000 with 1% HNO_3_ solution (optima grade). If the samples formed a precipitate after dilution, the samples were centrifuged and the supernatant was analysed. When precipitation was observed, the sample was newly tested after being pre-processed *via* a microwave digester. About 50 mg of the solution samples were weighted in a PTFE-lined microwave digestion tubes and digested with reverse *aqua regia* (ultra-pure grade). The digested samples were diluted to 20 mL with milliQ water and measured without further dilution.

**Table 1:** Visual observation of the minimum conditions required to the formation of the DES under magnetic stirring. All molar ratio from of 3 : 1 to 1 : 3 were tested but herein are reported only the mixtures achieving an homogeneous liquid phase

HBA : HBD (molar ratio)	Appearance at 20–22 °C	*t* (h)	*T* (°C)
ChCl : GA (1 : 2)	Clear homogeneous liquid	2	20–22
ChCl : GA (1 : 3)^a^	Clear homogenous liquid	2	20–22
ChCl : TGA (1 : 2)	Clear homogenous liquid	2	20–22
ChCl : TGA (1 : 3)	Clear homogenous liquid	2	20–22
ChCl : LA (1 : 2)	Clear homogenous liquid	2	20–22
ChCl : LA (1 : 3)	Clear homogenous liquid	2	20–22
ChCl : TLA (1 : 2)	Clear homogenous liquid	2	20–22
ChCl : TLA (1 : 3)	Clear homogenous liquid	2	20–22
ChCl : MA (1 : 1)	Clear homogenous liquid	2	60–70
ChCl : TMA (1 : 1)	Clear faint yellow homogenous liquid	4	60–70
ChCl : DTT (1 : 2)	Clear homogenous liquid	3	20–22
ChCl : DTT (1 : 3)	Clear homogenous liquid	3	20–22

a A precipitate was observed after refrigerating the sample overnight at 6 °C.

## Results and discussion

**Fig. 1:**
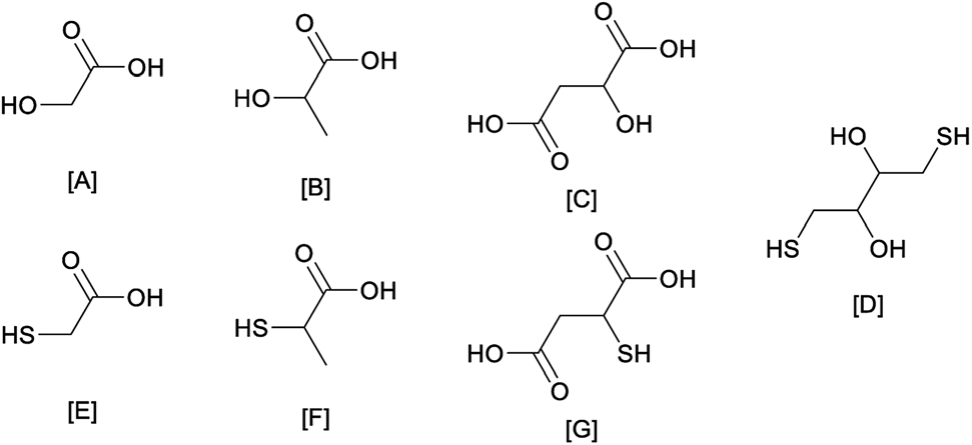
Graphical representation of the HBD used in our analysis. From the top left corner to the bottom right corner: [A] – glycolic acid (GA), [B] – lactic acid (LA), [C] – malic acid(MA), [D] – dithiothreitol (DTT), [E] – thioglycolic acid (TGA), [F] – thiolactic acid(TLA) and [G] – thiomalic acid (TMA).

A series of choline chloride DESs was characterised. The focus was on how inter-molecular interactions are affected by the substitution of an alcohol group with a thiol analogue in a series of structures (Fig. 1). The substitution of the oxygen atom by a sulfur atom was also valued against its effect on acidity and metal complexation, which should favour a higher selectivity towards late transition metals (with softer metal ions). In our analysis, we focused on sulfur analogues of widely available bio-derivable feedstocks. These were selected due to their large scale use in fine chemicals, which was indicative of reduced toxicity, low cost, and large scale availability (*e.g.* thioglycolic and thiolactic acids are used in hair removal creams and perm treatments).^27,28^ Therefore, thioglycolic acid, thiolactic acid, thiomalic acid, and dithiothreitol were analysed against some of their alcohol analogues (Fig. 1).

The formation of the DESs was studied to determine the appropriate components ratio and the softest condition for the homogenization of these mixtures. Most of the DESs were obtained *via* simple mixing at room temperature (22 °C), but few required higher temperatures (Table 1). The HBA : HBD ratio achieving a clear homogeneous eutectic mixture followed the empirical ratio formerly observed by Abbott *et al.* for other mono and di-acids.^5^ The formation of the DESs was eased by the mutual dispersion of the components into each other. Although time-demanding, mixing the components of the DES at room temperature allowed to have virtually no ester formation. This was verified *via* NMR analysis, which confirmed the virtual absence of esterification. If the homogenization was expedited, for example *via* the use of higher temperatures, then the ^1^H NMR signals of esterification side-products became noticeable.^30^ In order to minimize ester formation, the less energy intensive procedure was used.^30^ To avoid further esterification and achieve more reliable results, the prepared DES were stored at 6 °C. The acidity of the mixtures was estimated *via* pH strip indicators, showing an average pH of 3 for the mixtures containing an acid function (*i.e.* ChCl : GA(1 : 2), ChCl : TGA(1 : 2), ChCl : LA(1 : 2), ChCl : TLA(1 : 2), ChCl : MA(1 : 2), ChCl : TMA(1 : 2)) and a pH of 5 for the ChCl : DTT(1 : 3) mixture (Table 2). Generally, a lower acidity is experienced in this type of media because the charged protic environment inhibits the full ionization of the acidic species.^8^ Instead, the DESs had a slightly higher acidity with respect to the pure substances (*e.g.* the used acids have in average a p*K*_a_ ranging from about 3.1 to 3.8). This effect may be due to the coordination of chloride anions to hydrogen through H-bond interactions. This may result in an overall weaker X–H bond (X = O, S) and a higher concentration of ionized acidic groups. The synthesised mixtures were characterised *via* differential scanning calorimetry. All of the mixtures showed a glass phase transition (Table 2).^30^

**Table 2:** Physical characterization of the DESs

HBA : HBD (ratio)	*η* a (Pa s)	g. t. p.^b^ (°C)	p*K*_a_
ChCl : GA (1 : 2)	0.3 ± 0.0	−79 ± 0	3
ChCl : GA (1 : 3)	—	−78 ± 2	3
ChCl : TGA (1 : 2)	—	−95 ± 0	3
ChCl : TGA (1 : 3)	—	−94 ± 2	3
ChCl : LA (1 : 2)	0.7 ± 0.0	−70 ± 2	3
ChCl : LA (1 : 3)	—	−68 ± 2	3
ChCl : TLA (1 : 2)	0.1 ± 0.0	−88 ± 0	3
ChCl : TLA (1 : 3)	—	−87 ± 3	3
ChCl : MA (1 : 1)	30.4 ± 0.6	−48 ± 0	3
ChCl : TMA (1 : 1)	28.1 ± 0.8	−58 ± 1	3
ChCl : DTT (1 : 2)	—	−64 ± 3	5
ChCl : DTT (1 : 3)	0.32 ± 0.0	−69 ± 3	5

a Viscosity measured at 25 °C (*vide* ESI).

b Glass transition temperature according to the DSC analyses.

In order to limit the number of analyses, a subset of the synthesised DESs was used in the following tests (*i.e.* ChCl : GA (1 : 2), ChCl : TGA (1 : 2), ChCl : LA (1 : 2), ChCl : TLA (1 : 2), ChCl : MA (1 : 1), ChCl : TMA (1 : 1), ChCl : DTT (1 : 3)). Viscosimetry was carried out but the ChCl : TGA (1 : 2) mixture was excluded from the analyses due to safety concerns. Thioglycolic and thioglycolate toxicity is reported to be concentration dependent, which is also the reason why it is widely used in consumer products.^31^ To avoid any risk, we excluded the analyses of mixtures of thioglycolic acid out of a fumehood. Viscosimetry showed a marked reduction in viscosity for all the thiol derivatives with respect to the alcohol ones (Table 2). The significant reduction in viscosity is beneficial for the intended application use of these mixtures.^14^ Both the ChCl : MA (1 : 1) and the ChCl : TMA (1 : 1) had a remarkably higher viscosity, even at elevated temperatures (30.4 and 28.1 Pa s respectively at 25 °C). In this case, the higher viscosity impeded an effective homogenization of the sample *via* magnetic stirring. Therefore, these mixtures were excluded from the metal oxides dissolution study.

Metal oxide dissolution of various sample metals was tested to define a general solubility trend in the studied DESs. The solubility of a series of metal oxides belonging to the late transition metal group was studied: Ag_2_O, Au_2_O_3_, CdO, CoO, LiCoO_2_, CuO, Cu_2_O, FeO, Fe_2_O_3_, Fe_3_O_4_, NiO, PbO, and ZnO. These metals were selected in order to observe the effect of the heteroatom substitution on the chelating capabilities of the various mixtures. Each metal oxide was added to a separate DES sample until saturation was reached. After 48 h, the samples were centrifuged and filtered and the filtrate was tested to evaluate the dissolution process.

A preliminary test of the dissolution effects was carried out *via* FTIR spectroscopy. FTIR analysis was directed to analyse the interaction of the functional groups belonging to the organic complexing agent with the dissolved metals (Table 3). Only the ChCl : DTT (1 : 3) mixture had no remarkable variations, with respect to the pure sample, in the reflectance signals. Because for the ChCl : DTT (1 : 3) mixture no characteristic variation could be observed *via* FTIR analysis, the following discussion on the FTIR analyses will focus on the other mixtures tested.

**Table 3:** Characteristic IR absorption bands (cm^−1^) for the studied DESs^26^^,^^a^

ChCl : GA (1 : 2)	ChCl : TGA (1 : 2)	ChCl : LA (1 : 2)	ChCl : TLA (1 : 2)	ChCl : DTT (1 : 3)	Assignment
3280 (w)	3280 (w)	3279 (w)	—	3309 (vs.)	v (OH)_COOH_
	2564 (w)		2553 (w)	2548 (w)	v (SH)
1729 (vs.)	1717 (vs.)	1730 (vs.)	1723 (vs.)		v (C=O)_COOH_
1419 (m)	1414 (m)	1456 (m)	1452 (m)		v_sy_ (–CO_2_)_COOH_
		1194 (vs.)	1171 (vs.)		ω (CH_2_)
1079 (vs.)	1082 (m)	1084 (m)	1077 (m)	1066 (vs.)	v (C–OH)_COOH_
	1049 (m)	1043 (m)	1057 (m)	1044 (vs.)	v (C–OH)_CH_2_OH_
993 (w)	1003 (w)	1005 (m)	1002 (w)	1005 (w)	v (C–C)

a vs. very strong, s strong, m medium, w weak, sh shoulder. ν stretching, ν_sy_ symmetric, ν_as_ asymmetric, δ_s_ bending, ρ rocking, ω wagging, τ twisting.

For the DES containing an acid group, metal oxide dissolution happens generally *via* the neutralization of the oxide and the formation of a metal complex. Therefore, the metal oxide neutralization was followed by the appearance of a peak at about 1550–1590 cm^−1^, which was caused by the asymmetric stretch of the carboxylate group. In all of the samples where dissolution of the metal oxide occurred, there was also the appearance of the carboxylate group signal. In correspondence to the appearance of the carboxylate group signal also some reduction in the intensity of the signals of the carboxylic acid group at about 1715–1730 cm^−1^ and of the hydroxyl group stretch at 1040–1090 cm^−1^ was observed.

The appearance of the carboxylate signal was generally consistent with the results obtained with the ICP-OES on metal dissolution. However, for some samples ICP-OES analyses did show only a negligible amount of dissolved metals whilst the FTIR analyses showed a marked formation of the carboxylate anion. This was observed especially for lead and to some extent also for nickel and cobalt samples. The absence of dissolved metals was suggestive of the formation of a precipitate, which was filtered off after dissolution and prior to the ICP-OES analysis. This was reasoned out by the possible formation of insoluble hydroxides and/or salts by reaction with the DES. The acidic group of the DES might have reacted with the oxides, resulting in the formation of metal salts (either chloride or carboxylate) and eventually also hydroxides. Then, these might have precipitated out because they are poorly soluble in the DES (*vide* ESI, Visual report). Noticeable was that nickel(ii) oxide was generally insoluble in the assayed mixtures and strongly reacted with the ChCl : TLA (1 : 2) (determined *via* FTIR analysis). This resulted in the formation of a thick solid paste, which could not be filtered, impeding any further analyses.

The determination of the dissolved metal content into the mixture was carried out *via* ICP-MS analysis (Table 4). The ChCl : GA (1 : 2) mixture was able to dissolve various metals: CdO (96 g L^−1^), Cu_2_O (84 g L^−1^), ZnO (50 g L^−1^), Au_2_O_3_ (42 g L^−1^), Fe_2_O_3_ (27 g L^−1^). Substitution of glycolic with thioglycolic acid strongly affected metal solubilities. The ChCl : TGA (1 : 2) mixture was able to dissolve more than the double of the amount of cadmium and zinc dissolved in the ChCl : GA (1 : 2) and no cupric nor cuprous oxides. In the ChCl : TGA (1 : 2) mixture, the concentration of dissolved metals was as follows: CdO (213 g L^−1^), ZnO (122 g L^−1^), FeO (29 g L^−1^), Fe_2_O_3_ (23 g L^−1^), CoO (43 g L^−1^), LiCoO_2_ (22 g L^−1^). This shows a marked increase in the dissolution selectivity of the metals belonging to the Group 12 of the periodic system (*i.e.* Cd and Zn). Moreover, this mixture outperformed all the others in the dissolution of cobalt, both as cobalt oxide and as lithium cobalt oxide.

**Table 4:** Dissolved metal content in the various DES (g L^−1^)

	FeO	Fe_2_O_3_	CoO	LiCoO_2_	NiO	Cu_2_O	CuO	Ag_2_O	Au_2_O_3_	ZnO	CdO	PbO
ChCl : GA (1 : 2)	2.99	27.9	6.97	3.5	0.1	84.18	3.11	0.62	42.22	50.28	96.96	0.82
ChCl : TGA (1 : 2)	29.91	23.79	43.98	22.11	2.44	0.62	0.63	0.67	0.03	122.07	213.31	0.51
ChCl : LA (1 : 2)	3.86	29.61	8.3	11.62	0.27	77.16	25.36	0.58	31.22	13.52	42.09	0.22
ChCl : TLA (1 : 2)	20.21	50.42	0.35	0	0	83.99	60.22	0.63	0.05	90.95	93.75	0.26
ChCl : DTT (1 : 3)	9.89	30.68	1.46	0.93	4.51	2.93	3.18	0.27	3.19	21.85	12.67	80.81
ChCl : ethylene glycol (1 : 2)^a^	0.00	0.00	0.01	0.00	0.00	0.36	0.00	—	—	0.43	—	—
ChCl : malonic acid (1 : 2)^a^	4.28	0.32	3.10	0.00	0.13	15.67	11.97	—	—	13.86	—	—

a The data herein reproduced may have been obtained *via* a different procedure. The data were converted from ppm assuming the density of ChCl : EG (1 : 2) and ChCl : malonic acid (1 : 2) at 50 °C to be 1.10 g mL^−1^ and 1.17 g mL^−1^, respectively.^11^

A similar trend was observed for the mixtures of ChCl : LA (1 : 2) and ChCl : TLA (1 : 2). The ChCl : LA (1 : 2) mixture was able to dissolve the following metals: Cu_2_O (80 g L^−1^), CdO (46 g L^−1^), Au_2_O_3_ (31 g L^−1^), CuO (23 g L^−1^), Fe_2_O_3_ (23 g L^−1^). Differently from what was previously observed for the ChCl : GA (1 : 2) mixture, in the ChCl : LA (1 : 2) mixture the concentration of zinc oxide fell well below the concentration of cadmium (about ⅓). Conversely, in the ChCl : TLA (1 : 2) mixture both cadmium and zinc were well dissolved but also a higher concentration of iron was dissolved with respect to the ChCl : LA (1 : 2). Overall, in the ChCl : TLA (1 : 2) the dissolution trend was as follows: CdO (93 g L^−1^), PbO (90 g L^−1^), Cu_2_O (83 g L^−1^), CuO (60 g L^−1^), Fe_2_O_3_ (50 g L^−1^), FeO (20 g L^−1^). Noticeably, ChCl : TLA (1 : 2) was the best mixture for the dissolution of copper oxides, both as cupric and cuprous oxide.

The ChCl : DTT (1 : 3) had strongly different dissolution trends, corresponding with its dissimilar and less acidic dithiothreose sugar structure. In this mixture, metal oxides were dissolved to a lower extent: PbO (80 g L^−1^), Fe_2_O_3_ (30 g L^−1^), ZnO (21 g L^−1^), CdO (12 g L^−1^). Lead was the only metal with a marked solubility in this mixture, and the ChCl : DTT (1 : 3) was the sole mixture capable to solubilize PbO. In this case, the low dissolution could be correlated not only to the ligand structure but also to the overall reduced media acidity, which might have influenced oxide dissolution.

A similar result was observed for the dissolution of cobalt(ii) oxide in the ChCl : TGA (1 : 2). As the graph shows, the highest dissolution of cobalt oxide is present in the ChCl : TGA (1 : 2) mixture, both as cobalt(ii) oxide and as lithium cobalt(iii) oxide. However, a few days after dissolution the filtrate of the cobalt samples presented some spontaneous precipitation. This phenomenon was further studied *via* the UV-vis absorption spectroscopy. The UV-vis spectra showed two groups of peaks: one group assigned to the cobalt(ii) chloride complex (631 nm, 668 nm, 696 nm), the other group to the thioglycolate complex (324 nm, 405 nm, 507 nm).^32,33^ The sample was of a turbid dark purple/brown colour and it was filtered to remove the turbid precipitate observed. After filtration the sample had a clear light blue colour. The UV-vis spectra of the filtered sample confirmed the disappearance of the group of peaks belonging to the thioglycolate complex. When the spectra of the sample were measured as a function of time, the slow re-appearance of the thioglycolate group of peaks was observed. This indicates that the dissolved cobalt(ii) was soluble as a cobalt chloride and slowly precipitated out of the solution as a thioglycolate complex.

The dissolution of coinage metal oxides was analysed. Whereas silver was insoluble in all of the samples, gold and copper oxides were soluble in a few mixtures. Gold oxide was soluble only in the mixtures not containing thiols (*i.e.* ChCl : GA (1 : 2) and ChCl : LA (1 : 2)). On the other hand, copper was soluble as both the copper oxides (*i.e.* CuO and Cu_2_O) in both the ChCl : LA (1 : 2) and the ChCl : TLA (1 : 2) mixtures, while only copper(i) oxide was soluble in ChCl : GA (1 : 2) (Table 4).

The dissolution of the late transition metals belonging to Group 12 was then investigated. For these metals, the UV-vis spectra were not of use because these divalent metal ions are spectroscopically silent. In all the samples there was dissolution of both zinc and cadmium. The highest level of dissolution was experienced in ChCl : TGA (1 : 2), but also ChCl : TLA (1 : 2) performed well (Table 4).

**Fig. 2:**
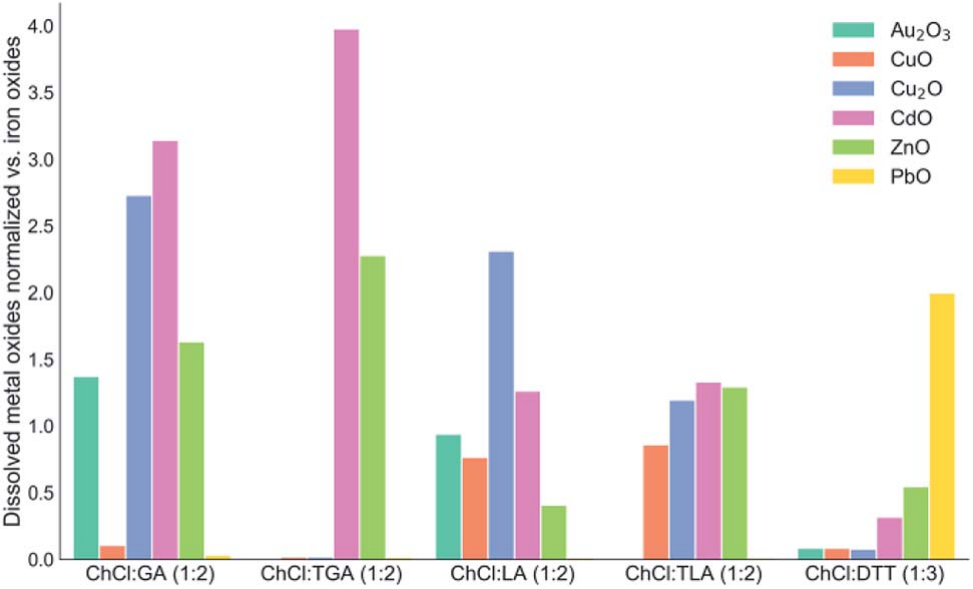
Graphical representation of the dissolution selectivity of the subset of the metal oxides selectively dissolved in large amounts in the DESs *vs.* iron oxides dissolution. This is a numerical elaboration of the data are listed within Table 4. Each of the listed metal oxide was normalized by the amount of iron oxides dissolved under the same conditions.

Iron had a marginal but still significant dissolution. Iron was mostly soluble as iron(iii) but the mixtures of the thiol-acids were also capable to dissolve iron(ii) oxide. The ChCl : TLA (1 : 2) mixture had the highest solubilizing power for iron. The selectivity analysis of the metal dissolution within the various mixtures clearly shows how the iron solubility affects metal selectivity against it (Fig. 2). Considering both the metal oxide solubility and the dissolution selectivity it can be seen that cadmium and zinc had the highest solubility in the ChCl : TGA (1 : 2) mixture. In this medium, we report the highest metal content and the highest selectivity for the metals belonging to the Group 12. Comparatively, copper and gold showed overall a higher dissolution and selectivity in the α-alcohol–acid mixtures with respect to the α-thiol–acid counterparts. Finally, the ChCl : DTT (1 : 3) mixture showed a remarkable selectivity for lead and it was the only mixture of the studied DESs to dissolve PbO to a significant extent.

Another sample analysis was done *via* dilution of the DES samples (1 : 100 or 1 : 1000) with an aqueous solution of nitric acid (1%) (*vide* ESI Table S1†). After this dilution, some samples formed a precipitate, which was filtered off. The ICP-OES analysis indicated a virtually complete precipitation of gold and a partial precipitation of few other metals (*vide* ESI Table S1;†*i.e.* Cu, Zn, Co). The complete precipitation of gold was mostly selective with respect to the other metals studied and it has potential as a gold concentration technique from mixed metals matrices.^34^ This behaviour can be explained by the need for a strongly complexing anion to solubilize the Group 10 metals in aqueous mixtures. The absence of a strongly complexing anion, such as the chloride anion in the *aqua regia* sample, resulted therefore in the precipitation of the dissolved metal in the solution where only water is present. Analogously to gold, copper precipitated but to a minor extent in most of the samples. The ChCl : TLA (1 : 2) mixture was an exception to such behaviour, having the highest levels of dissolution and precipitation of copper, reaching an 81% of overall copper content precipitation. Instead, zinc had a minor but consistent precipitation throughout the samples (15–25%).

## Conclusions

A wide variety of choline chloride mixtures has been investigated for processing of metal compounds. In this study, we investigated the influence of thiol substitution to alcohol groups in choline chloride deep-eutectic solvents.

All thiol-containing DESs showed improved physical properties (*i.e.* lower viscosity, wider liquid range). Moreover, thiol-containing DESs had improved solubilities for late transition metals (*i.e.* copper and zinc), which are valuable metals often present in low grade ores or metal processing residues.

Group 12 metal oxides achieved the best dissolution in β-thiolacids mixtures. These metals were dissolved in high amounts and a fair selectivity. Aqueous dilution (with nitric acid 1%) of such mixtures yielded a partial selective precipitation of zinc. A different trend was observed for Group 10 metal oxides. While gold was not dissolved in the thiol-containing DESs, the best dissolution for copper oxides was achieved in the ChCl : TLA. Over 80% of the dissolved copper could be recovered *via* precipitation in water (with nitric acid 1%).

In conclusion, thiol DESs have better physico-chemical properties then their alcohol analogues and metal affinities which may allow their use as agents in the selective dissolution of valuable late transition metals (*i.e.* copper and zinc) and toxic metals (*e.g.* cadmium and lead), which are incompatible with aqueous processing.^35^ In order to determine the applicative efficacy, further studies may be directed to test the dissolution of actual samples in the studied DESs.

## Conflicts of interest

There are no conflicts to declare.

## Supplementary Material

RA-010-D0RA03696J-s001
