# Supplementary material for: Effects of thiol substitution in deep-eutectic solvents (DESs) as solvents for metal oxides
Source: RSC Adv. 2020 Jun 19;10(39):23484–90. doi: 10.1039/d0ra03696j (PMC9054895; doi:10.1039/d0ra03696j)
Supplement: RA-010-D0RA03696J-s001 [file RA-010-D0RA03696J-s001.pdf]

Electronic Supplementary Material (ESI) for RSC Advances.
This journal is © The Royal Society of Chemistry 2020

RSC Advances

Electronic Supplementary Information (ESI)

Effects of thiol substitution in low-transition-temperature mixtures (LTTMs) as solvents for metal oxides

Giacomo Damilano[a], Antero Laitinen[b], Pia Willberg-Keyriläinen[b], Tiina Lavonen[b], Riina Häkkinen[b], Wim Dehaen[a], Koen Binnemans[a], and Lauri Kuutti[b]*

[a.] *KU Leuven, Department of Chemistry, Celestijnenlaan 200F - P. O. Box 2404, B-3001 Leuven, Belgium.*
[b.] *VTT Technical Research Centre of Finland Ltd, Tietotie 4E, FI-02150 VTT, Finland, +358 20 722 2936.*
* Corresponding author. E-mail: lauri.kuutti@vtt.fi

Please do not adjust margins

Index

Index 2
List of figures 3
List of tables 4
Chemicals and solvents 5
Materials and instrumentation 5
Experimental methods 5
  Synthesis of the DES. 5
  Assay for the dissolution of metal oxides in DES. 6
Visual report - Low temperature-transition mixtures formation 7
  ChCl:GA 7
  ChCl:TGA 7
  ChCl:LA 7
  ChCl:TLA 7
  ChCl:MA 8
  ChCl:TMA 8
  ChCl:DTT 8
Visual report – Metal dissolution in low temperature-transition mixtures 10
  ChCl:GA(1:2) 10
  ChCl:TGA(1:2) 11
  ChCl:LA(1:2) 12
  ChCl:TLA(1:2) 13
  ChCl:DTT(1:3) 14
Metal dissolution (ICP-OES results) 15
Physico-chemical characterisation 17
  ChCl:GA(1:2) 17
  ChCl:GA(1:3) 21
  ChCl:TGA(1:2) 23
  ChCl:TGA(1:3) 26
  ChCl:LA(1:2) 28
  ChCl:LA(1:3) 32
  ChCl:TLA(1:2) 34
  ChCl:TLA(1:3) 38
  ChCl:MA(1:1) 40
  ChCl:TMA(1:1) 43
  ChCl:DTT(1:2) 46
  ChCl:DTT(1:3) 48

Please do not adjust margins

Please do not adjust margins

List of figures

Figure S 1 Mixtures of choline chloride with glycolic acid. From left to right the choline chloride to glycolic acid molar ratio changes (3:1, 2:1, 1:1, 1:2, 1:3). The picture was taken after equilibration at room temperature. 8
Figure S 2 Mixtures of choline chloride with thioglycolic acid. From left to right the choline chloride to thioglycolic acid molar ratio changes (3:1, 2:1, 1:1, 1:2, 1:3). The picture was taken after equilibration at room temperature. 8
Figure S 3 Mixtures of choline chloride with lactic acid. From left to right the choline chloride to lactic acid molar ratio changes (3:1, 2:1, 1:1, 1:2, 1:3). The picture was taken after equilibration at room temperature. 8
Figure S 4 Mixtures of choline chloride with thiolactic acid. From left to right the choline chloride to thiolactic acid molar ratio changes (3:1, 2:1, 1:1, 1:2, 1:3). The picture was taken after equilibration at room temperature. 9
Figure S 5 Mixtures of choline chloride with malic acid. From left to right the choline chloride to malic acid molar ratio changes (3:1, 2:1, 1:1, 1:2, 1:3). The picture was taken after equilibration at room temperature. 9
Figure S 6 Mixtures of choline chloride with thiomalic acid. From left to right the choline chloride to thiomalic acid molar ratio changes (3:1, 2:1, 1:1, 1:2, 1:3). The picture was taken after equilibration at room temperature. 9
Figure S 7 Mixtures of choline chloride with dithiotreitol. From left to right the choline chloride to dithiotreitol molar ratio changes (3:1, 2:1, 1:1, 1:2, 1:3).). The picture was taken after equilibration at room temperature. 10
Figure S 8. Metal dissolution process in ChCl:GA (1:2). [A] Photo after ~24h after beginning the dissolution process, [B] Photo of the centrifuged sample after 48h [C] Photo of the filtrate. From left to right: CdO, CoO, $LiCoO_2$, Cu_2O, Fe_3O_4, NiO, ZnO, AgO, Au_2O_3, CuO, FeO, Fe_2O_3, PbO. Pictures were taken after equilibration at room temperature. 11
Figure S 9. Metal dissolution process in ChCl:TGA (1:2). [A] Photo after ~24h after beginning the dissolution process, [B] Photo of the centrifuged sample after 48h [C] Photo of the filtrate. From left to right: CdO, CoO, $LiCoO_2$, Cu_2O, Fe_3O_4, NiO, ZnO, AgO, Au_2O_3, CuO, FeO, Fe_2O_3, PbO. Pictures were taken after equilibration at room temperature. 12
Figure S 10. Metal dissolution process in ChCl:LA (1:2). [A] Photo after ~24h after beginning the dissolution process, [B] Photo of the centrifuged sample after 48h [C] Photo of the filtrate. From left to right: CdO, CoO, $LiCoO_2$, Cu_2O, Fe_3O_4, NiO, ZnO, AgO, Au_2O_3, CuO, FeO, Fe_2O_3, PbO. Pictures were taken after equilibration at room temperature. 13
Figure S 11. Metal dissolution process in ChCl:TLA (1:2). [A] Photo after ~24h after beginning the dissolution process, [B] Photo of the centrifuged sample after 48h [C] Photo of the filtrate. From left to right: CdO, CoO, $LiCoO_2$, Cu_2O, Fe_3O_4, NiO, ZnO, AgO, Au_2O_3, CuO, FeO, Fe_2O_3, PbO. Pictures were taken after equilibration at room temperature. 14
Figure S 12. Metal dissolution process in ChCl:DTT (1:3). [A] Photo after ~24h after beginning the dissolution process, [B] Photo of the centrifuged sample after 48h [C] Photo of the filtrate. From left to right: CdO, CoO, $LiCoO_2$, Cu_2O, Fe_3O_4, NiO, ZnO, AgO, Au_2O_3, CuO, FeO, Fe_2O_3, PbO. Pictures were taken after equilibration at room temperature. 15
Figure S 13. Grouped barchart representation of the ICP-OES results for the microwave sample digestion in *aqua regia* 16
Figure S 14. Grouped barchart representation of the ICP-OES results for the microwave sample dilution in an aqueous solution of niric acid (1 %). 17
Figure S 15. ^1H NMR of ChCl:GA(1:2) in deuterated water. 18
Figure S 16. IR spectra of ChCl:GA(1:2). 18
Figure S 17. IR spectra overlap of ChCl:GA (black solid line), GA (grey dashed line), and ChCl (light grey dotted line). 19
Figure S 18. UV spectra of ChCl:GA(1:2). 19
Figure S 19. DSC spectra of ChCl:GA(1:2) 20
Figure S 20. Viscosity measurements for ChCl:GA(1:2). 21
Figure S 21. ^1H NMR of ChCl:GA(1:3) in deuterated water. 22
Figure S 22. IR spectra of ChCl:GA(1:3). 22
Figure S 23. DSC spectra of ChCl:GA(1:3) 23
Figure S 24. ^1H NMR of ChCl:TGA(1:2) in deuterated water. 24
Figure S 25. IR spectra of ChCl:TGA(1:2). 24
Figure S 26. IR spectra overlap of ChCl:TGA (black solid line), TGA (grey dashed line), and ChCl (light grey dotted line). 25
Figure S 27. UV spectra of ChCl:TGA(1:2). 25
Figure S 28. DSC spectra of ChCl:TGA(1:2) 26
Figure S 29. ^1H NMR of ChCl:TGA(1:3) in deuterated water. 27
Figure S 30. IR spectra of ChCl:TGA(1:3). 27
Figure S 31. DSC spectra of ChCl:TGA(1:3) 28
Figure S 32. ^1H NMR of ChCl:LA(1:2) in deuterated water. 29
Figure S 33. IR spectra overlap of ChCl:LA (black solid line), LA (grey dashed line), and ChCl (light grey dotted line). 29

Please do not adjust margins

Please do not adjust margins

Figure S 34. IR spectra of ChCl:LA(1:2).....30
Figure S 35. UV spectra of ChCl:LA(1:2).....30
Figure S 36. DSC spectra of ChCl:LA(1:2).....31
Figure S 37. Viscosity measurements for ChCl:LA(1:2).....32
Figure S 38. ^{1}H NMR of ChCl:LA(1:3) in deuterated water.....33
Figure S 39. IR spectra of ChCl:LA(1:3).....33
Figure S 40. DSC spectra of ChCl:LA(1:3).....34
Figure S 41. ^{1}H NMR of ChCl:TLA(1:2) in deuterated water.....35
Figure S 42. IR spectra of ChCl:TLA(1:2).....35
Figure S 43. IR spectra overlap of ChCl:TLA (black solid line), TLA (grey dashed line), and ChCl (light grey dotted line).....36
Figure S 44. UV spectra of ChCl:TLA(1:2).....36
Figure S 45. DSC spectra of ChCl:TLA(1:2).....37
Figure S 46. Viscosity measurements for ChCl:TLA(1:2).....38
Figure S 47. ^{1}H NMR of ChCl:TLA(1:3) in deuterated water.....39
Figure S 48. IR spectra of ChCl:TLA(1:3).....39
Figure S 49. DSC spectra of ChCl:TLA(1:3).....40
Figure S 50. ^{1}H NMR of ChCl:MA(1:1) in deuterated water.....41
Figure S 51. IR spectra of ChCl:MA(1:1).....41
Figure S 52. DSC spectra of ChCl:MA(1:1).....42
Figure S 53. Viscosity measurements for ChCl:MA(1:1).....43
Figure S 54. ^{1}H NMR of ChCl:TMA(1:1) in deuterated water.....44
Figure S 55. IR spectra of ChCl:TMA(1:1).....44
Figure S 56. DSC spectra of ChCl:TMA(1:1).....45
Figure S 57. Viscosity measurements for ChCl:TMA(1:1).....46
Figure S 58. ^{1}H NMR of ChCl:DTT(1:2) in deuterated water.....47
Figure S 59. IR spectra of ChCl:DTT(1:2).....47
Figure S 60. IR spectra overlap of ChCl:DTT (black solid line), DTT (grey dashed line), and ChCl (light grey dotted line).....48
Figure S 61. DSC spectra of ChCl:DTT(1:2).....48
Figure S 62. ^{1}H NMR of ChCl:DTT(1:3) in deuterated water.....49
Figure S 63. IR spectra of ChCl:DTT(1:3).....49
Figure S 64. UV spectra of ChCl:DTT(1:3).....50
Figure S 65. DSC spectra of ChCl:DTT(1:3).....50
Figure S 66. Viscosity measurements for ChCl:DTT(1:3).....51

List of tables

Table S 1. Visual observation of the minimum conditions required to the formation of the DES under magnetic stirring.....5
Table S 2. ICP-OES results for the microwave sample digestion in *aqua regia*. The metal content is reported as an unit of concentration (g L^{-1}). The details about the sample pre-treatment are listed in the methodology section.....15
Table S 3. ICP-OES results for the microwave sample dilution in an aqueous solution of niric acid (1 %). The metal content is reported as an unit of concentration (g L^{-1}). The details about the sample pre-treatment are listed in the methodology section.....16

Please do not adjust margins

Please do not adjust margins

Chemicals and solvents

Choline chloride (ChCl) (≥98%, 67-48-1), DL-malic acid (MA) (≥98%, 6915-15-7), DL-thiomalic acid (TMA) (>98.0%, 70-49-5), DL-lactic acid (LA) (90% solution in water, 50-21-5), DL-thiolactic acid (TLA) (>97.0%, 79-42-5), glycolic acid (GA) (≥99%, 79-14-1), thioglycolic acid (TGA) (≥98%, 68-11-1), cadmium(II) oxide (99.5%, 1306-19-0), cobalt(II) oxide (99.99%, 1307-96-6), nickel(II) oxide (99.8%, 1313-99-1), lead(II) oxide (99.999%, 1317-36-8), iron(II) oxide (99.7%, 1345-25-1), iron(II,III) mixed oxide (98%, 1309-37-1), zinc(II) oxide (99.99%, 1314-13-2) were purchased from Sigma-Aldrich (Espoo, Finland). DL-Dithiothreitol (DTT) (>99%, 3483-12-3) was purchased from Apollo Scientific (Manchester, United Kingdom). Lithium cobalt(III) oxide (≥98%, 12190-79-3) and gold(III) oxide (99% , 1303-58-8) were purchased from STREM (Helsinki, Finland). Copper(II) oxide (≥97.5%, 1317-39-1) was purchased from VWR (Helsinki, Finland). Copper(I) oxide (99.7%, 1317-39-1), and silver(I) oxide (>99.0%, 20667-12-3) were purchased from Fluka AG (Bucks, Switzerland). Choline chloride and lactic acid were dried prior to use. Choline chloride was dried at high vacuum for at least 24 h prior to use. All the other chemicals were used without further purification.

Materials and instrumentation

Differential scanning calorimetry (DSC) analyses was carried out on a DSC apparatus (Mettler-Toledo DSC820 STARe System, Switzerland) in the temperature range from -120 to 25 °C and a heating rate of 10 °C min^{-1}. Rheological analysis were carried out with a cone/plate rheometer (Anton-Paar Physica MCR 301, Germany) equipped with a conic spindle (CP50-2/TG). The instrument was equipped with a thermal control unit to allow the investigation at temperatures between 25 and 60 °C. The viscosity was measured in triplicates as a function of the shear rate. NMR measurements were performed on a NMR spectrometer (Bruker Avance III HD) operating at a ^1H frequency of 500 MHz at 22 °C. Fourier transform infrared (FTIR) measurements were carried out using a FTIR spectrometer (Thermo Fisher Nicolet iS50, USA) and recorded on a Diamond-ATR (attenuated total reflectance) module with a resolution of 2 cm^{-1}. UV-vis spectra were carried out on a UV-vis double beam spectrophotometer (Shimadzu UV-2600, Japan) with quartz cuvettes (10 x 10 mm). Solution samples were assayed for Ag, Au, Co, Cd, Cu, Fe, Ni, Pb, and Zn content on an inductively coupled plasma optical emission spectroscopy (ICP-OES) apparatus (5100 SVDV, Agilent Technologies). ICP-OES measurements were performed using multi-element standards (Inorganic Ventures and SPEX) for calibration lines and control samples. Centrifugation was carried out on a refrigerated centrifuge (Eppendorf 5804 R) at 4000 and 40 °C rpm for 45 min. For the estimation of the pH, pH-indicator strips (ColorpHast, Merck) were used with a sensitivity of 1 unit over a pH range from 0 to 14. Disposable syringe filters (Whatman, PVDF membrane in PP housing, 25 mm Ø, 0.45 µm pore Ø) were used for sample filtration.

Experimental methods

Synthesis of the DES.

If the HBA had a thiol functionality, all of the procedures were run under nitrogen atmosphere. In a 5mL closed vial the HBD:HBA mixture was stirred with a magnetic stirrer for 2 h at room temperature, after which the phase aspect was recorded (Table S1). Then, the mixture was stirred for 2 h at 60°C, after which the phase aspect was newly recorded (Table S1). If a homogeneous liquid was obtained, the liquid mixture was characterised via IR, UV, NMR, TGA, DSC and a cone plate rheometer.

The preparation of the DESs was repeated at a decigram scale. The homogeneization time was slightly extended due to the inability to have a comparable stirring strength.

Please do not adjust margins

Please do not adjust margins

Assay for the dissolution of metal oxides in DES.

The solubility of various metal oxides (i.e. Ag_2O, Au_2O_3, CdO, CoO, $LiCoO_2$, CuO, Cu_2O, FeO, Fe_2O_3, NiO, PbO, ZnO) in the synthesised DES (i.e. ChCl:GA (1:2), ChCl:TGA (1:2), ChCl:LA (1:2), ChCl:TLA (1:2)) was tested. Each vial was equipped with a magnetic stirrer (VWR, PTFE coated magnetic bar, 10 x 3 mm) and filled with one of the metal oxides in analysis (about 100 mg) and one of the DES in analysis (about 2 mL). The samples were stirred for 48 h at 50° C. About every 8 h the vials were checked for the oxide dissolution. If full dissolution was observed, additional oxide was added to the vial (reaching up to about 500 mg of metal oxide).

After 48 h, the sample was centrifuged at a speed of 2500 rpm at 40°C for 50 minutes. The centrifuged samples were then filtered *via* the use of syringe filters with a pore size of 45 µm. Samples were diluted 1:100 or 1:1000 with 1% HNO_3 solution (optima grade). If a precipitate was formed after dilution, the samples were centrifuged. The supernatant was then collected and analysed. Because of the precipitation, samples were re-tested after being pre-processed via a microwave digester. About 50mg of the solution sample was weighted in a PTFE lined microwave digestion tubes and digested with a few mL of reverse *aqua regia* (ultra-pure grade). The digested samples were diluted to 20mL with milliQ water and measured without further dilution. The samples were then measured by ICP-MS. The remaining filtrate was diluted (1:100) in the same DES solvent. The diluted solution was then recorded three times *via* a UV-vis spectrometer in the range between 200 and 700 nm.

Table S 1. Visual observation of the minimum conditions required to the formation of the DES under magnetic stirring.

HBA:HBD (molar ratio)	Appearance at 20-22 °C	t (h)	T (°C)
ChCl:GA (3:1)	glass / solid	2	20-22
ChCl:GA (2:1)	glass / solid	2	20-22
ChCl:GA (1:1)	non-homogenous liquid	2	20-22
ChCl:GA (1:2)	clear homogeneous liquid	2	20-22
ChCl:GA (1:3) [1]	clear homogenous liquid	2	20-22
ChCl:TGA (3:1)	glass / solid	2	20-22
ChCl:TGA (2:1)	glass / solid	2	20-22
ChCl:TGA (1:1)	non-homogenous liquid	2	20-22
ChCl:TGA (1:2)	clear homogenous liquid	2	20-22
ChCl:TGA (1:3)	clear homogenous liquid	2	20-22
ChCl:LA (3:1)	glass / solid	2	20-22
ChCl:LA (2:1)	glass / solid	2	20-22
ChCl:LA (1:1)	non-homogenous liquid	2	20-22
ChCl:LA (1:2)	clear homogenous liquid	2	20-22
ChCl:LA (1:3)	clear homogenous liquid	2	20-22
ChCl:TLA (3:1)	glass / solid	2	20-22
ChCl:TLA (2:1)	glass / solid	2	20-22
ChCl:TLA (1:1)	non-homogenous liquid	2	20-22
ChCl:TLA (1:2)	clear homogenous liquid	2	20-22
ChCl:TLA (1:3)	clear homogenous liquid	2	20-22
ChCl:MA (3:1)	glass / solid	2	60-70
ChCl:MA (2:1)	glass / solid	2	60-70
ChCl:MA (1:1)	clear homogenous liquid	2	60-70
ChCl:MA (1:2)	non-homogenous liquid	2	60-70
ChCl:MA (1:3)	glass / solid	2	60-70
ChCl:TMA (3:1)	glass / solid	4	60-70
ChCl:TMA (2:1)	glass / solid	4	60-70
ChCl:TMA (1:1)	clear faint yellow homogenous liquid	4	60-70
ChCl:TMA (1:2)	glass / solid	4	60-70
ChCl:TMA (1:3)	glass / solid	4	60-70
ChCl:DTT (3:1)	glass / solid	3	20-22
ChCl:DTT (2:1)	glass / solid	3	20-22
ChCl:DTT (1:1)	non-homogenous liquid	3	20-22
ChCl:DTT (1:2)	clear homogenous liquid	3	20-22
ChCl:DTT (1:3)	clear homogenous liquid	3	20-22

[1] A precipitate was observed after refrigerating the sample overnight at 6 °C.

Please do not adjust margins

Please do not adjust margins

Visual report - Low temperature-transition mixtures formation

ChCl:GA

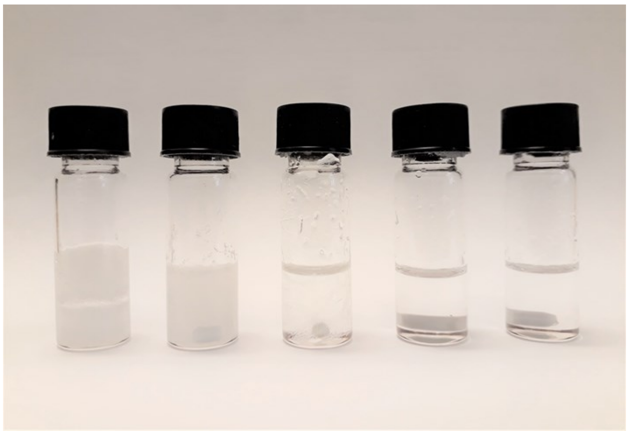

Figure S 1 Mixtures of choline chloride with glycolic acid. From left to right the choline chloride to glycolic acid molar ratio changes (3:1, 2:1, 1:1, 1:2, 1:3). The picture was taken after equilibration at room temperature.

ChCl:TGA

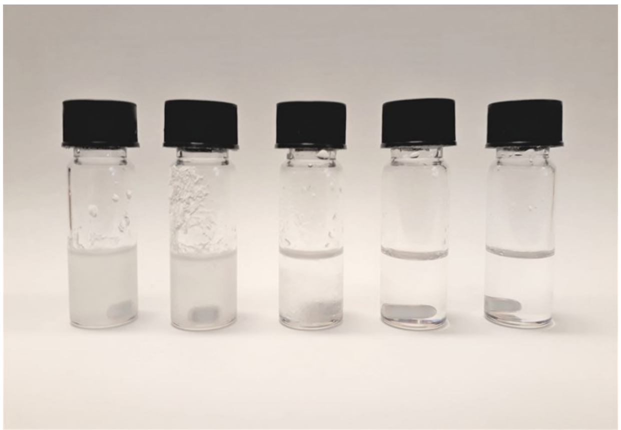

Figure S 2 Mixtures of choline chloride with thioglycolic acid. From left to right the choline chloride to thioglycolic acid molar ratio changes (3:1, 2:1, 1:1, 1:2, 1:3). The picture was taken after equilibration at room temperature.

ChCl:LA

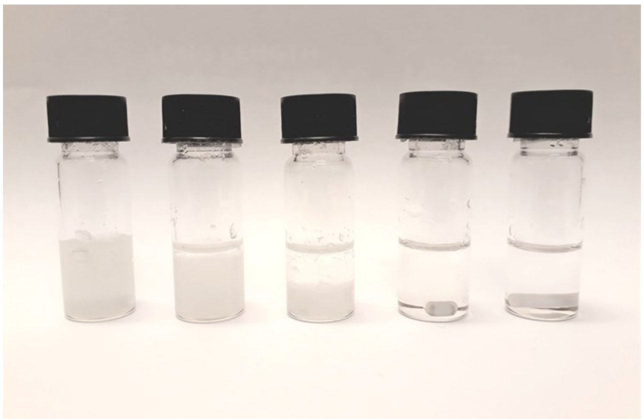

Figure S 3 Mixtures of choline chloride with lactic acid. From left to right the choline chloride to lactic acid molar ratio changes (3:1, 2:1, 1:1, 1:2, 1:3). The picture was taken after equilibration at room temperature.

ChCl:TLA

Please do not adjust margins

Please do not adjust margins

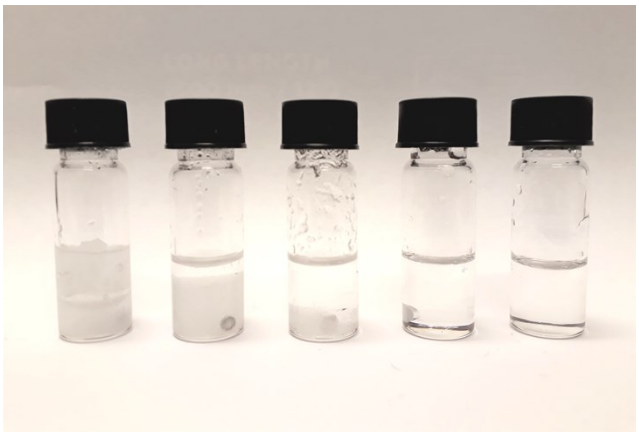

Figure S 4 Mixtures of choline chloride with thiolactic acid. From left to right the choline chloride to thiolactic acid molar ratio changes (3:1, 2:1, 1:1, 1:2, 1:3). The picture was taken after equilibration at room temperature.

ChCl:MA

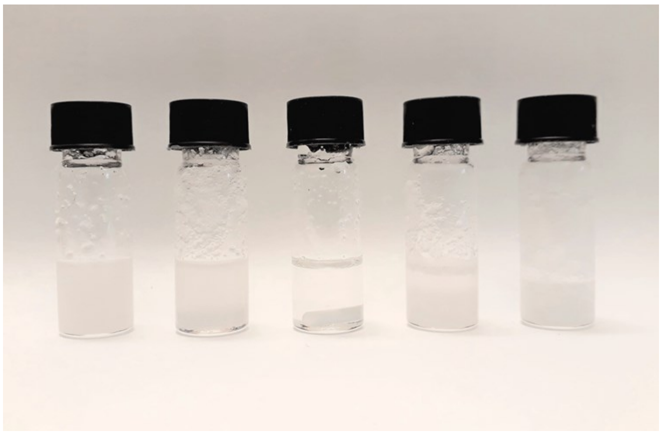

Figure S 5 Mixtures of choline chloride with malic acid. From left to right the choline chloride to malic acid molar ratio changes (3:1, 2:1, 1:1, 1:2, 1:3). The picture was taken after equilibration at room temperature.

ChCl:TMA

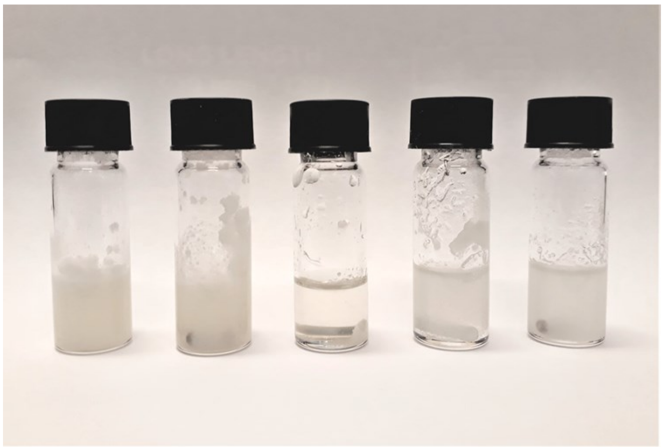

Figure S 6 Mixtures of choline chloride with thiomalic acid. From left to right the choline chloride to thiomalic acid molar ratio changes (3:1, 2:1, 1:1, 1:2, 1:3). The picture was taken after equilibration at room temperature.

ChCl:DTT

Please do not adjust margins

Please do not adjust margins

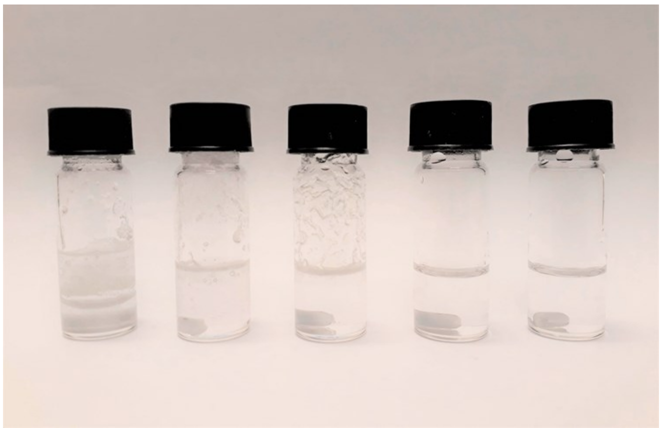

Figure S 7 Mixtures of choline chloride with dithiotreitol. From left to right the choline chloride to dithiotreitol molar ratio changes (3:1, 2:1, 1:1, 1:2, 1:3).). The picture was taken after equilibration at room temperature.

Please do not adjust margins

Please do not adjust margins

Visual report – Metal dissolution in low temperature-transition mixtures

ChCl:GA(1:2)

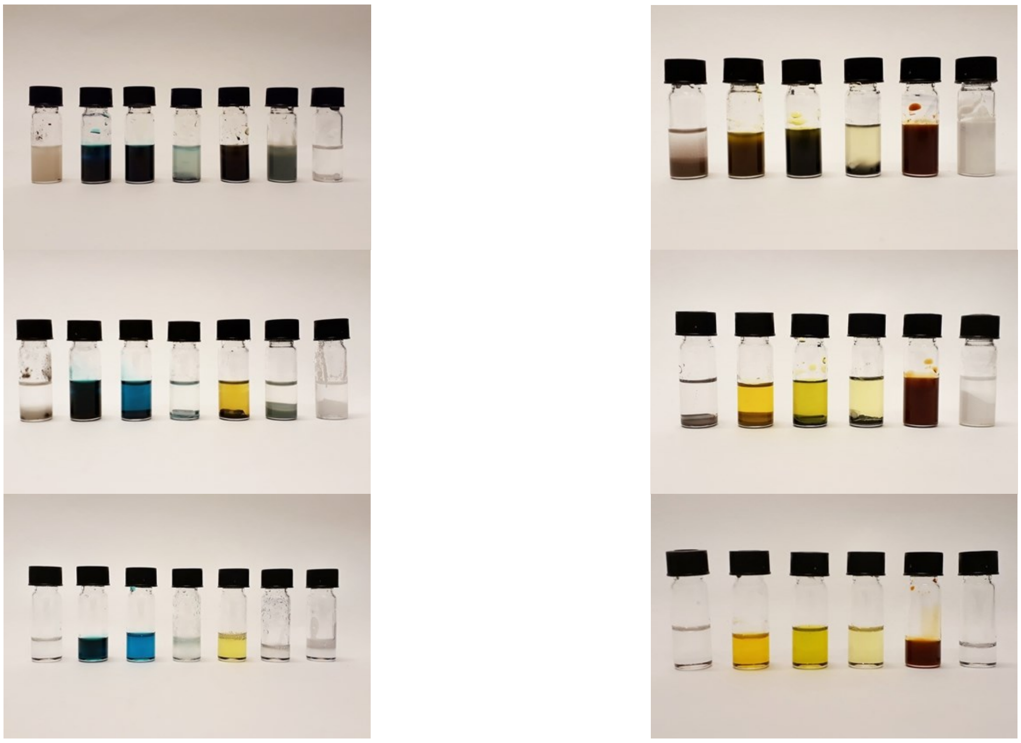

[A]

[B]

[C]

Figure S 8. Metal dissolution process in ChCl:GA (1:2). [A] Photo after ~24h after beginning the dissolution process, [B] Photo of the centrifuged sample after 48h [C] Photo of the filtrate. From left to right: CdO, CoO, $LiCoO_2$, Cu_2O, Fe_3O_4, NiO, ZnO, AgO, Au_2O_3, CuO, FeO, Fe_2O_3, PbO. Pictures were taken after equilibration at room temperature.

Please do not adjust margins

Please do not adjust margins

ChCl:TGA(1:2)

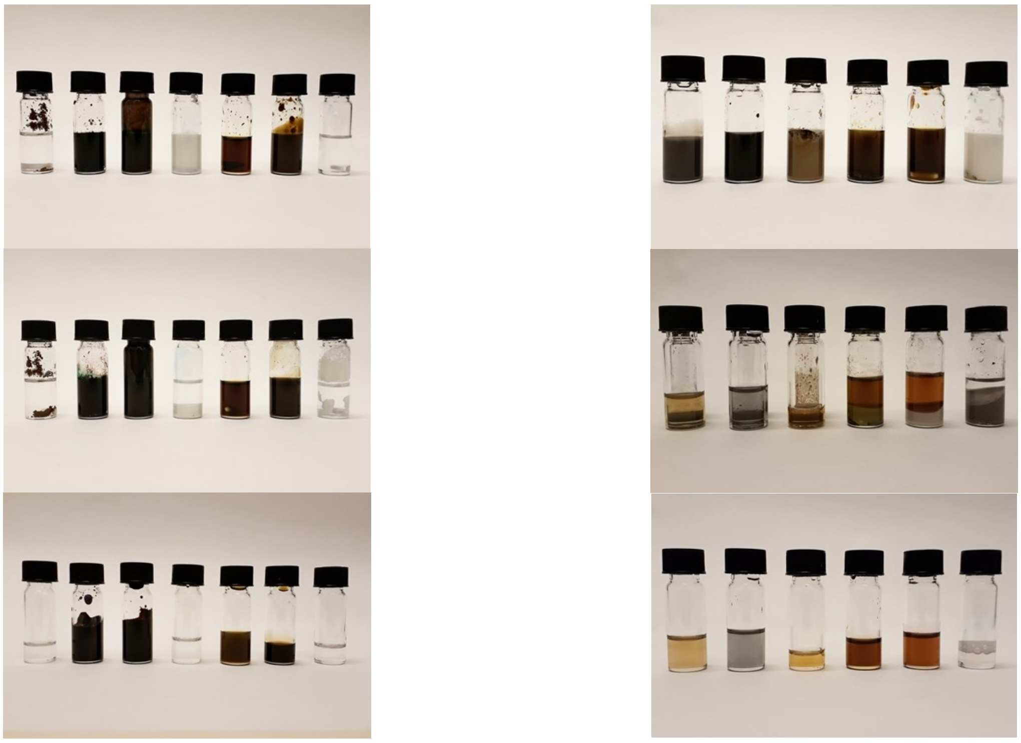

[A]

[B]

[C]

Figure S 9. Metal dissolution process in ChCl:TGA (1:2). [A] Photo after ~24h after beginning the dissolution process, [B] Photo of the centrifuged sample after 48h [C] Photo of the filtrate. From left to right: CdO, CoO, $LiCoO_2$, Cu_2O, Fe_3O_4, NiO, ZnO, AgO, Au_2O_3, CuO, FeO, Fe_2O_3, PbO. Pictures were taken after equilibration at room temperature.

Please do not adjust margins

Please do not adjust margins

ChCl:LA(1:2)

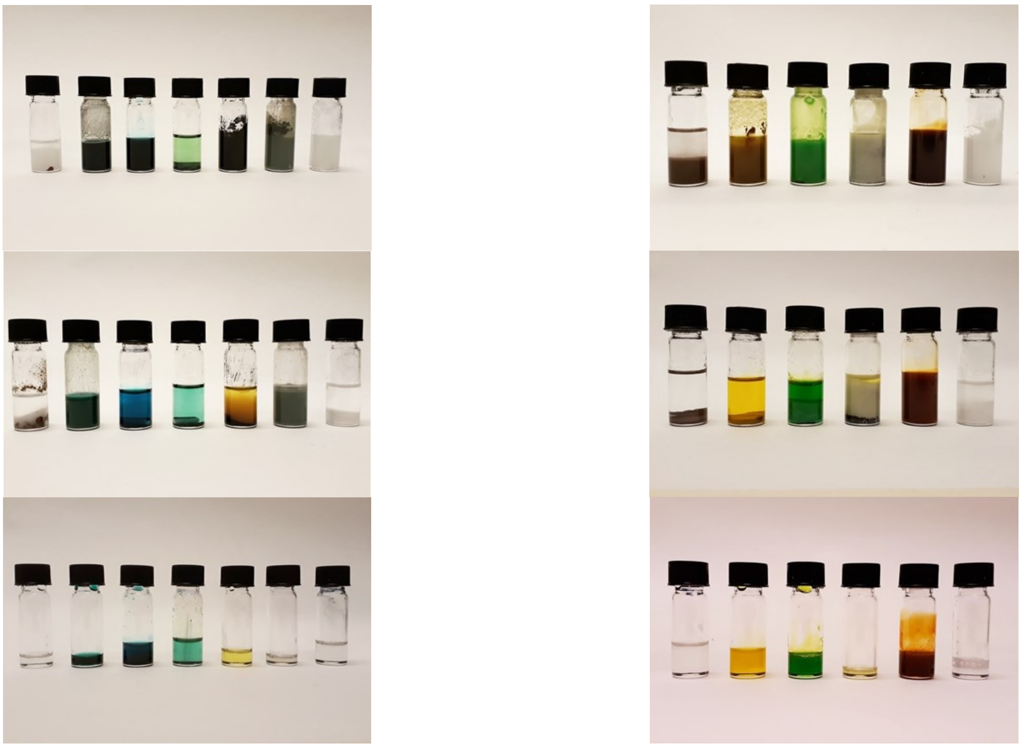

[A]

[B]

[C]

Figure S 10. Metal dissolution process in ChCl:LA (1:2). [A] Photo after ~24h after beginning the dissolution process, [B] Photo of the centrifuged sample after 48h [C] Photo of the filtrate. From left to right: CdO, CoO, $LiCoO_2$, Cu_2O, Fe_3O_4, NiO, ZnO, AgO, Au_2O_3, CuO, FeO, Fe_2O_3, PbO. Pictures were taken after equilibration at room temperature.

Please do not adjust margins

Please do not adjust margins

ChCl:TLA(1:2)

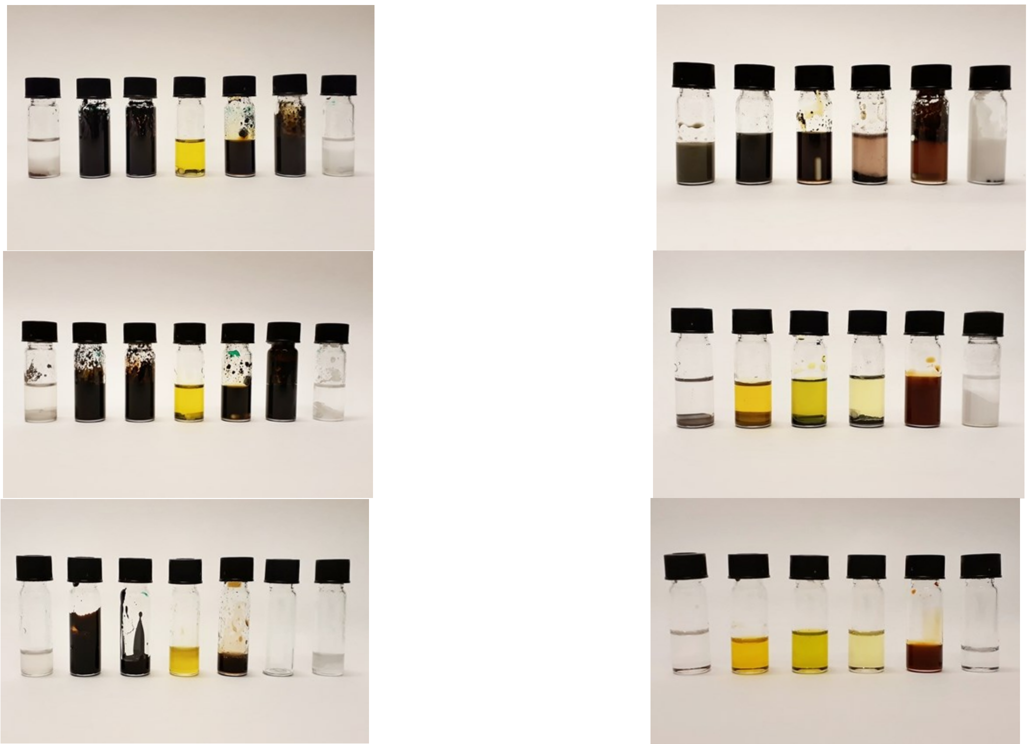

[A]

[B]

[C]

Figure S 11. Metal dissolution process in ChCl:TLA (1:2). [A] Photo after ~24h after beginning the dissolution process, [B] Photo of the centrifuged sample after 48h [C] Photo of the filtrate. From left to right: CdO, CoO, $LiCoO_2$, Cu_2O, Fe_3O_4, NiO, ZnO, AgO, Au_2O_3, CuO, FeO, Fe_2O_3, PbO. Pictures were taken after equilibration at room temperature.

Please do not adjust margins

Please do not adjust margins

ChCl:DTT(1:3)

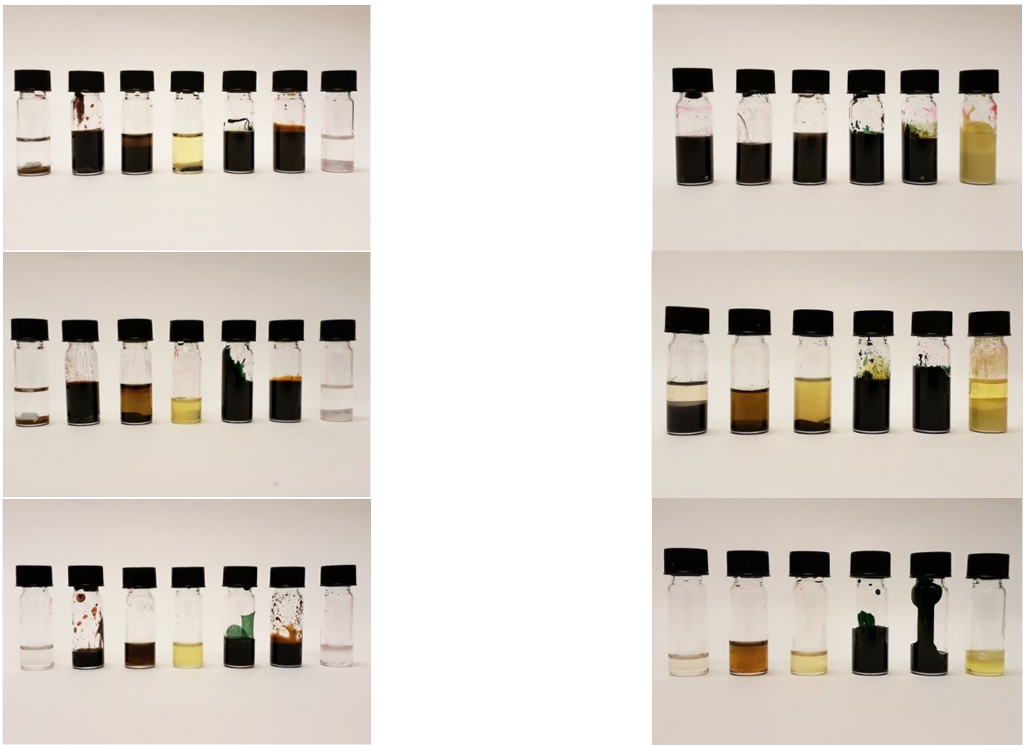

[A]

[B]

[C]

Figure S 12. Metal dissolution process in ChCl:DTT (1:3). [A] Photo after ~24h after beginning the dissolution process, [B] Photo of the centrifuged sample after 48h [C] Photo of the filtrate. From left to right: CdO, CoO, $LiCoO_2$, Cu_2O, Fe_3O_4, NiO, ZnO, AgO, Au_2O_3, CuO, FeO, Fe_2O_3, PbO. Pictures were taken after equilibration at room temperature.

Please do not adjust margins

Metal dissolution (ICP-OES results)

Table S 2. ICP-OES results for the microwave sample digestion in *aqua regia*. The metal content is reported as an unit of concentration (g L^{-1}). The details about the sample pre-treatment are listed in the methodology section.

	Au2O3	CdO	CoO	Ag2O
**ChCl:GA (1:2)**	42.22 ± 0.99	96.96 ± 5.18	6.97 ± 0.07	0.62 ± 0.00
**ChCl:TGA (1:2)**	0.03 ± 0.00	213.31 ± 5.19	43.98 ± 0.23	0.67 ± 0.01
**ChCl:LA (1:2)**	31.22 ± 0.08	42.09 ± 3.08	8.30 ± 0.04	0.58 ± 0.00
**ChCl:TLA (1:2)**	0.05 ± 0.00	93.75 ± 2.23	0.35 ± 0.01	0.63 ± 0.01
**ChCl:DTT (1:3)**	3.19 ± 0.24	12.67 ± 0.30	1.46 ± 0.22	0.27 ± 0.00
	Cu2O	CuO	FeO	Fe2O3
**ChCl:GA (1:2)**	84.18 ± 3.15	3.11 ± 0.04	2.99 ± 0.08	27.90 ± 0.50
**ChCl:TGA (1:2)**	0.62 ± 0.01	0.63 ± 0.01	29.91 ± 0.06	23.79 ± 0.09
**ChCl:LA (1:2)**	77.16 ± 4.41	25.36 ± 3.00	3.86 ± 0.40	29.61 ± 1.58
**ChCl:TLA (1:2)**	83.99 ± 2.24	60.22 ± 0.50	20.21 ± 1.12	50.42 ± 1.04
**ChCl:DTT (1:3)**	2.93 ± 0.03	3.18 ± 0.06	9.89 ± 0.07	30.68 ± 1.45
	LiCoO2	NiO	PbO	ZnO
**ChCl:GA (1:2)**	3.50 ± 0.07	0.10 ± 0.00	0.82 ± 0.00	50.28 ± 3.76
**ChCl:TGA (1:2)**	22.11 ± 0.98	2.44 ± 0.08	0.51 ± 0.00	122.07 ± 9.28
**ChCl:LA (1:2)**	11.62 ± 0.14	0.27 ± 0.00	0.22 ± 0.01	13.52 ± 0.53
**ChCl:TLA (1:2)**	0.00 ± 0.00	0.00 ± 0.00	0.26 ± 0.00	90.95 ± 2.59
**ChCl:DTT (1:3)**	0.93 ± 0.08	4.51 ± 0.07	80.81 ± 3.87	21.85 ± 1.18

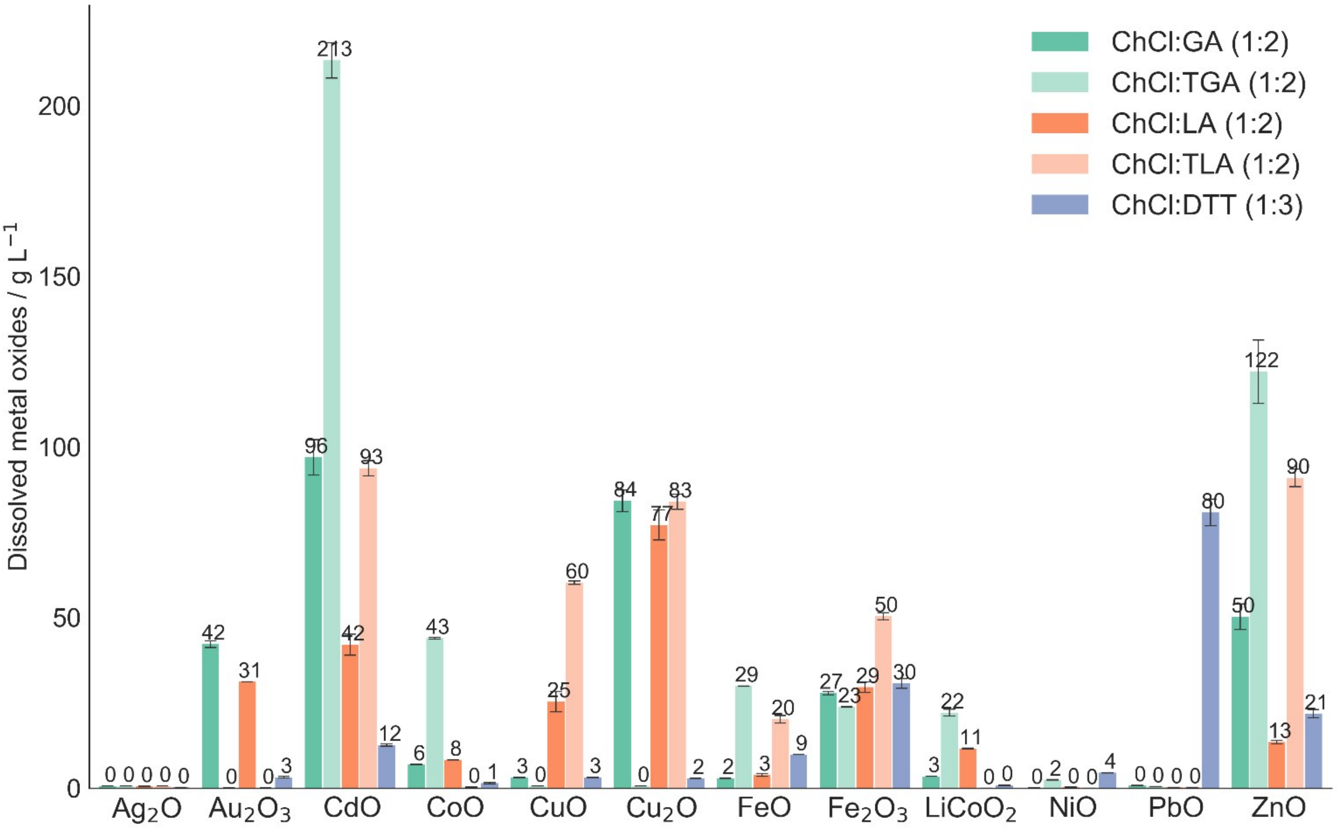

Figure S 13. Grouped barchart representation of the ICP-OES results for the microwave sample digestion in *aqua regia*

Please do not adjust margins

Table S 3. ICP-OES results for the microwave sample dilution in an aqueous solution of niric acid (1 %). The metal content is reported as an unit of concentration (g L^{-1}). The details about the sample pre-treatment are listed in the methodology section.

	Au2O3	CdO	CoO	Ag2O
**ChCl:GA (1:2)**	0.02 ± 0.00	127.54 ± 6.82	3.22 ± 0.03	0.06 ± 0.00
**ChCl:TGA (1:2)**	0.00 ± 0.00	237.25 ± 5.77	10.45 ± 0.05	0.05 ± 0.00
**ChCl:LA (1:2)**	0.02 ± 0.00	44.16 ± 3.23	7.47 ± 0.03	0.04 ± 0.00
**ChCl:TLA (1:2)**	0.02 ± 0.00	92.28 ± 2.20	0.29 ± 0.01	0.43 ± 0.01
**ChCl:DTT (1:3)**	0.01 ± 0.00	10.85 ± 0.26	0.01 ± 0.00	0.00 ± 0.00
	Cu2O	CuO	FeO	Fe2O3
**ChCl:GA (1:2)**	51.70 ± 1.94	3.06 ± 0.03	2.31 ± 0.07	24.99 ± 0.45
**ChCl:TGA (1:2)**	0.03 ± 0.00	0.07 ± 0.00	28.87 ± 0.06	21.10 ± 0.08
**ChCl:LA (1:2)**	77.32 ± 4.42	23.15 ± 2.74	3.34 ± 0.35	23.29 ± 1.24
**ChCl:TLA (1:2)**	14.05 ± 0.37	12.65 ± 3.48	16.40 ± 0.91	64.34 ± 16.92
**ChCl:DTT (1:3)**	0.00 ± 0.00	0.01 ± 0.00	8.57 ± 0.06	30.54 ± 1.44
	LiCoO2	NiO	PbO	ZnO
**ChCl:GA (1:2)**	3.25 ± 0.07	0.07 ± 0.00	0.78 ± 0.00	41.76 ± 3.12
**ChCl:TGA (1:2)**	8.11 ± 0.36	2.46 ± 0.08	0.27 ± 0.00	97.92 ± 7.44
**ChCl:LA (1:2)**	4.40 ± 0.05	0.17 ± 0.00	0.24 ± 0.02	9.15 ± 0.36
**ChCl:TLA (1:2)**	0.00 ± 0.00	0.00 ± 0.00	0.24 ± 0.07	78.46 ± 24.32
**ChCl:DTT (1:3)**	0.05 ± 0.00	2.34 ± 0.04	83.75 ± 4.01	16.95 ± 0.91

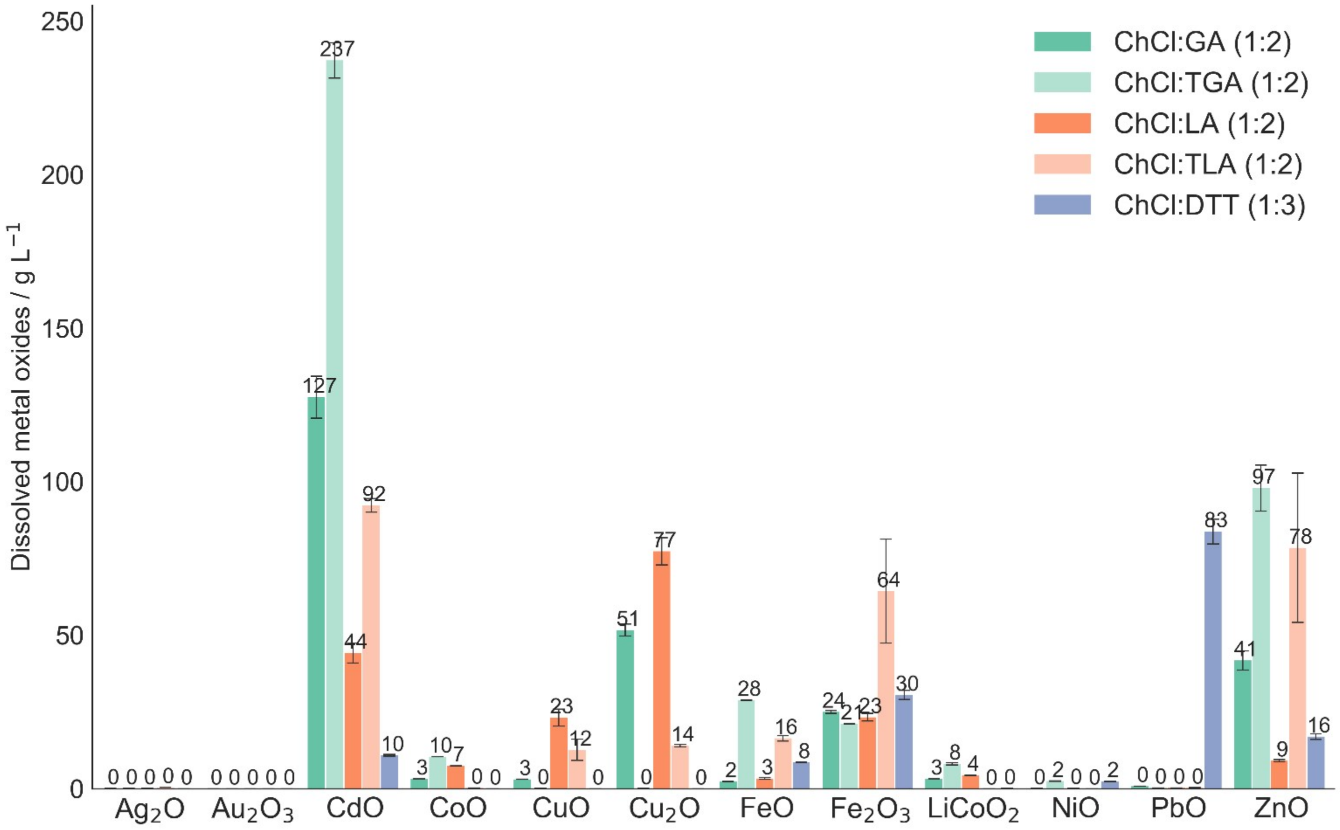

Figure S 14. Grouped barchart representation of the ICP-OES results for the microwave sample dilution in an aqueous solution of niric acid (1 %).

Physico-chemical characterisation

ChCl:GA(1:2)

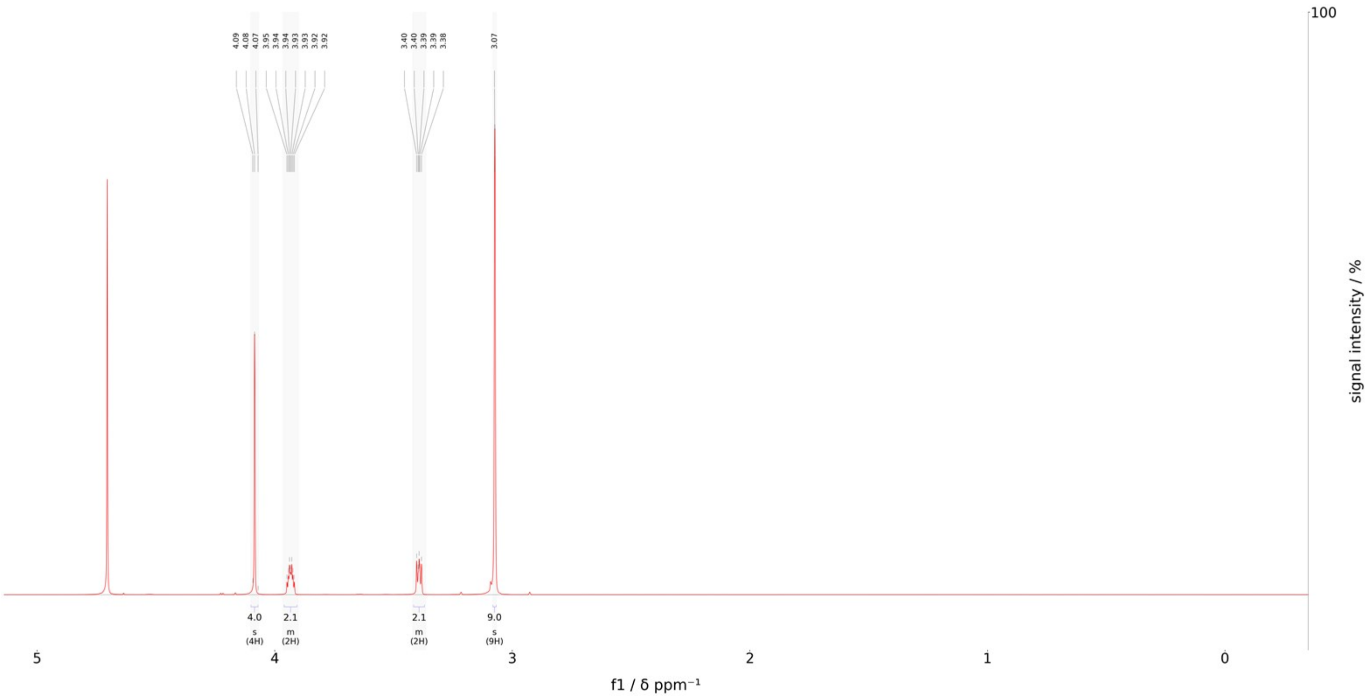

Figure S 15. ^{1}H NMR of ChCl:GA(1:2) in deuterated water.

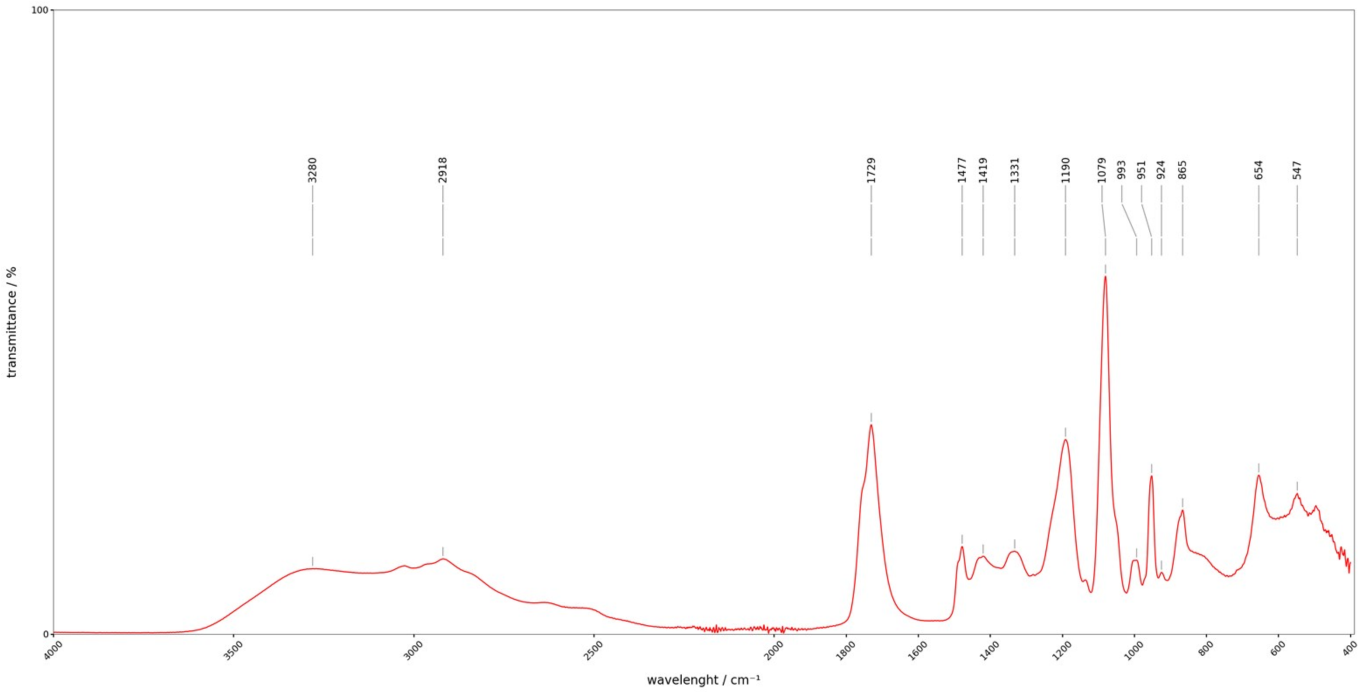

Figure S 16. IR spectra of ChCl:GA(1:2).

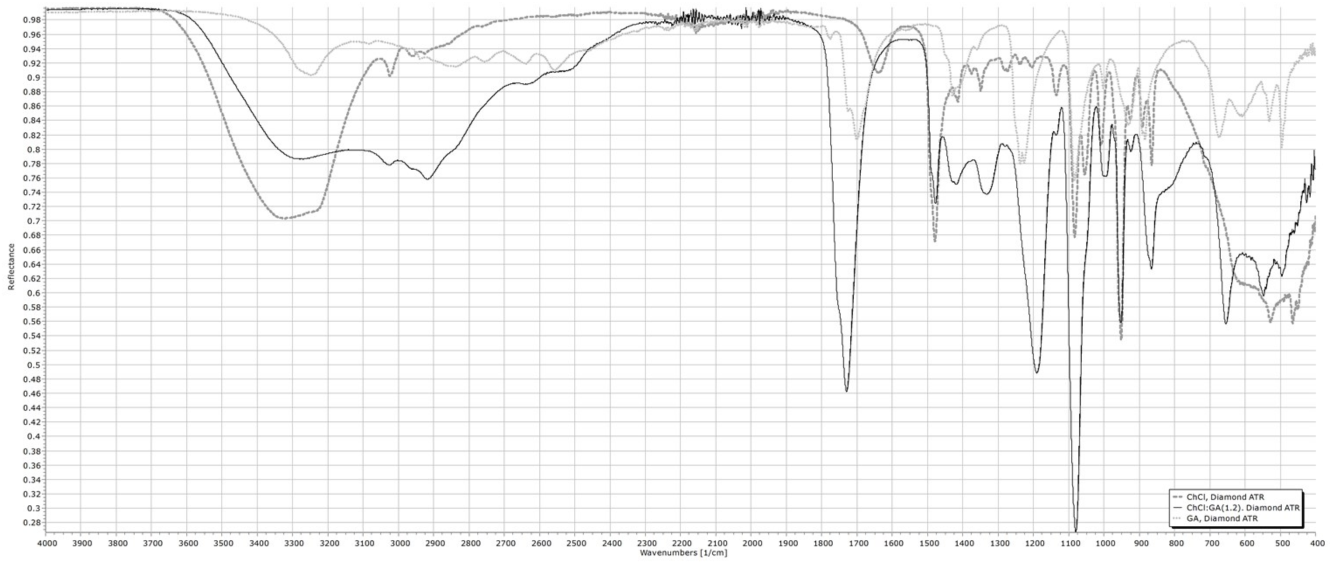

Figure S 17. IR spectra overlap of ChCl:GA (black solid line), GA (grey dashed line), and ChCl (light grey dotted line).

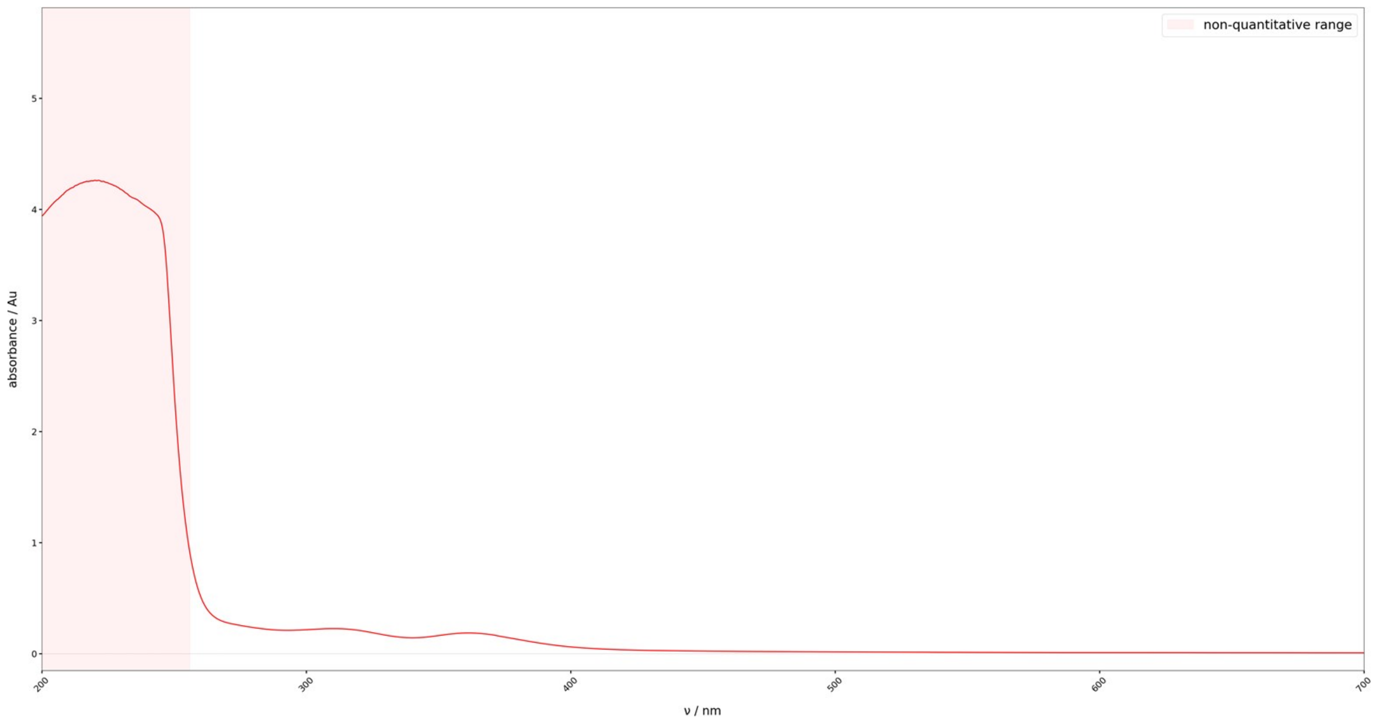

Figure S 18. UV spectra of ChCl:GA(1:2).

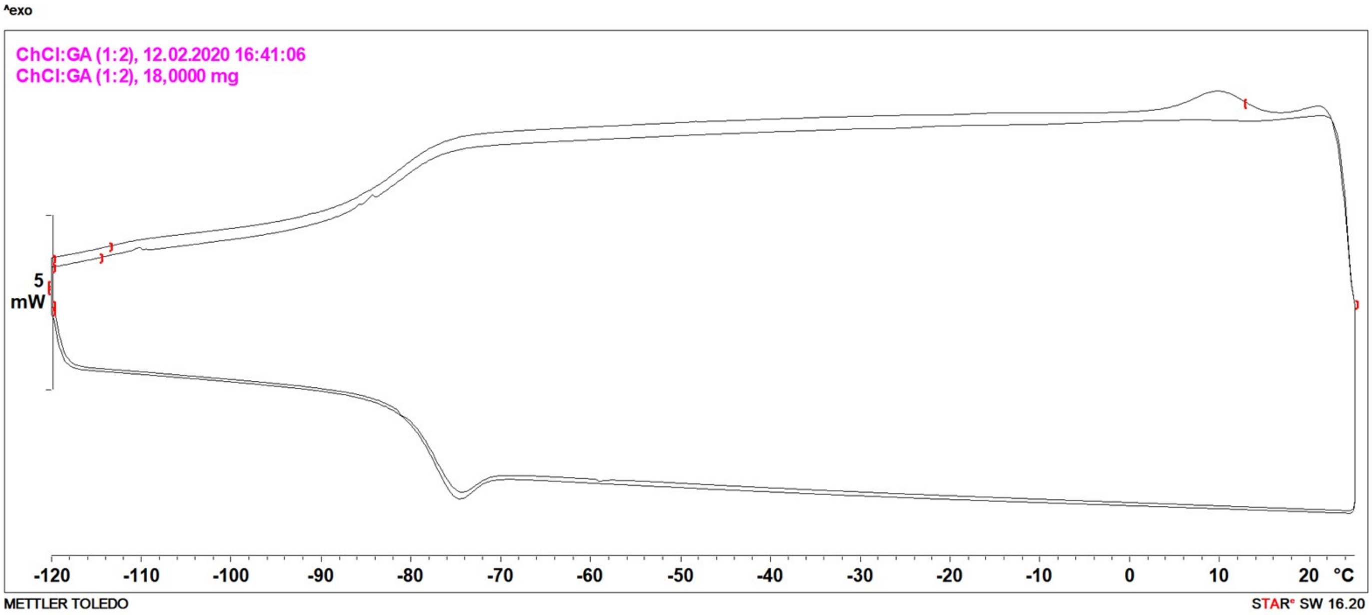

Figure S 19. DSC spectra of ChCl:GA(1:2).

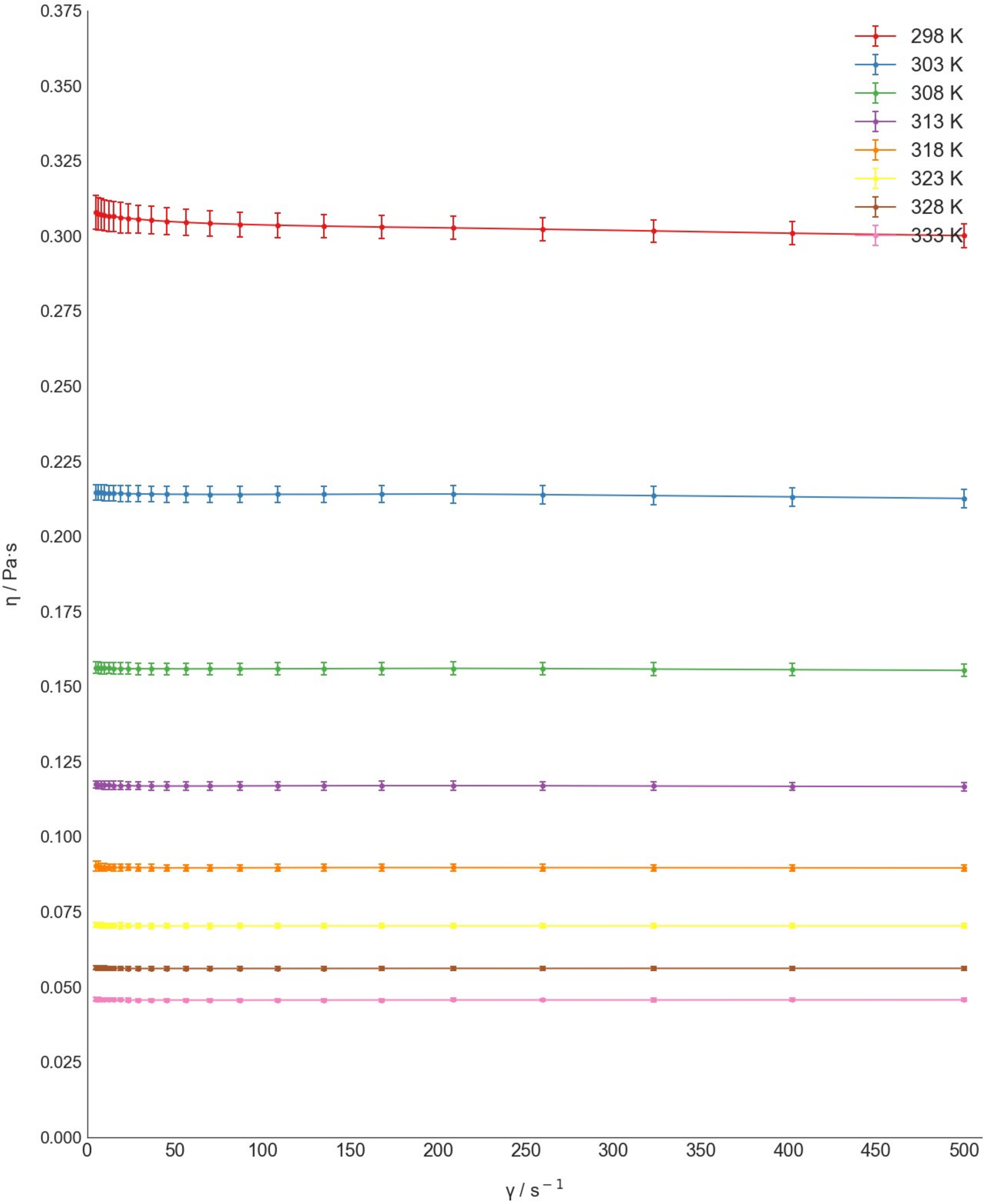

Figure S 20. Viscosity measurements for ChCl:GA(1:2).

ChCl:GA(1:3)

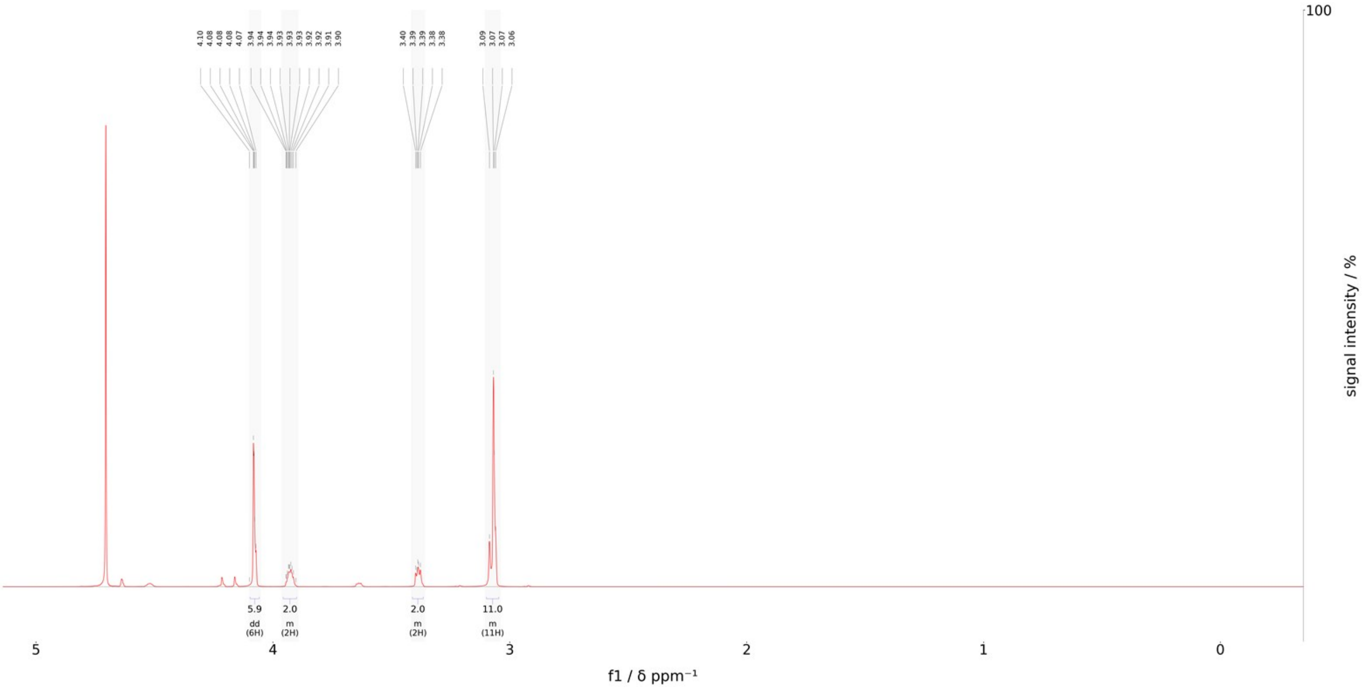

Figure S 21. ^{1}H NMR of ChCl:GA(1:3) in deuterated water.

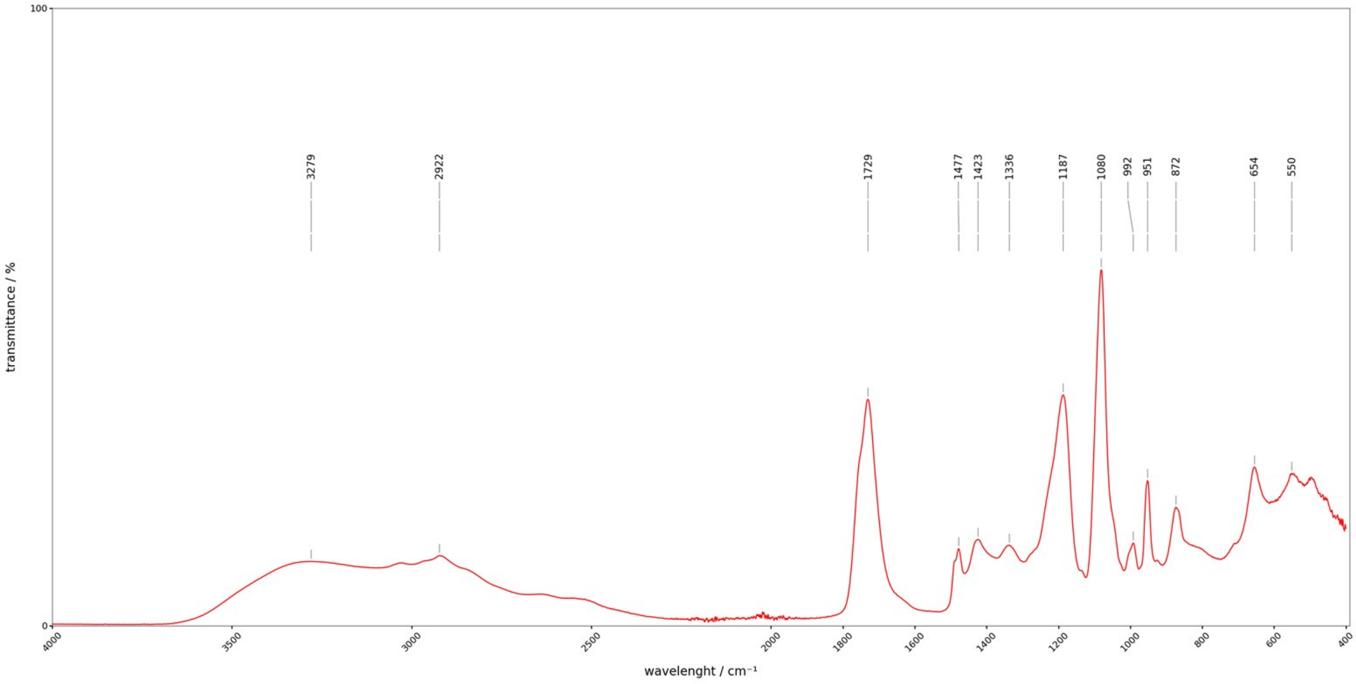

Figure S 22. IR spectra of ChCl:GA(1:3).

Please do not adjust margins

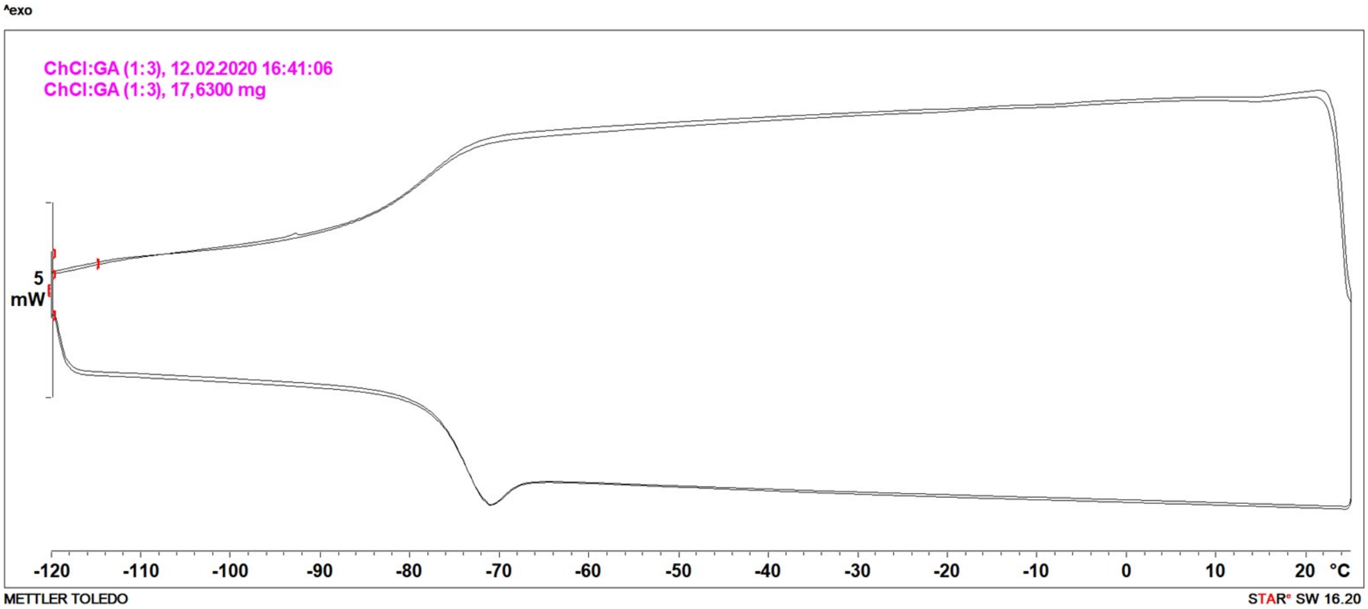

Figure S 23. DSC spectra of ChCl:GA(1:3).

Please do not adjust margins

ChCl:TGA(1:2)

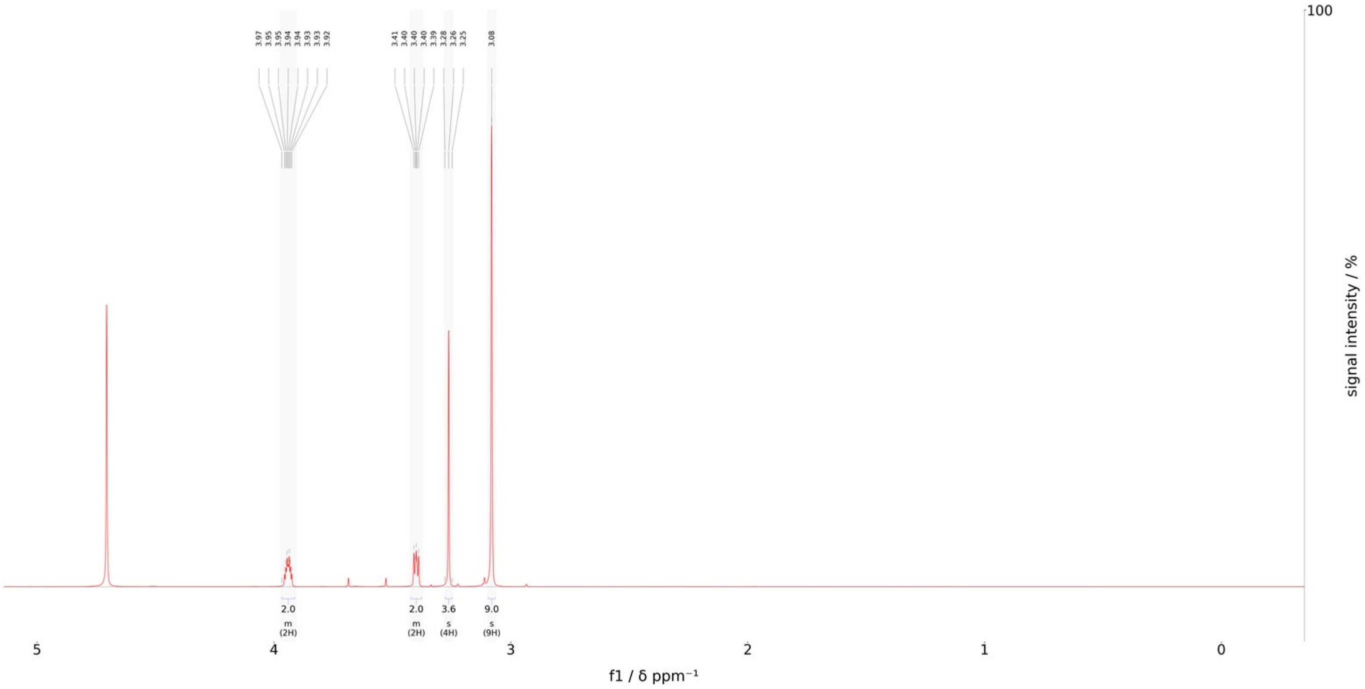

Figure S 24. ^{1}H NMR of ChCl:TGA(1:2) in deuterated water.

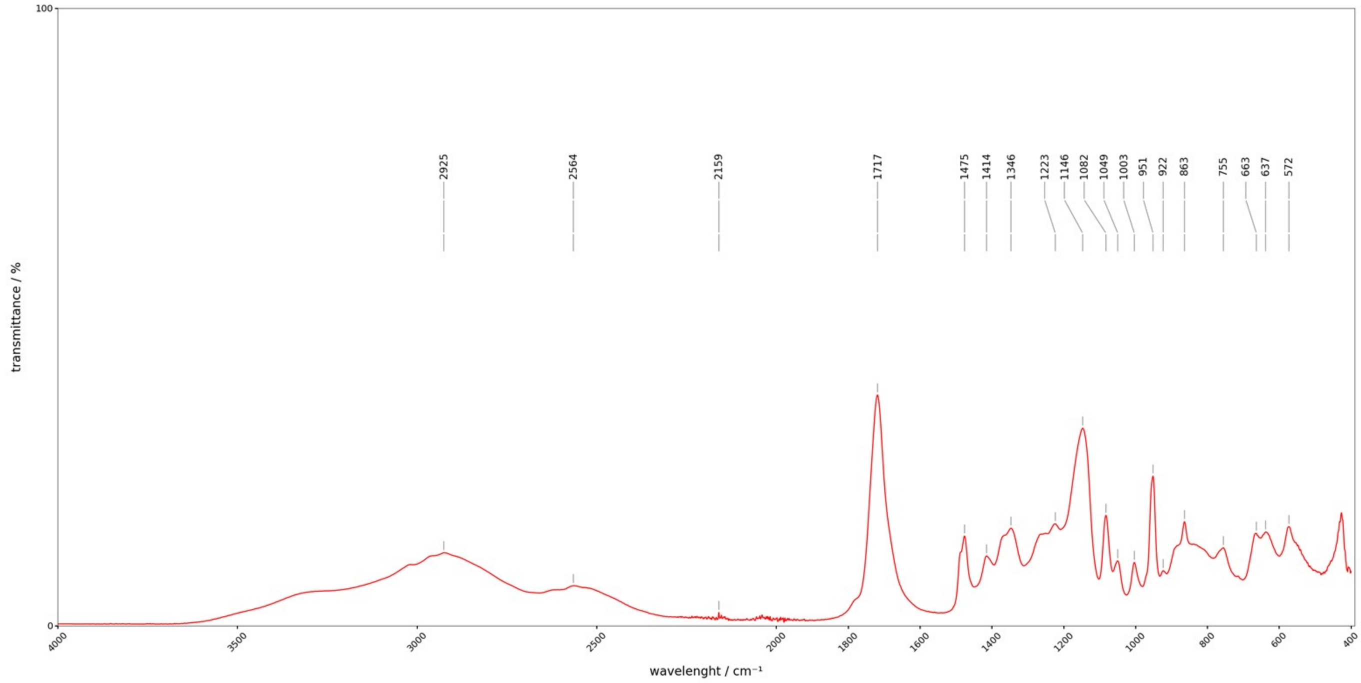

Figure S 25. IR spectra of ChCl:TGA(1:2).

Please do not adjust margins

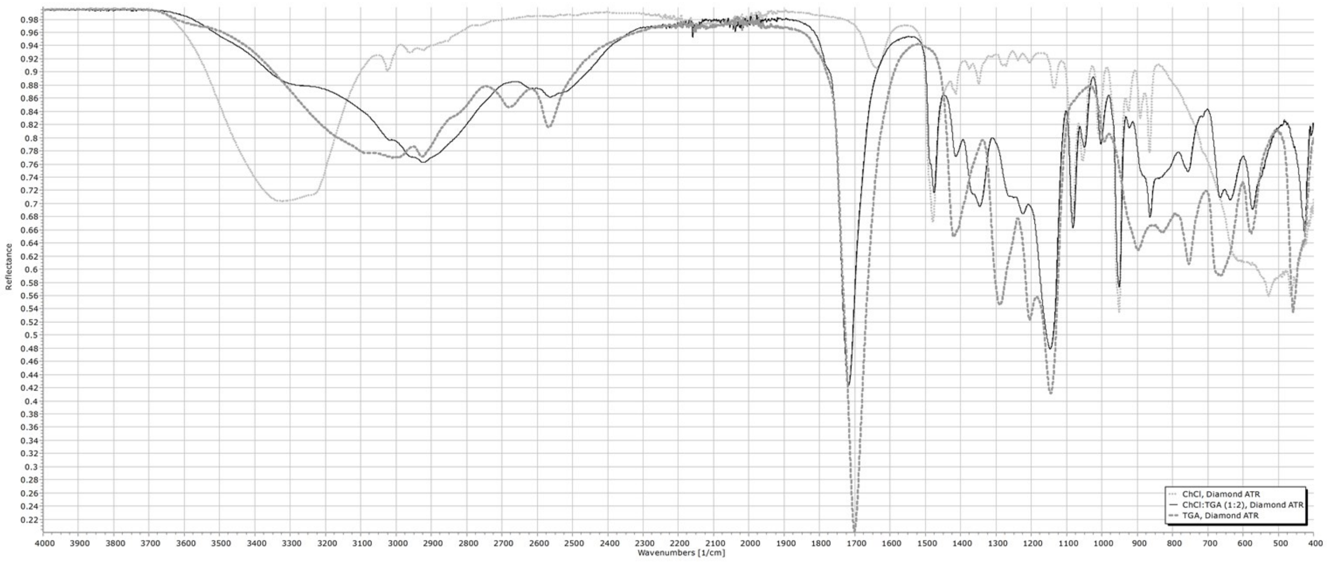

Figure S 26. IR spectra overlap of ChCl:TGA (black solid line), TGA (grey dashed line), and ChCl (light grey dotted line).

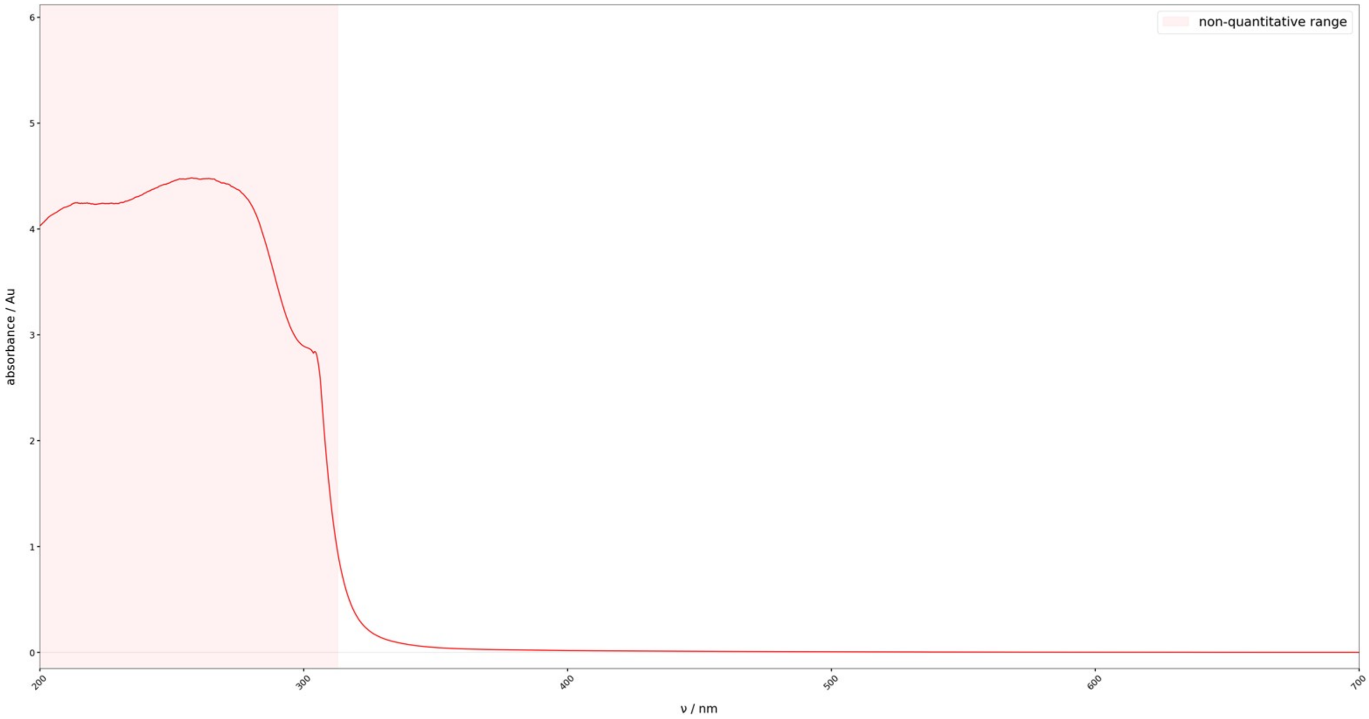

Figure S 27. UV spectra of ChCl:TGA(1:2).

Please do not adjust margins

Please do not adjust margins

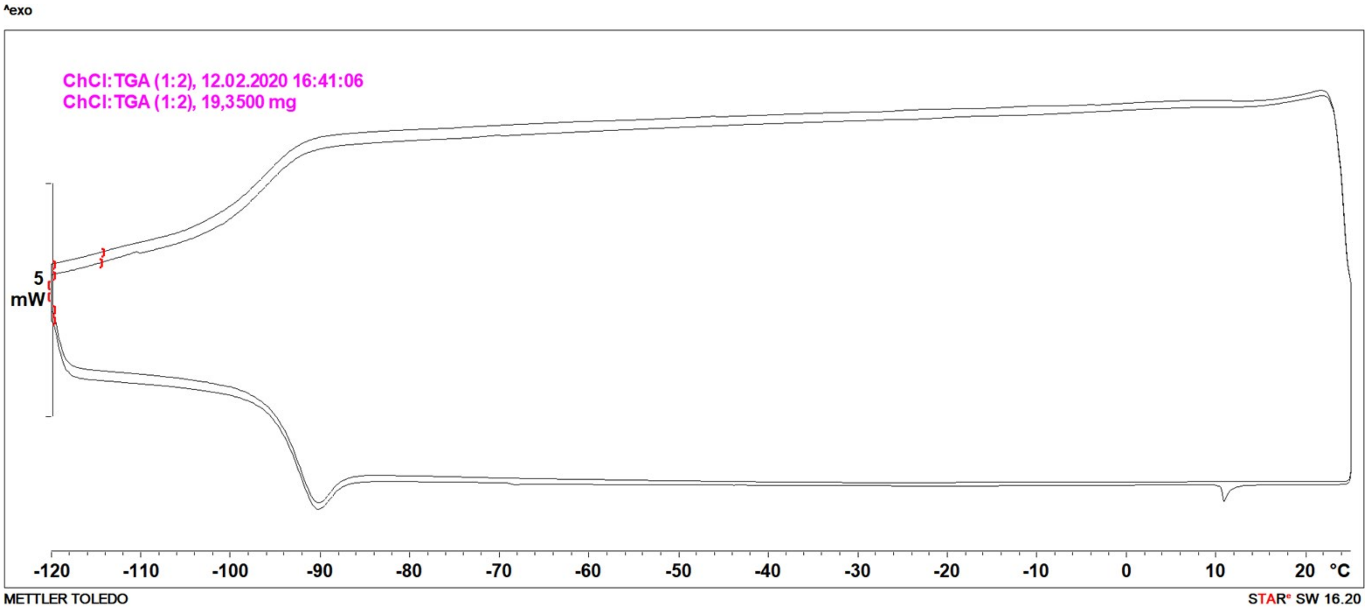

Figure S 28. DSC spectra of ChCl:TGA(1:2).

Please do not adjust margins

Please do not adjust margins

ChCl:TGA(1:3)

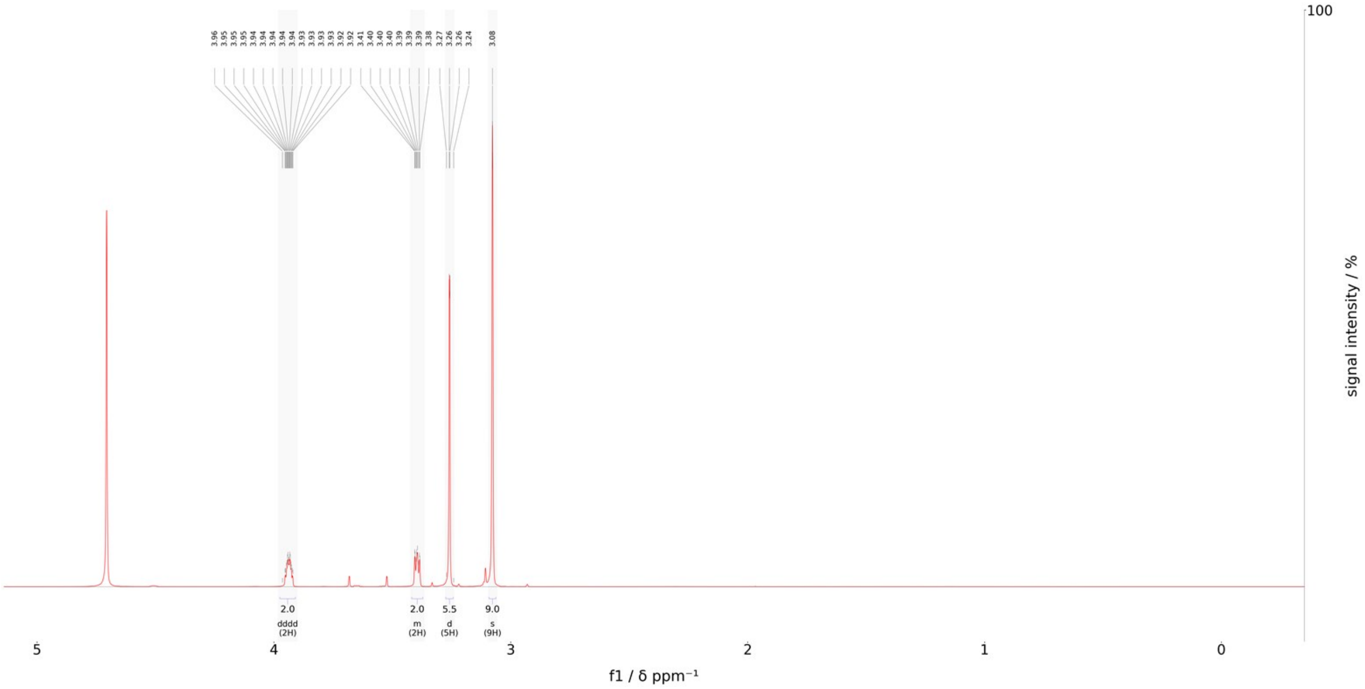

Figure S 29. ^{1}H NMR of ChCl:TGA(1:3) in deuterated water.

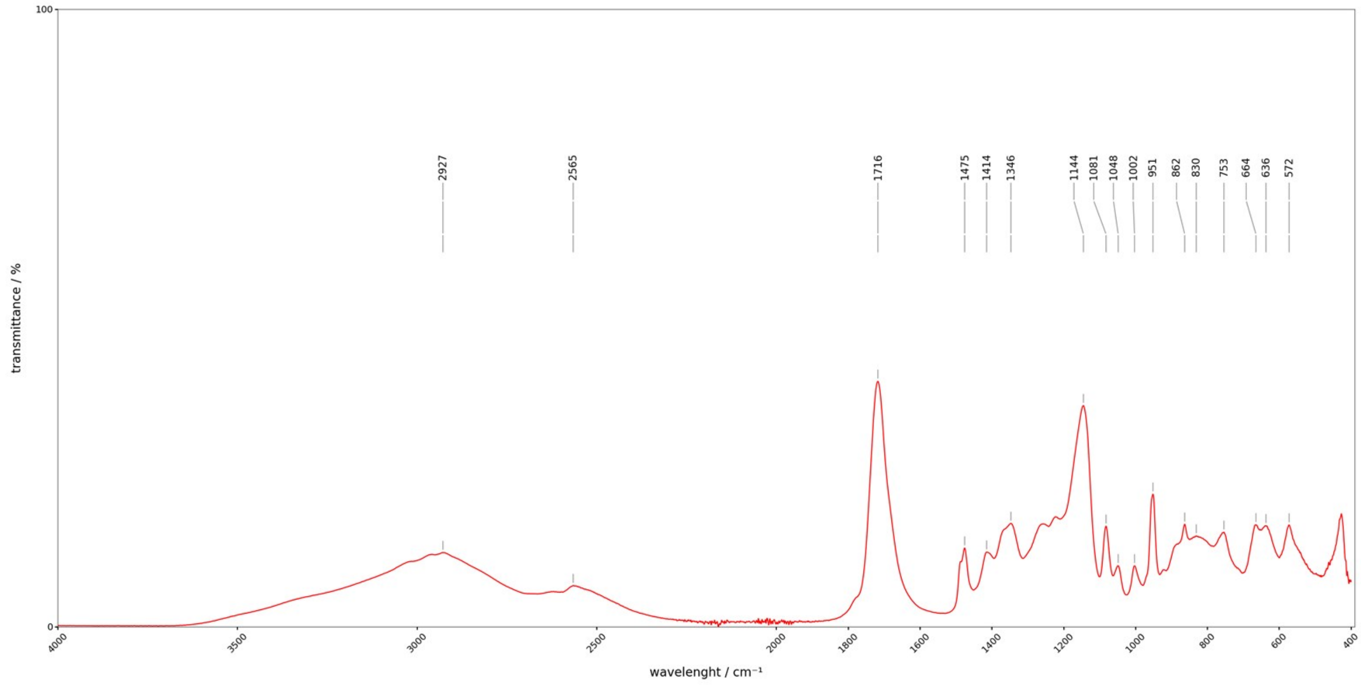

Figure S 30. IR spectra of ChCl:TGA(1:3).

Please do not adjust margins

Please do not adjust margins

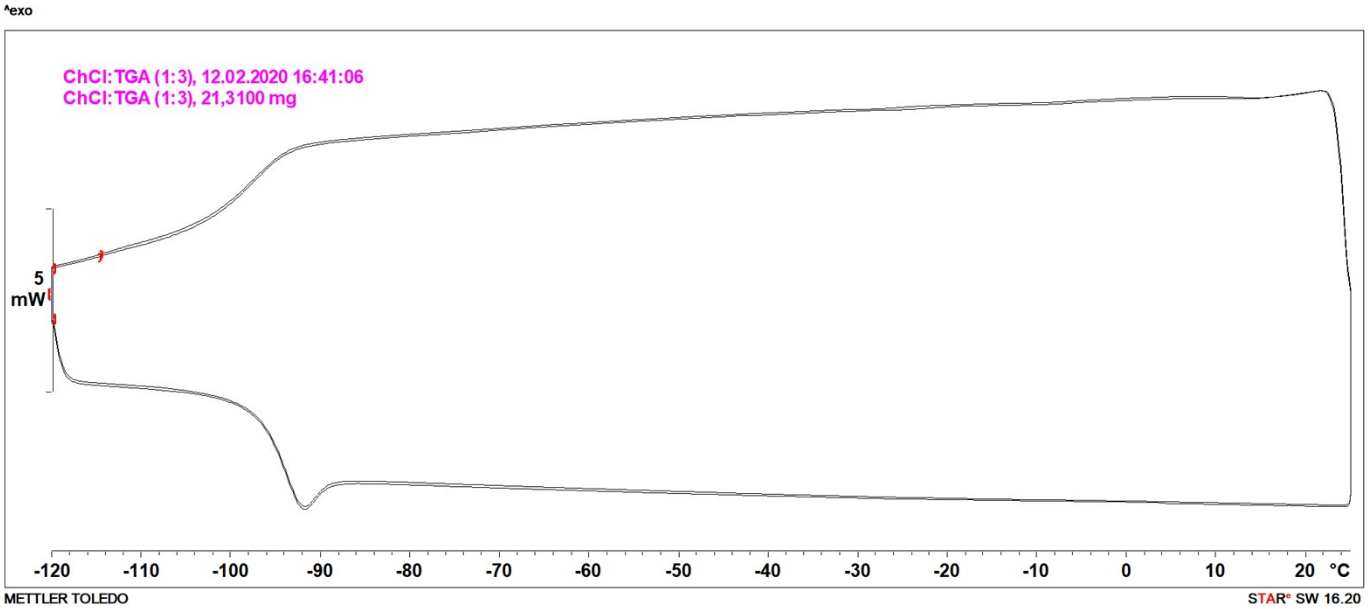

Figure S 31. DSC spectra of ChCl:TGA(1:3).

Please do not adjust margins

Please do not adjust margins

ChCl:LA(1:2)

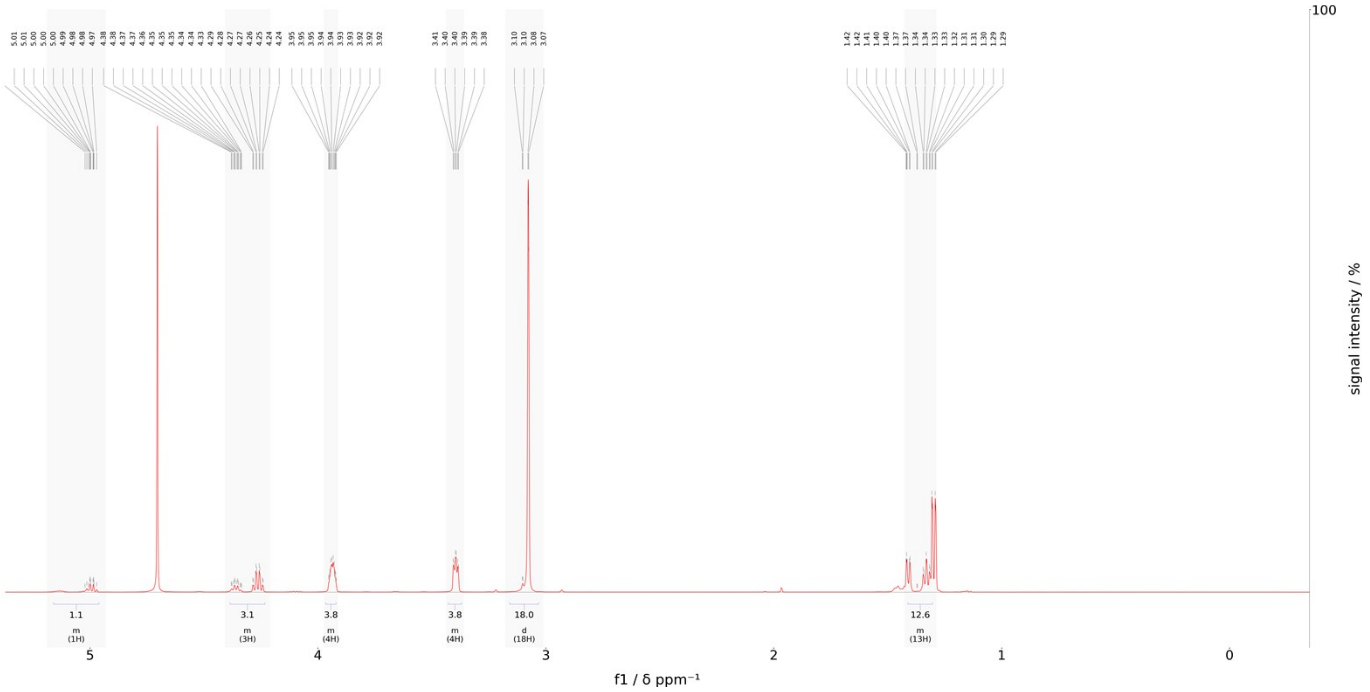

Figure S 32. ^{1}H NMR of ChCl:LA(1:2) in deuterated water.

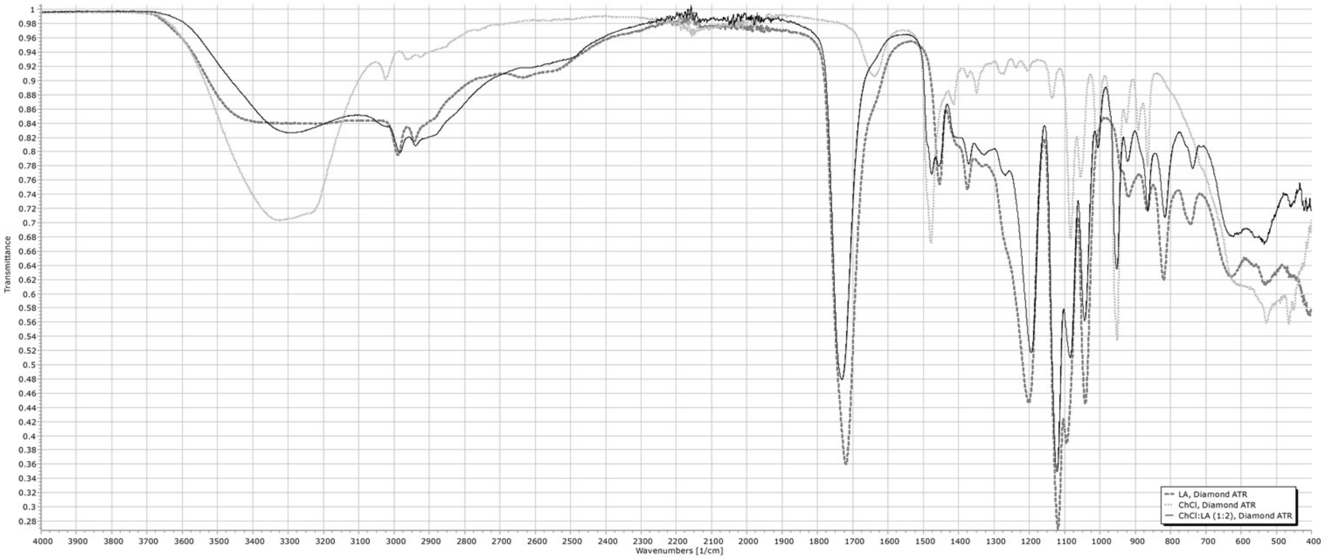

Figure S 33. IR spectra overlap of ChCl:LA (black solid line), LA (grey dashed line), and ChCl (light grey dotted line).

Please do not adjust margins

Please do not adjust margins

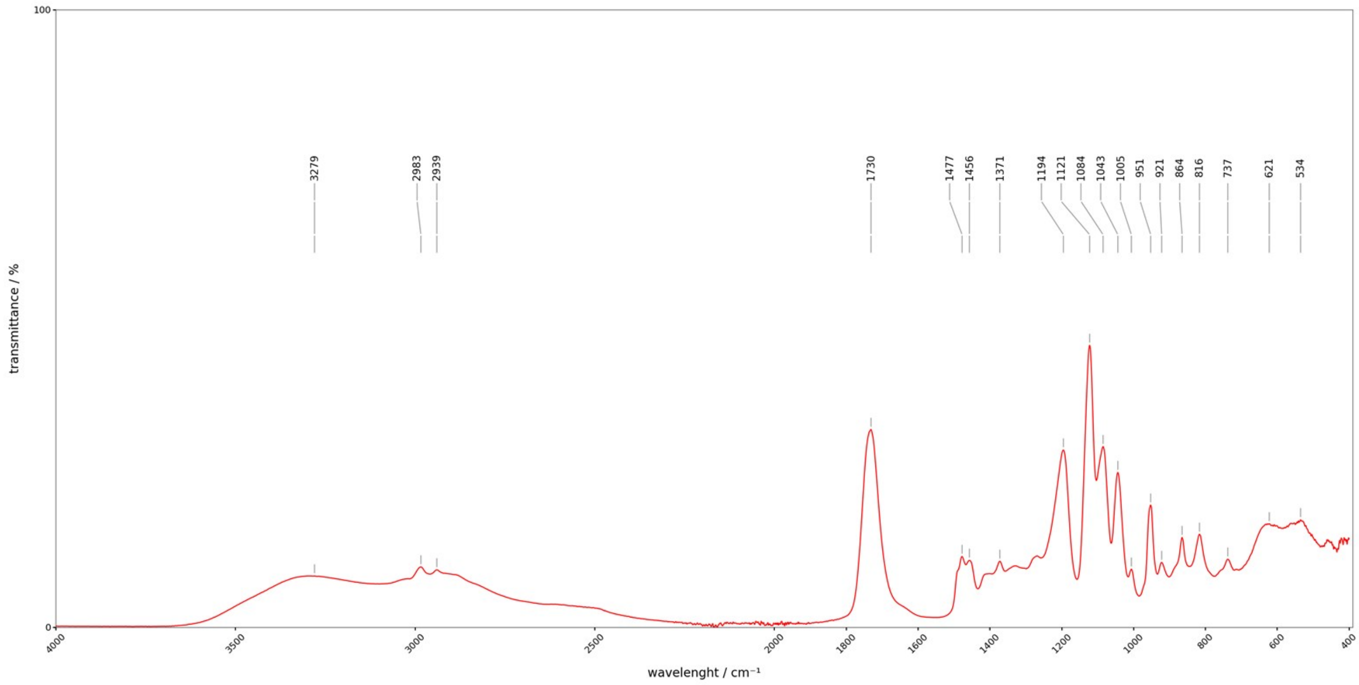

Figure S 34. IR spectra of ChCl:LA(1:2).

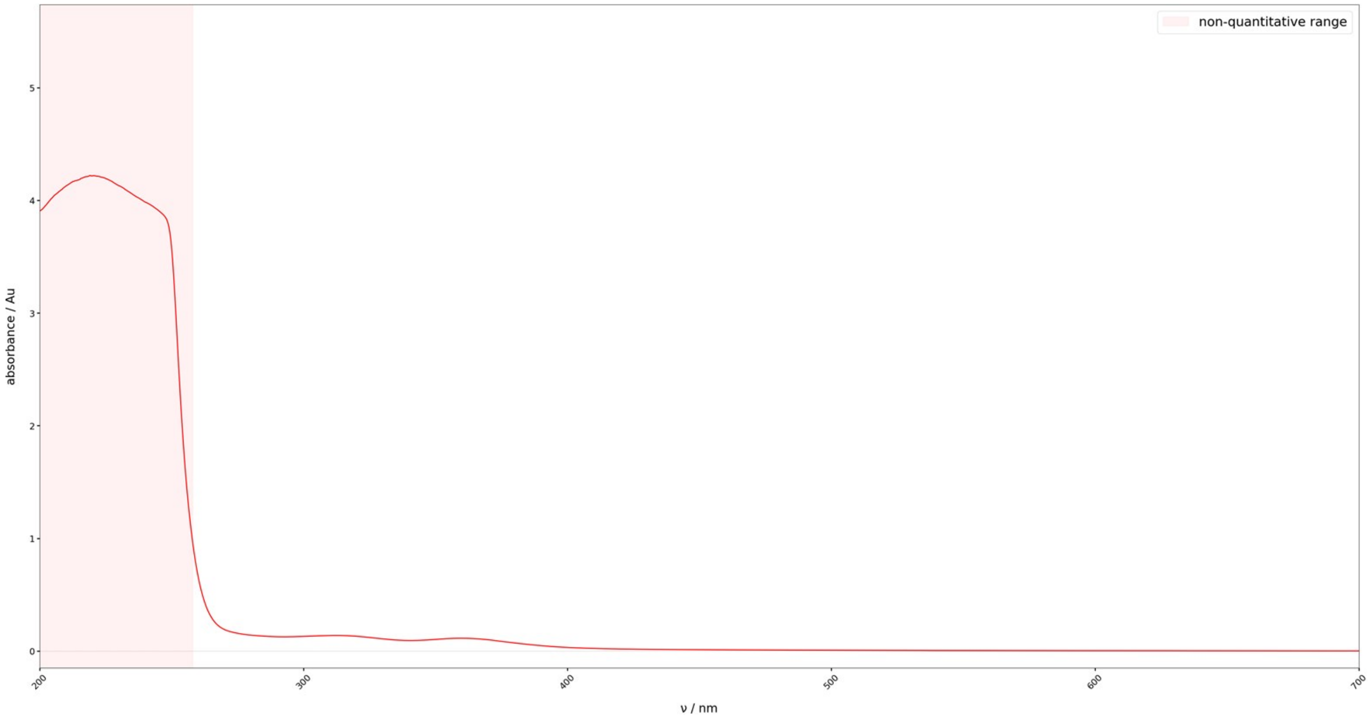

Figure S 35. UV spectra of ChCl:LA(1:2).

Please do not adjust margins

Please do not adjust margins

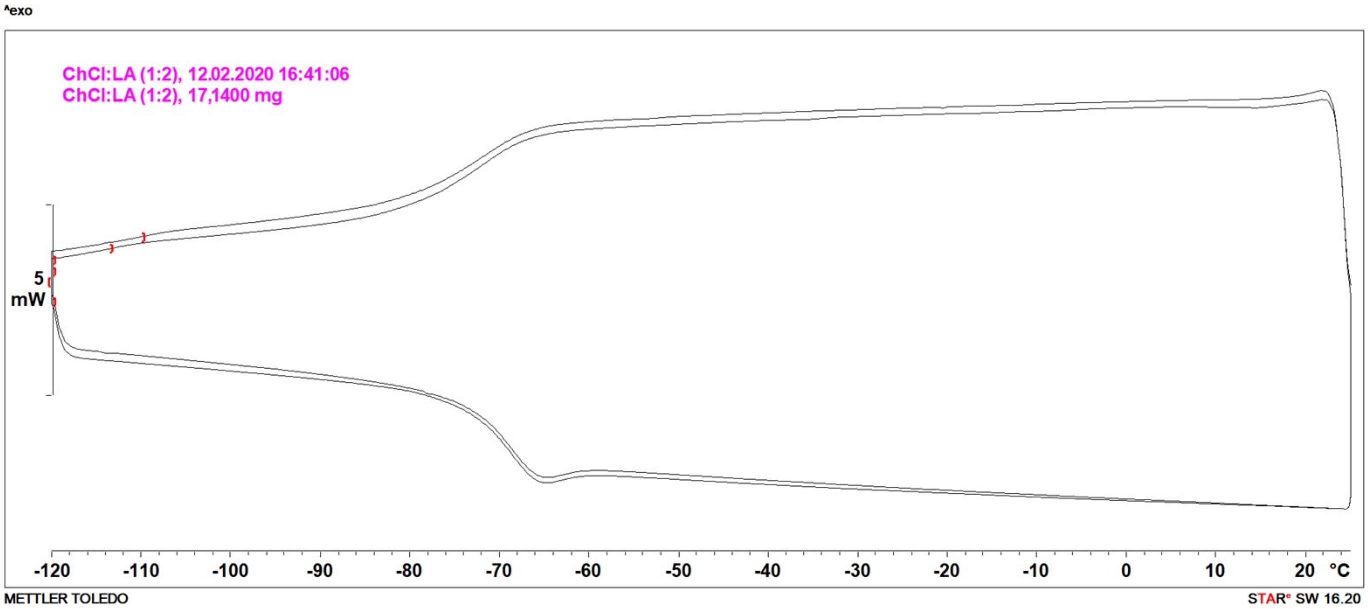

Figure S 36. DSC spectra of ChCl:LA(1:2).

Please do not adjust margins

Please do not adjust margins

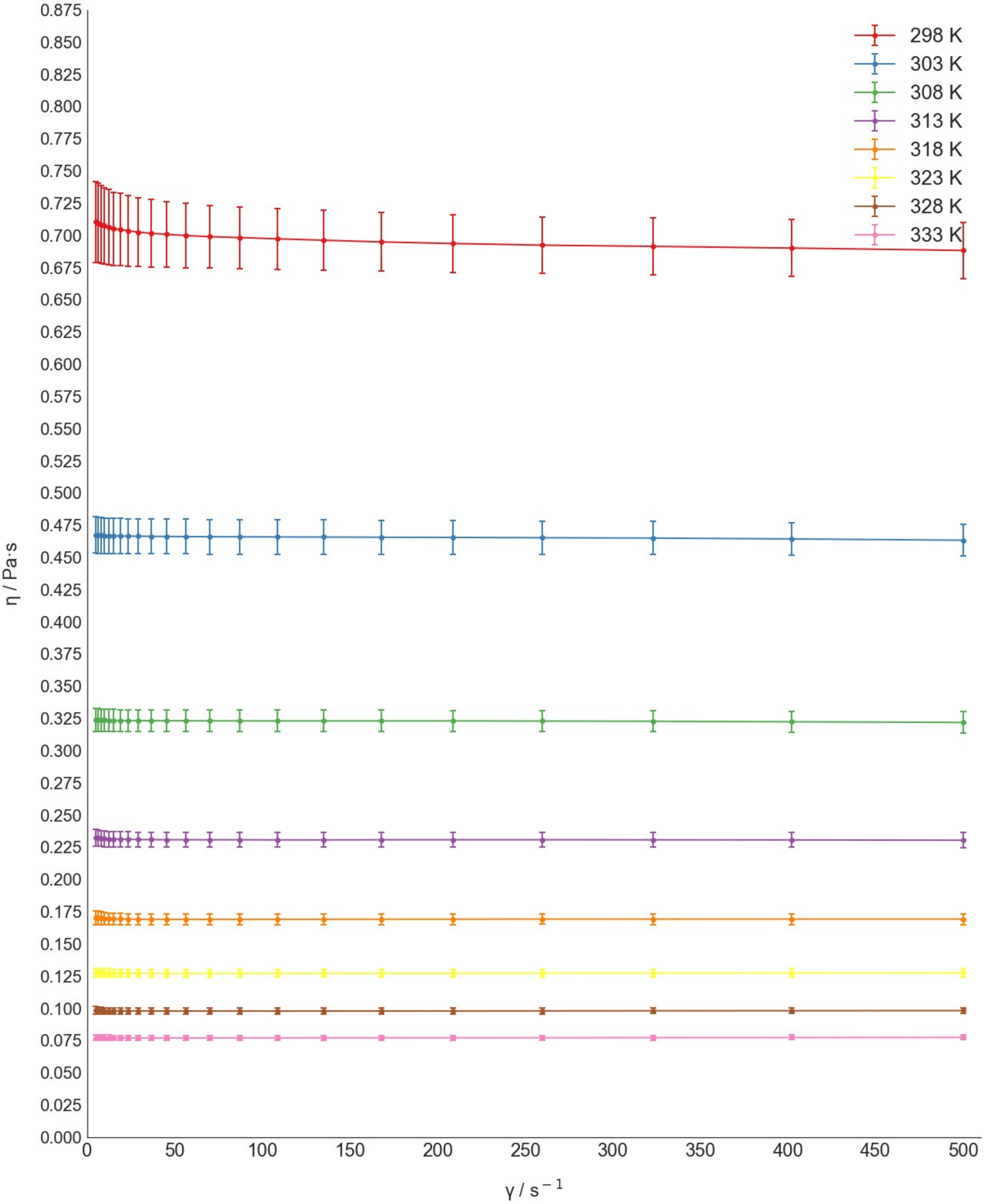

Figure S 37. Viscosity measurements for ChCl:LA(1:2).

Please do not adjust margins

Please do not adjust margins

ChCl:LA(1:3)

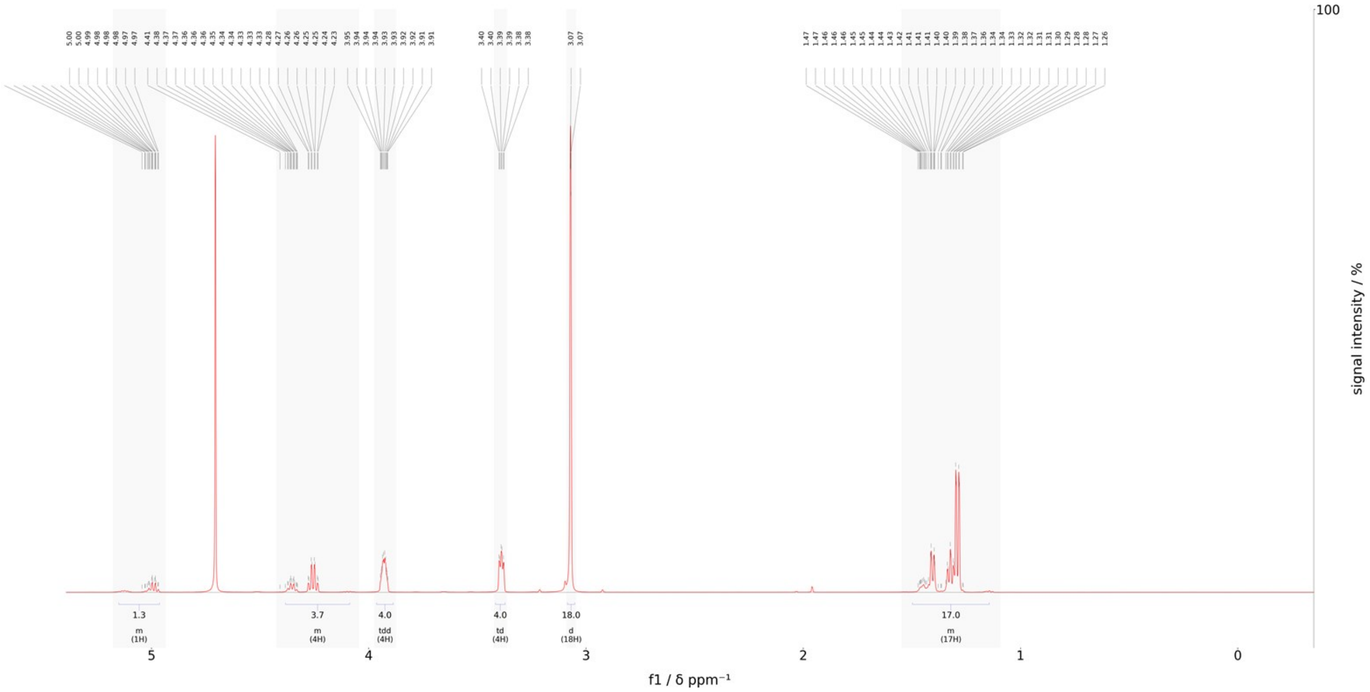

Figure S 38. ^{1}H NMR of ChCl:LA(1:3) in deuterated water.

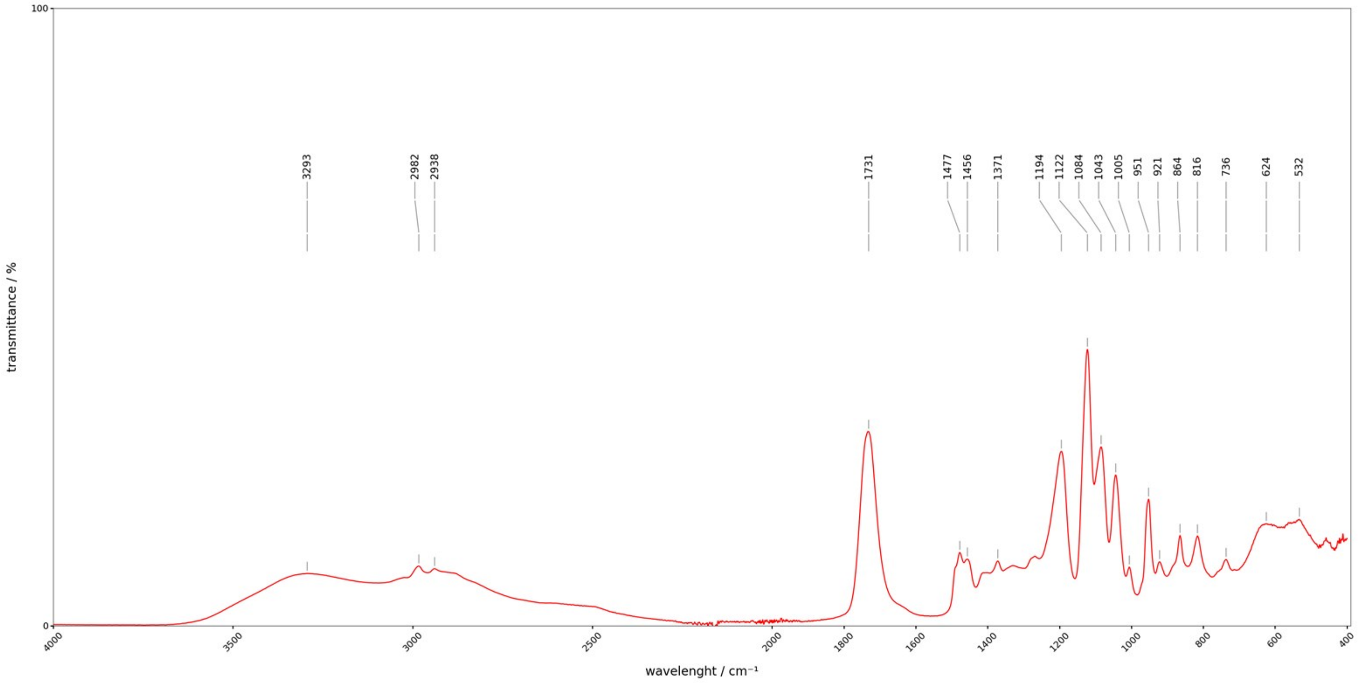

Figure S 39. IR spectra of ChCl:LA(1:3).

Please do not adjust margins

Please do not adjust margins

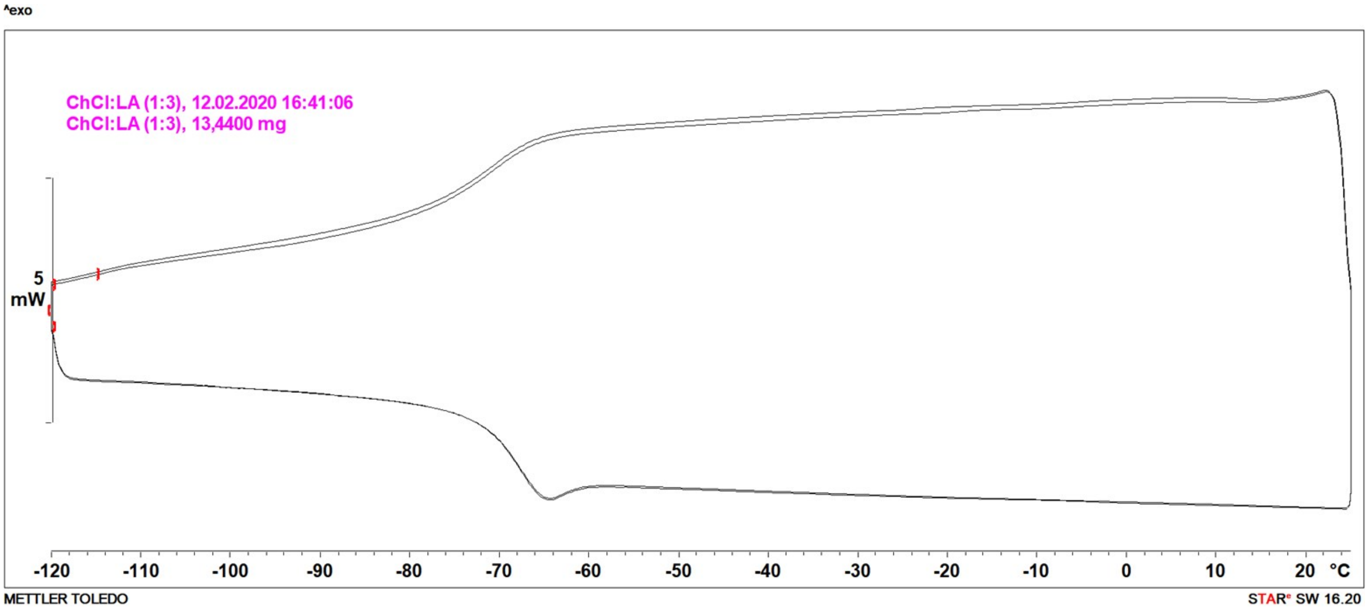

Figure S 40. DSC spectra of ChCl:LA(1:3).

Please do not adjust margins

ChCl:TLA(1:2)

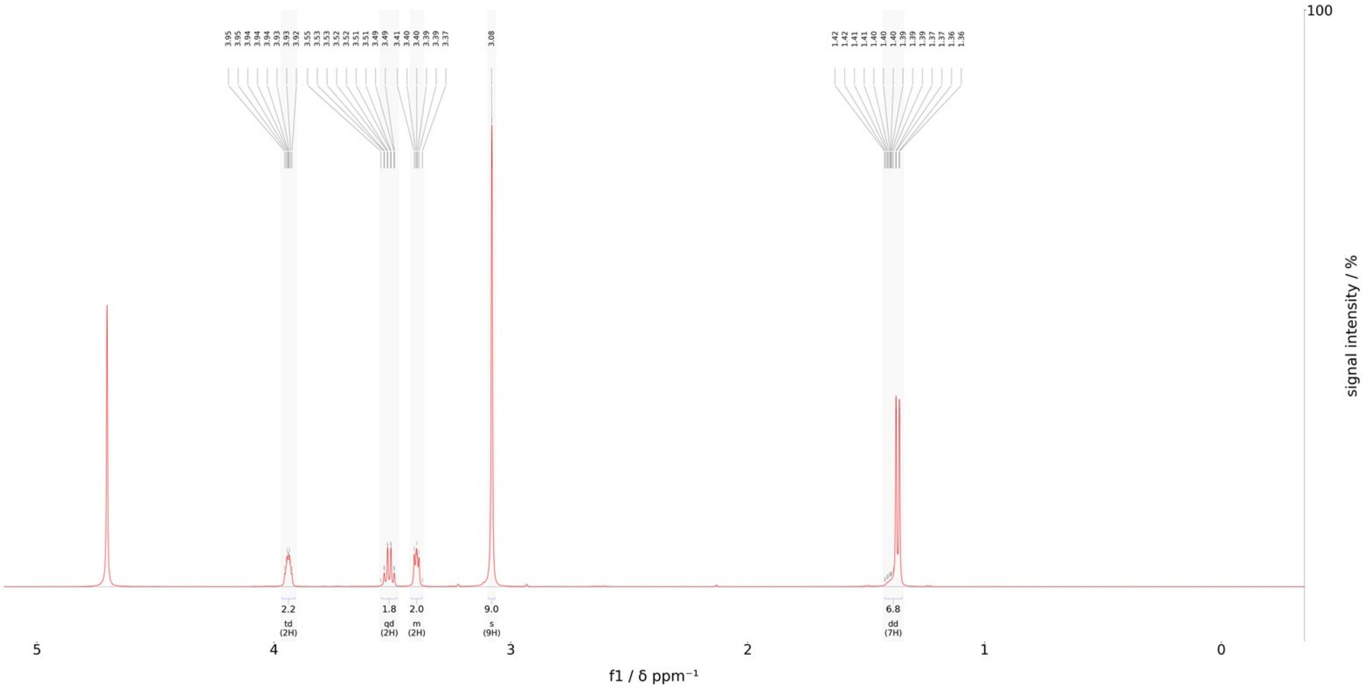

Figure S 41. ^{1}H NMR of ChCl:TLA(1:2) in deuterated water.

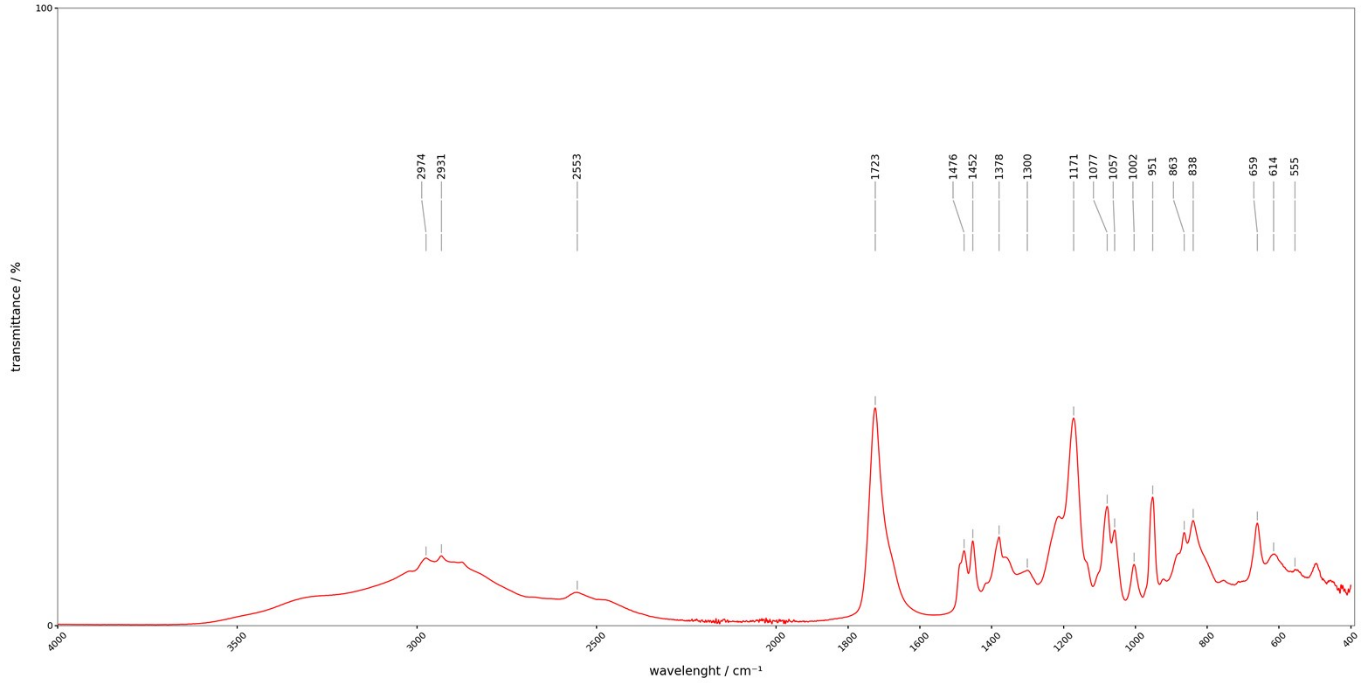

Figure S 42. IR spectra of ChCl:TLA(1:2).

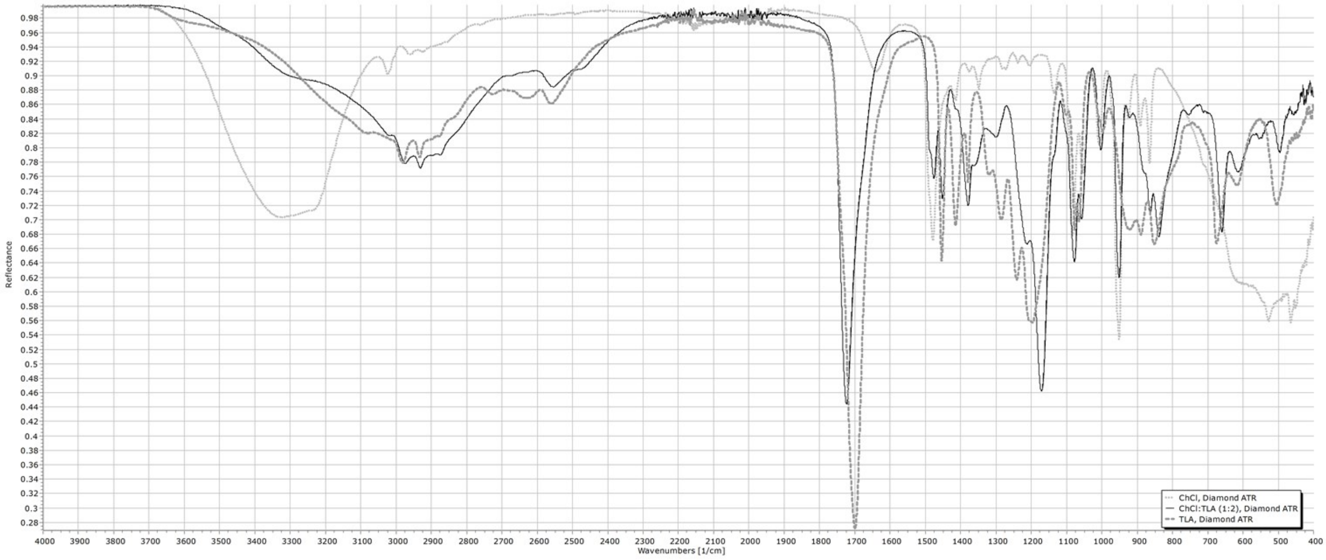

Figure S 43. IR spectra overlap of ChCl:TLA (black solid line), TLA (grey dashed line), and ChCl (light grey dotted line).

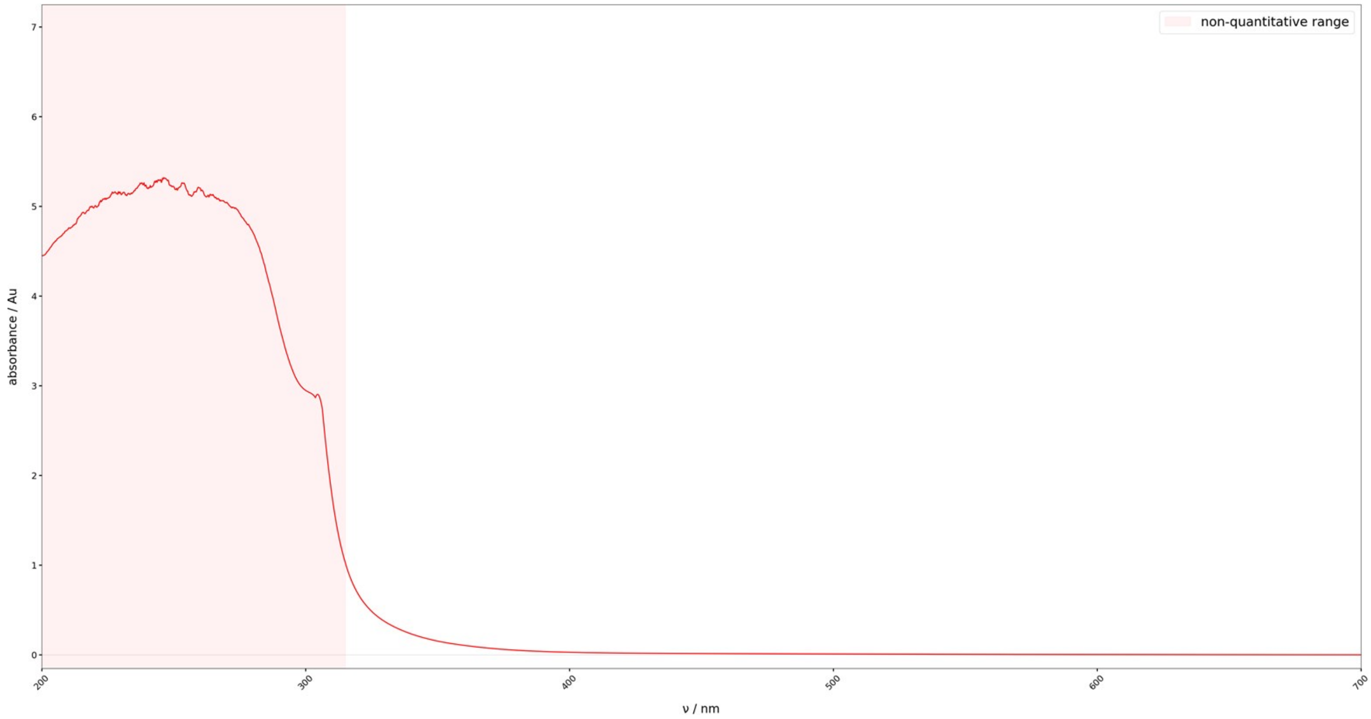

Figure S 44. UV spectra of ChCl:TLA(1:2).

Please do not adjust margins

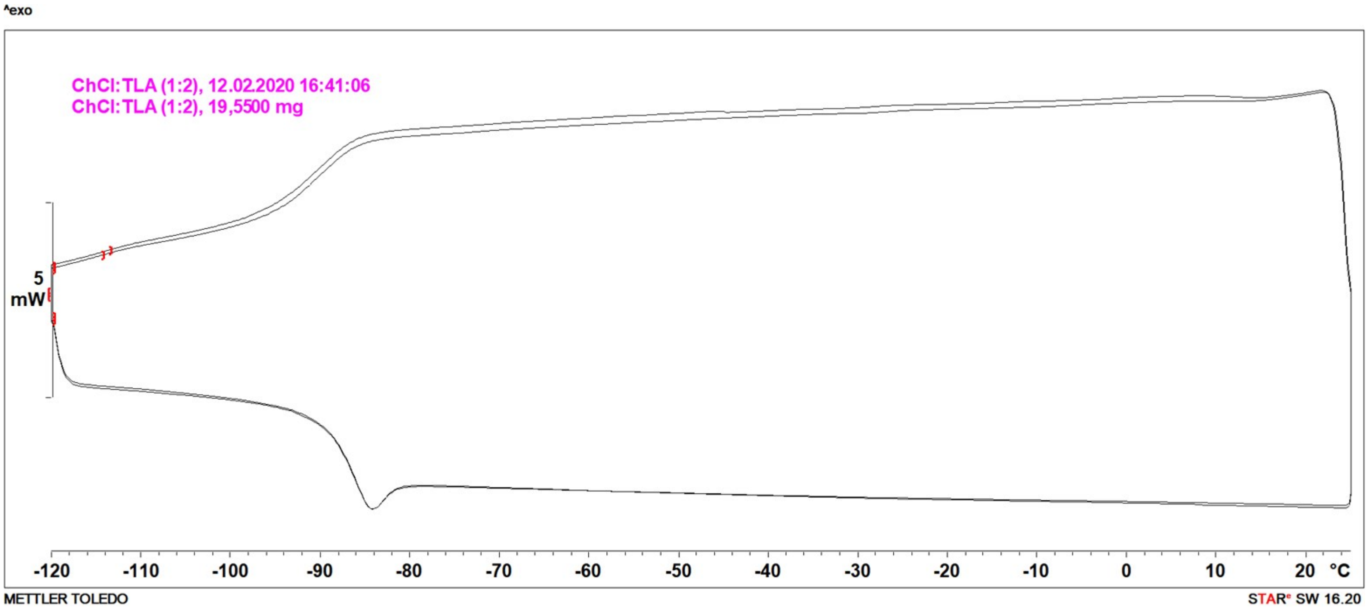

Figure S 45. DSC spectra of ChCl:TLA(1:2).

Please do not adjust margins

Please do not adjust margins

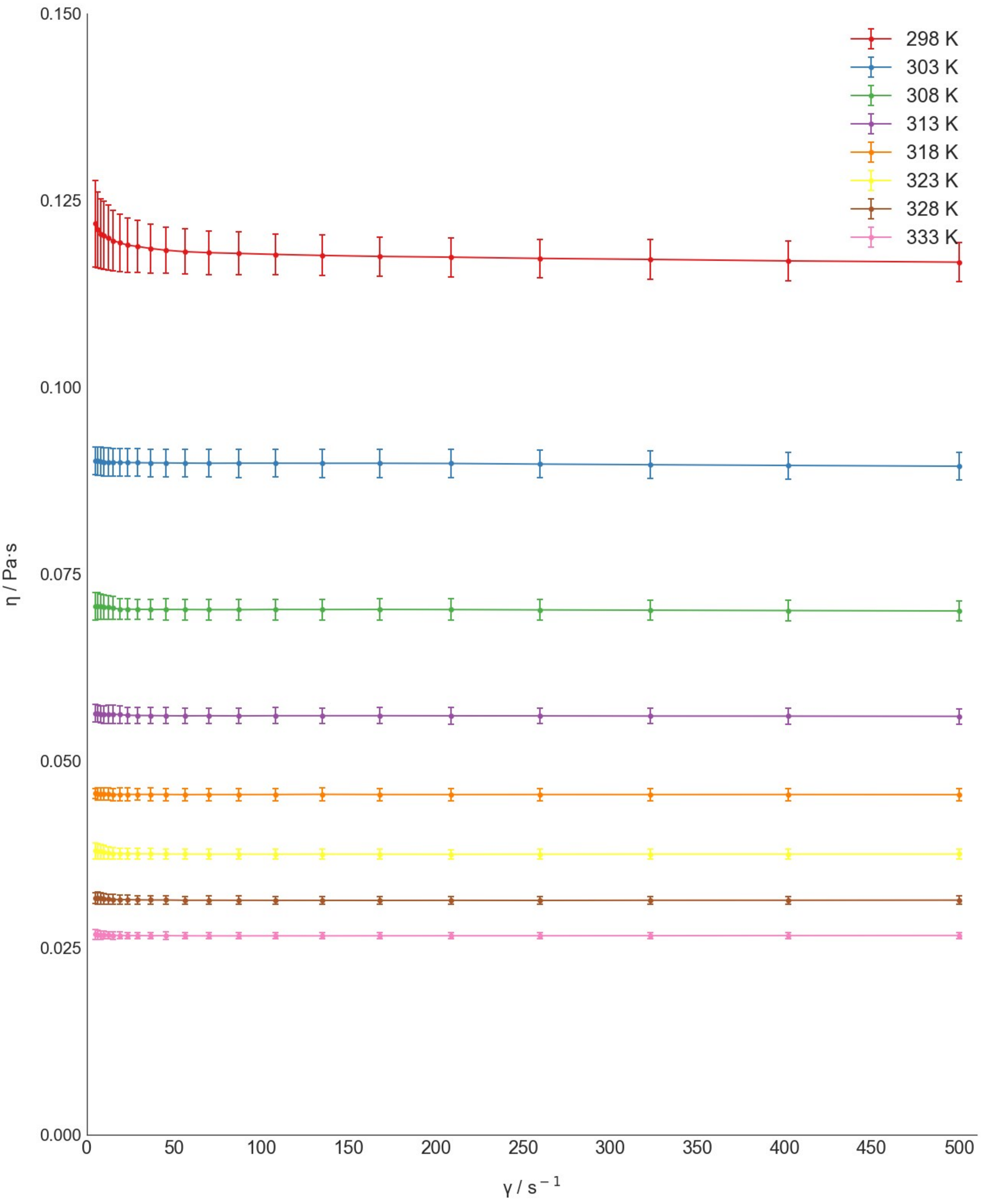

Figure S 46. Viscosity measurements for ChCl:TLA(1:2).

Please do not adjust margins

ChCl:TLA(1:3)

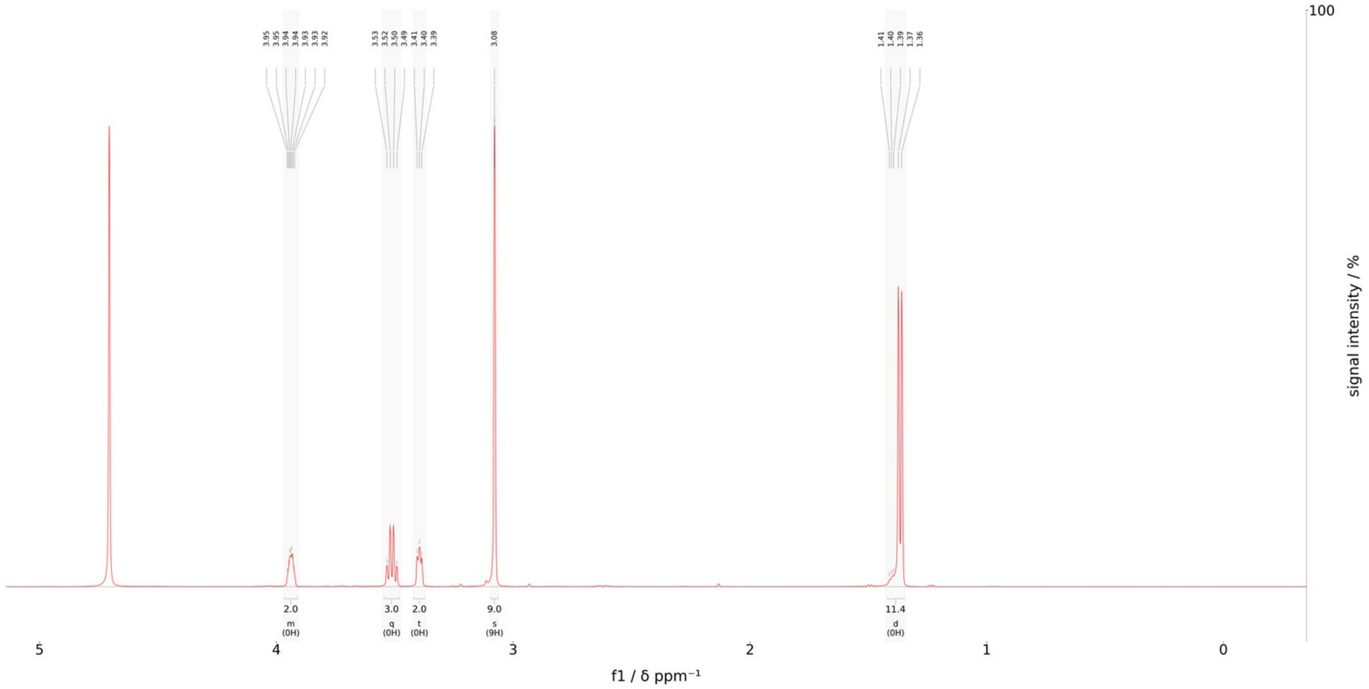

Figure S 47. ¹H NMR of ChCl:TLA(1:3) in deuterated water.

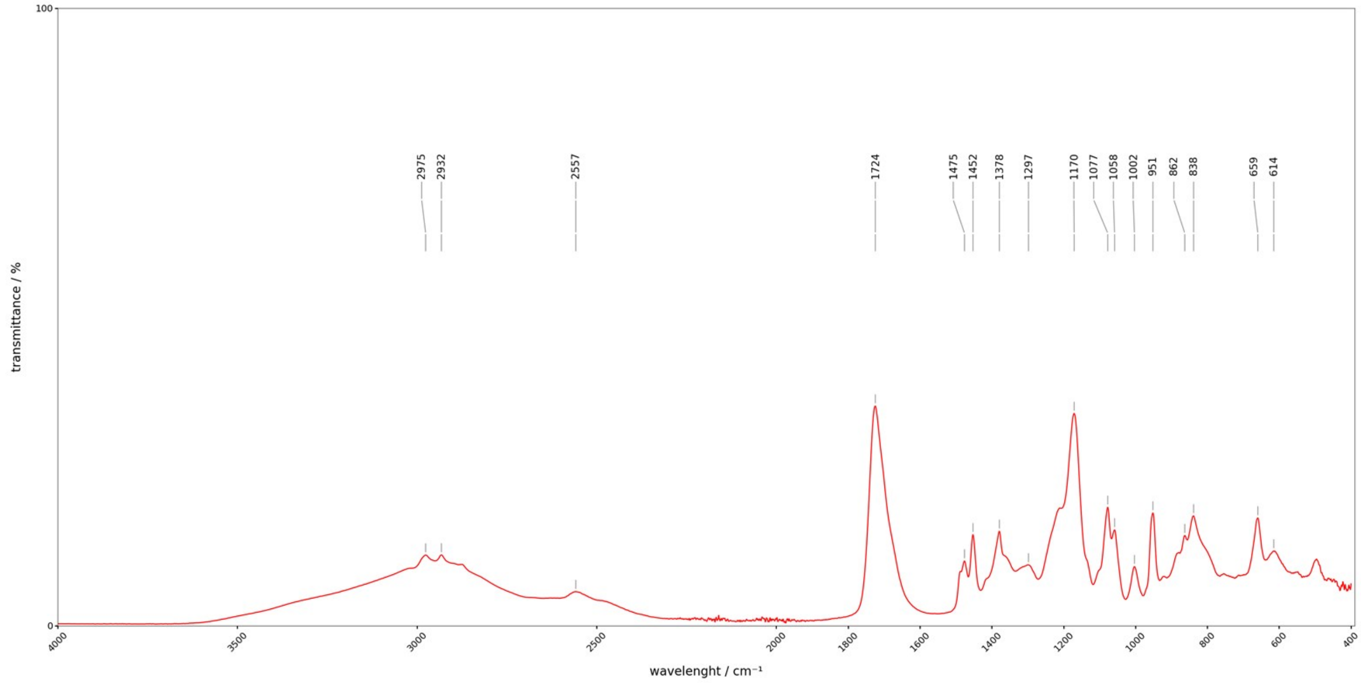

Figure S 48. IR spectra of ChCl:TLA(1:3).

Please do not adjust margins

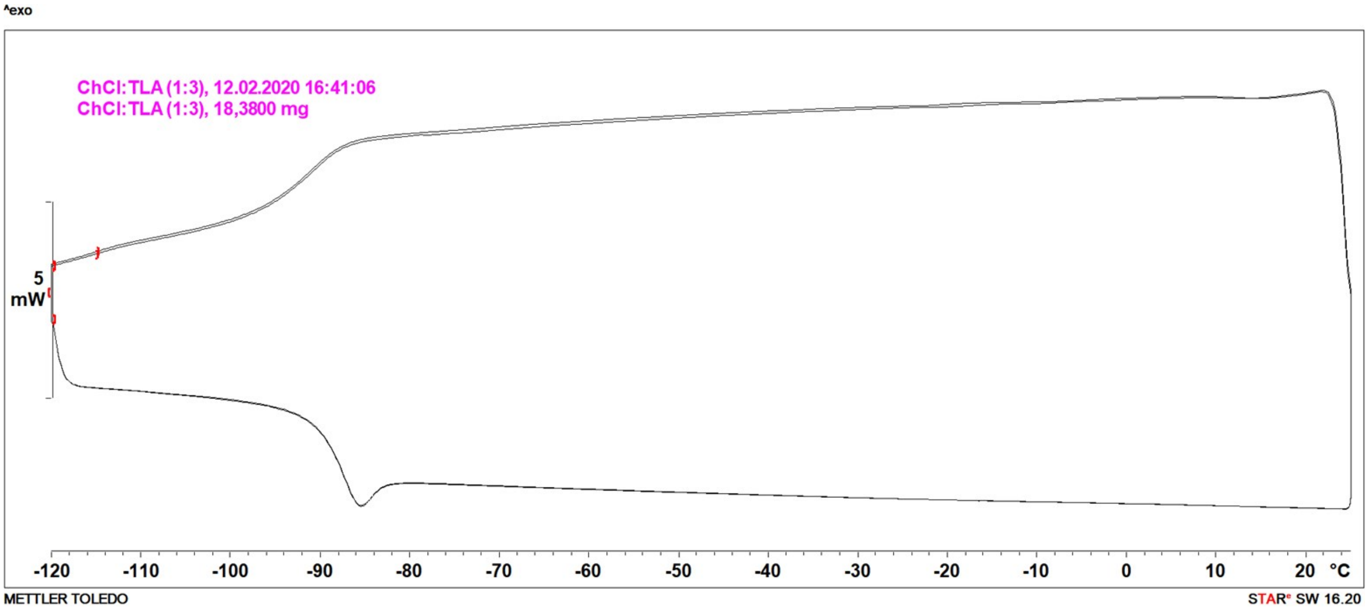

Figure S 49. DSC spectra of ChCl:TLA(1:3).

Please do not adjust margins

ChCl:MA(1:1)

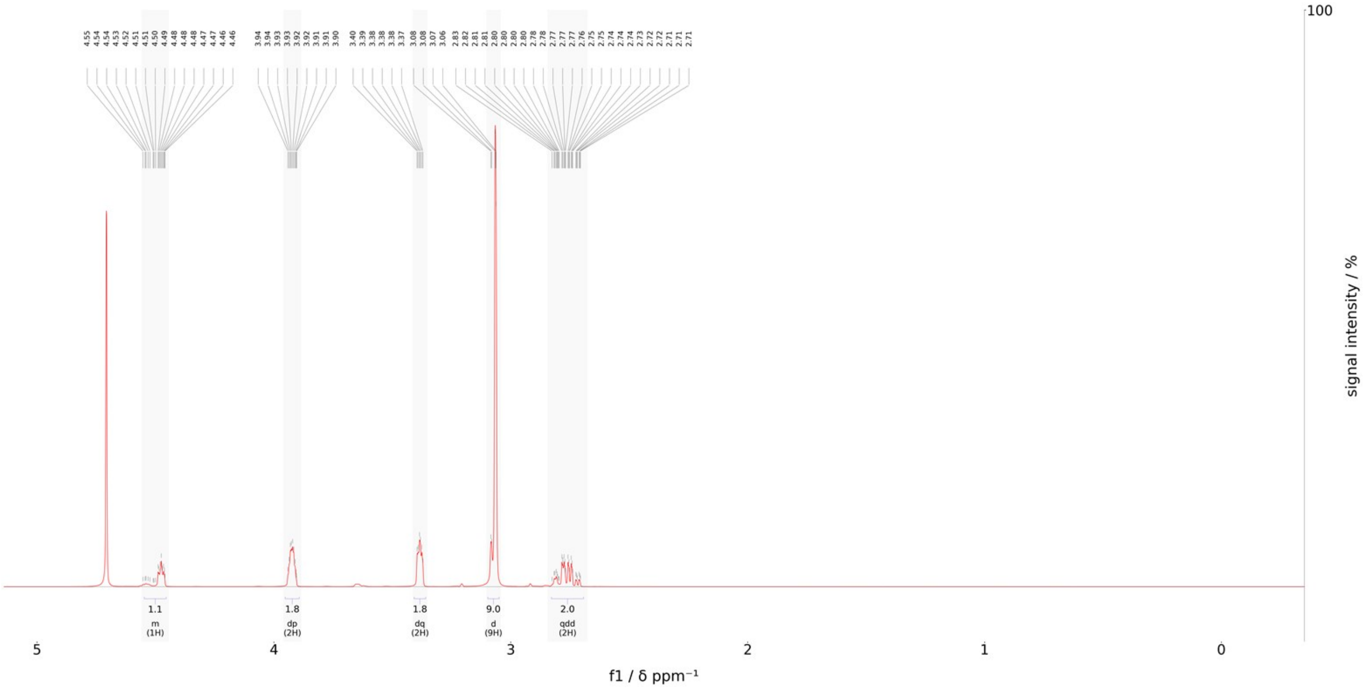

Figure S 50. ^{1}H NMR of ChCl:MA(1:1) in deuterated water.

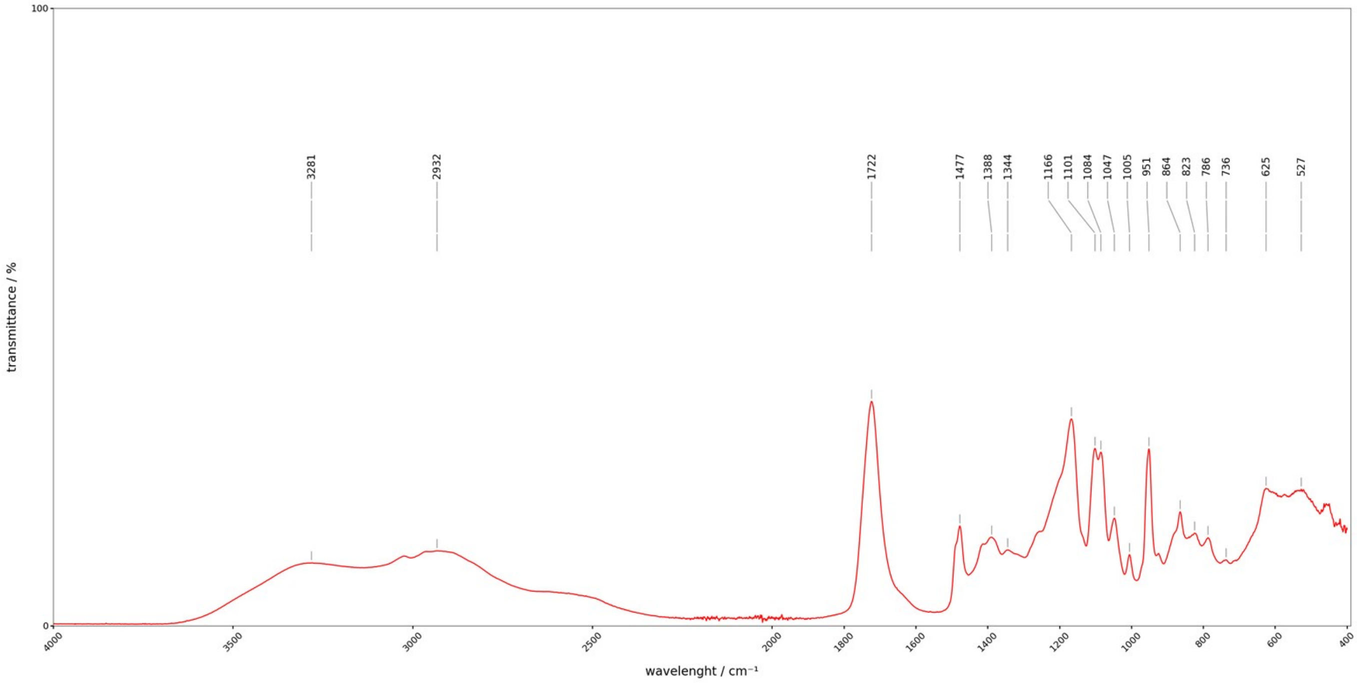

Figure S 51. IR spectra of ChCl:MA(1:1).

Please do not adjust margins
Please do not adjust margins

Please do not adjust margins

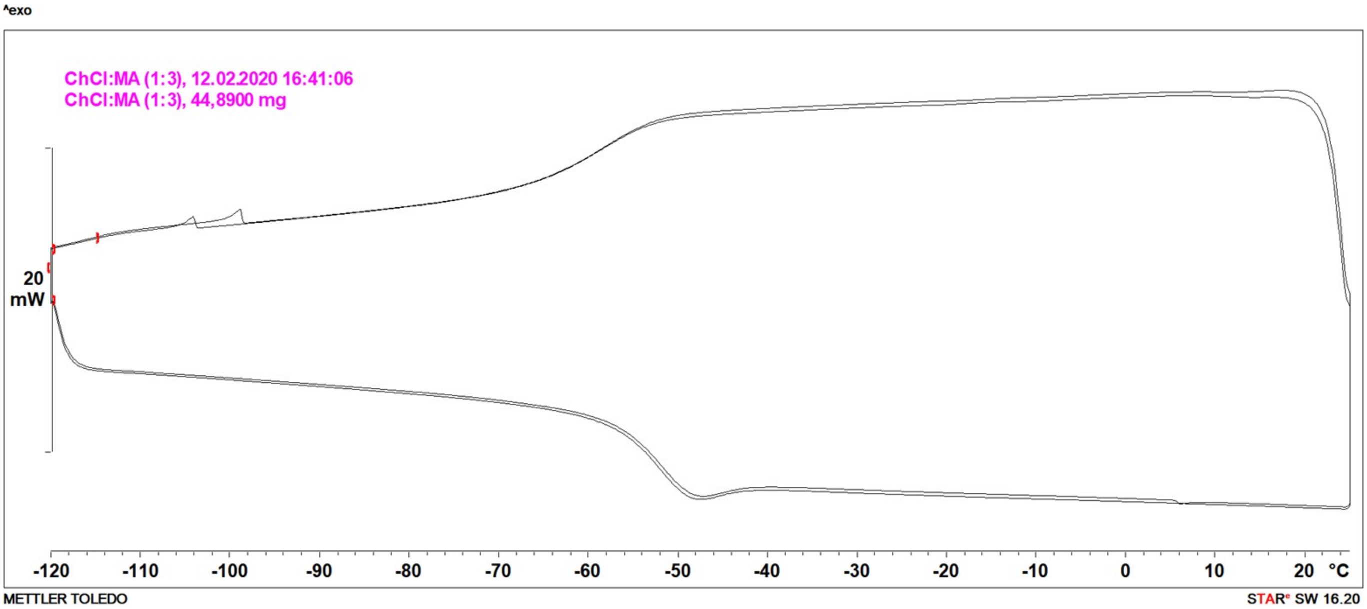

Figure S 52. DSC spectra of ChCl:MA(1:1).

Please do not adjust margins

Please do not adjust margins

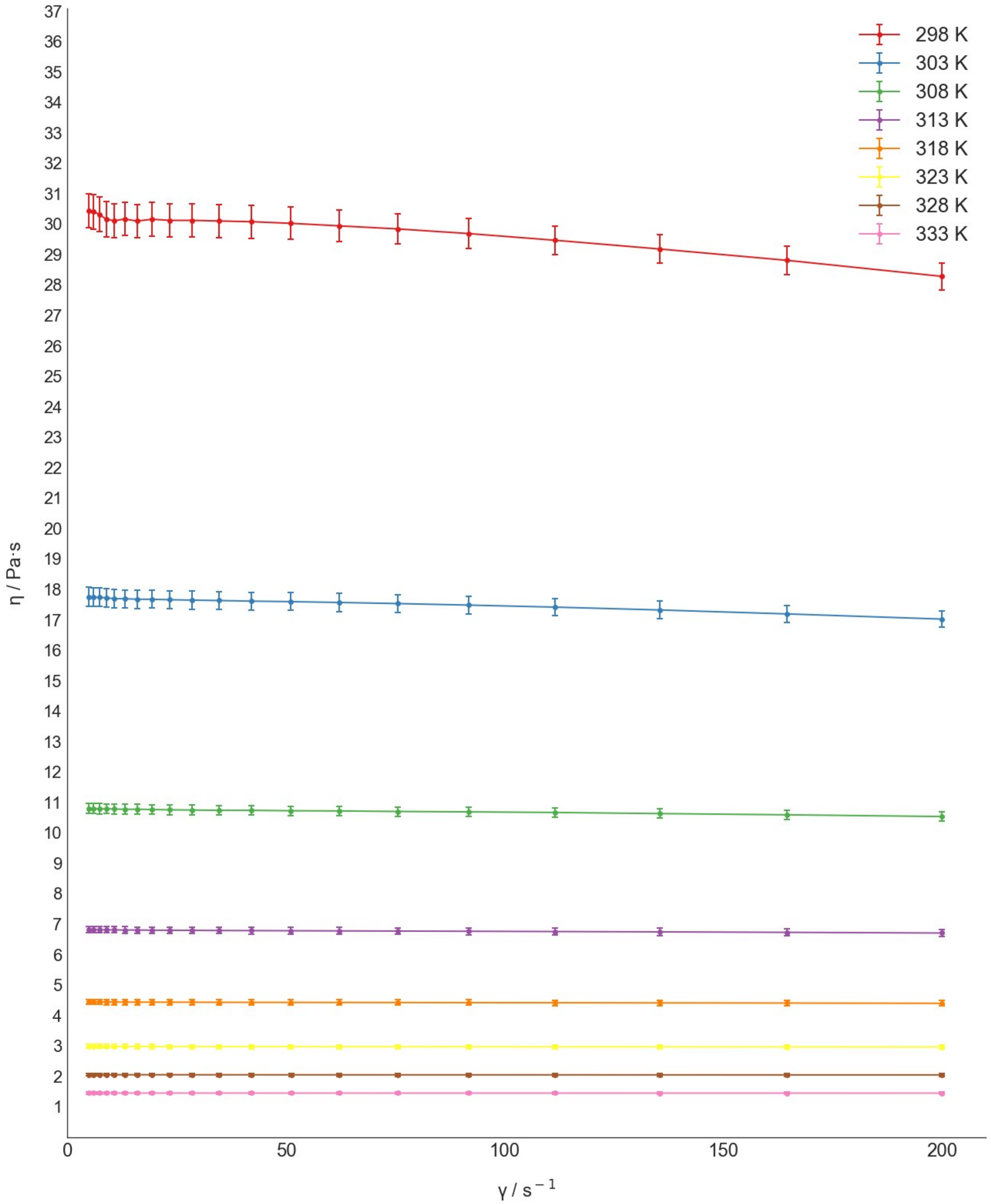

Figure S 53. Viscosity measurements for ChCl:MA(1:1).

Please do not adjust margins

ChCl:TMA(1:1)

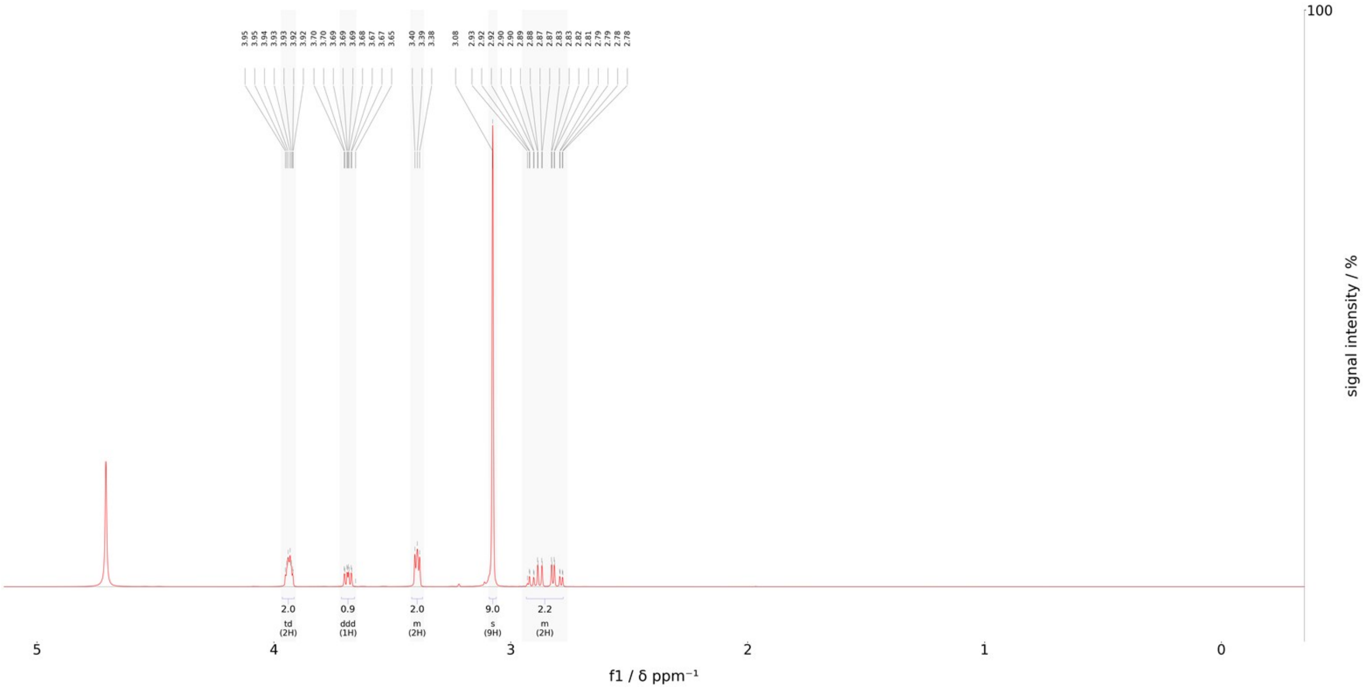

Figure S 54. ^{1}H NMR of ChCl:TMA(1:1) in deuterated water.

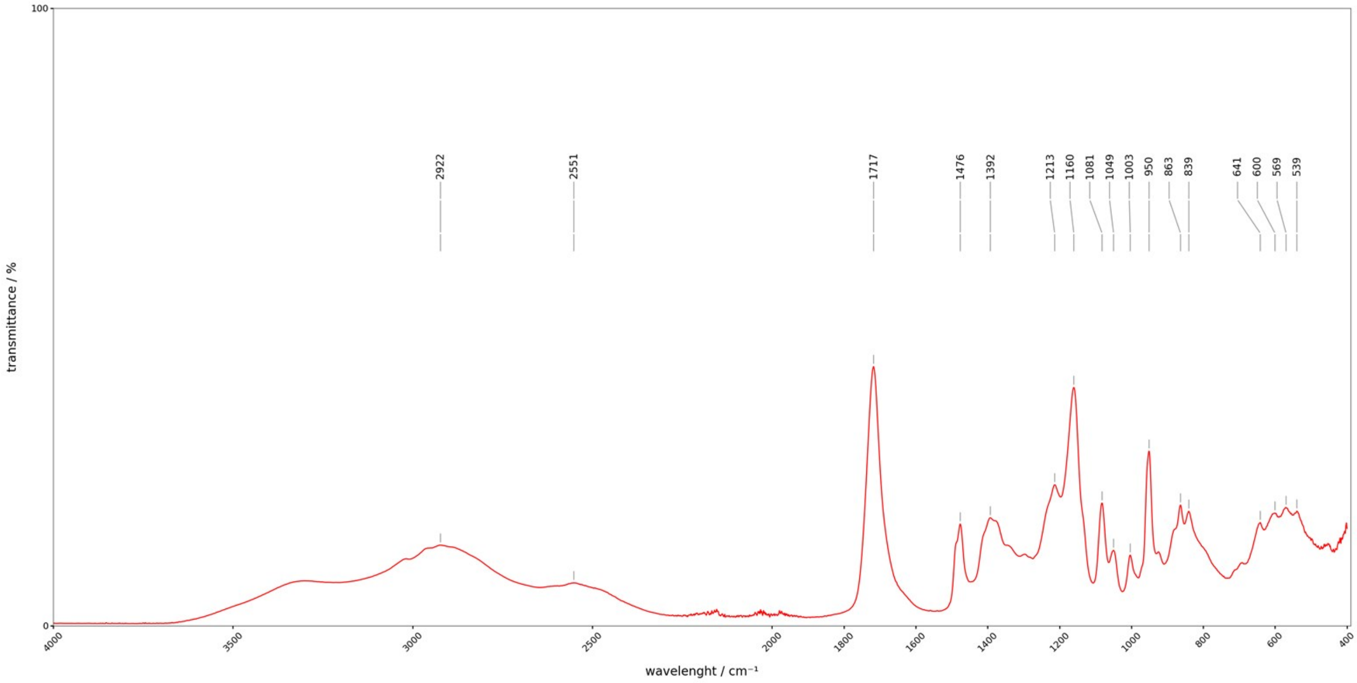

Figure S 55. IR spectra of ChCl:TMA(1:1).

Please do not adjust margins

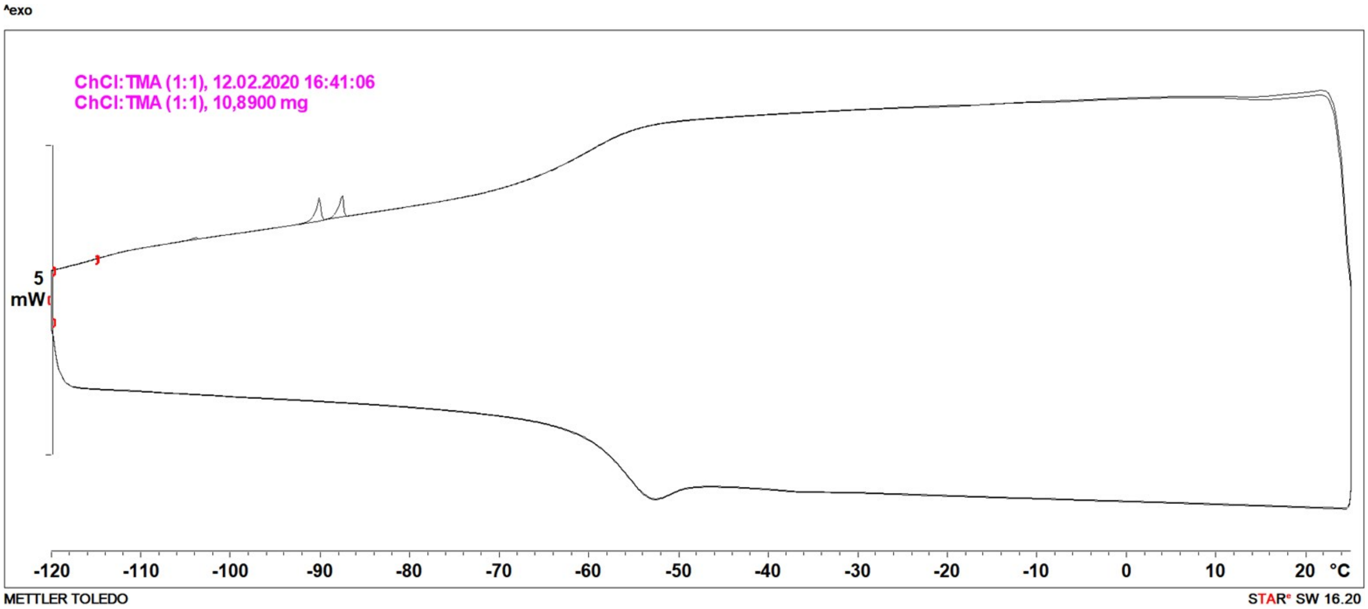

Figure S 56. DSC spectra of ChCl:TMA(1:1).

Please do not adjust margins

Please do not adjust margins

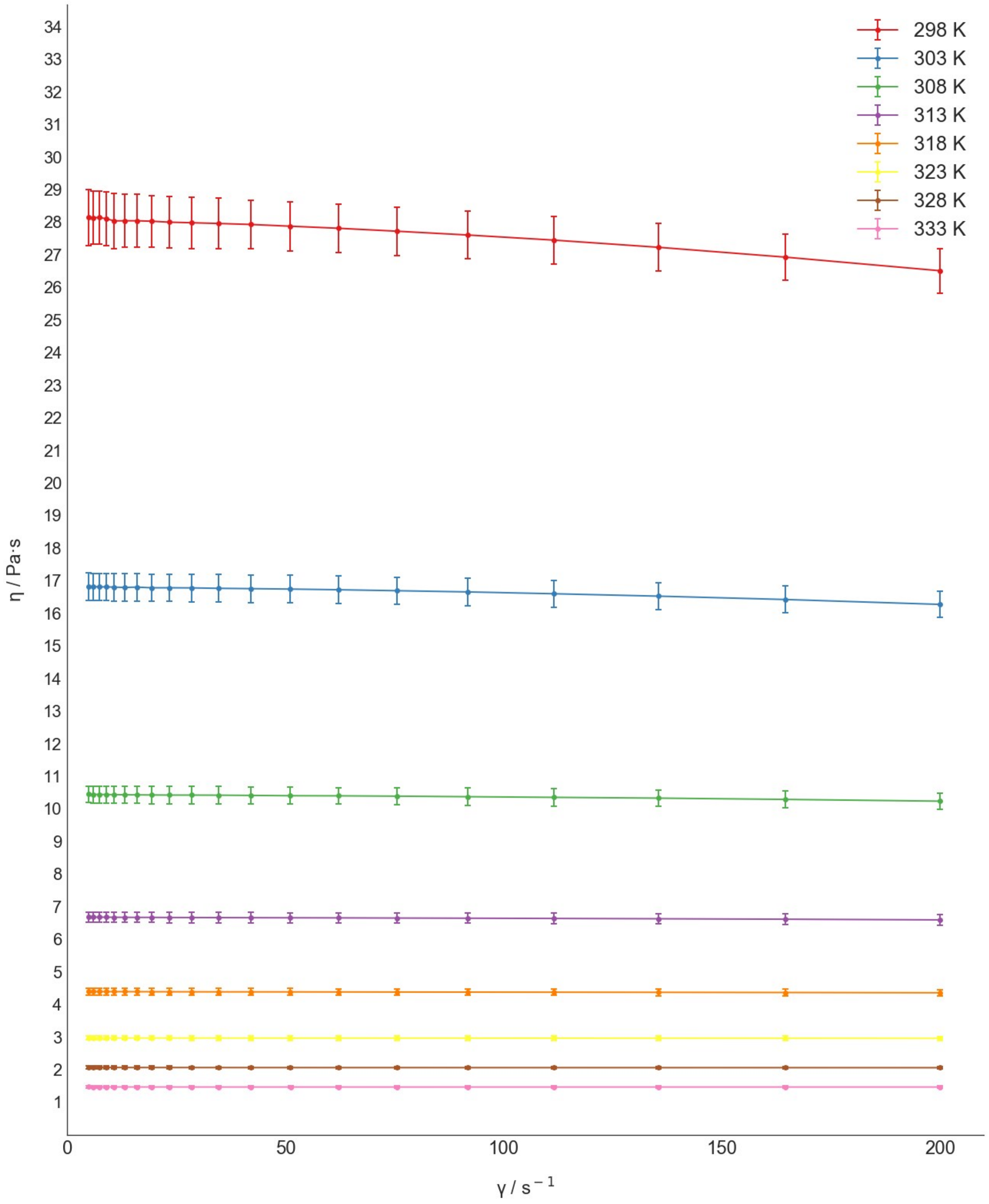

Figure S 57. Viscosity measurements for ChCl:TMA(1:1).

Please do not adjust margins

ChCl:DTT(1:2)

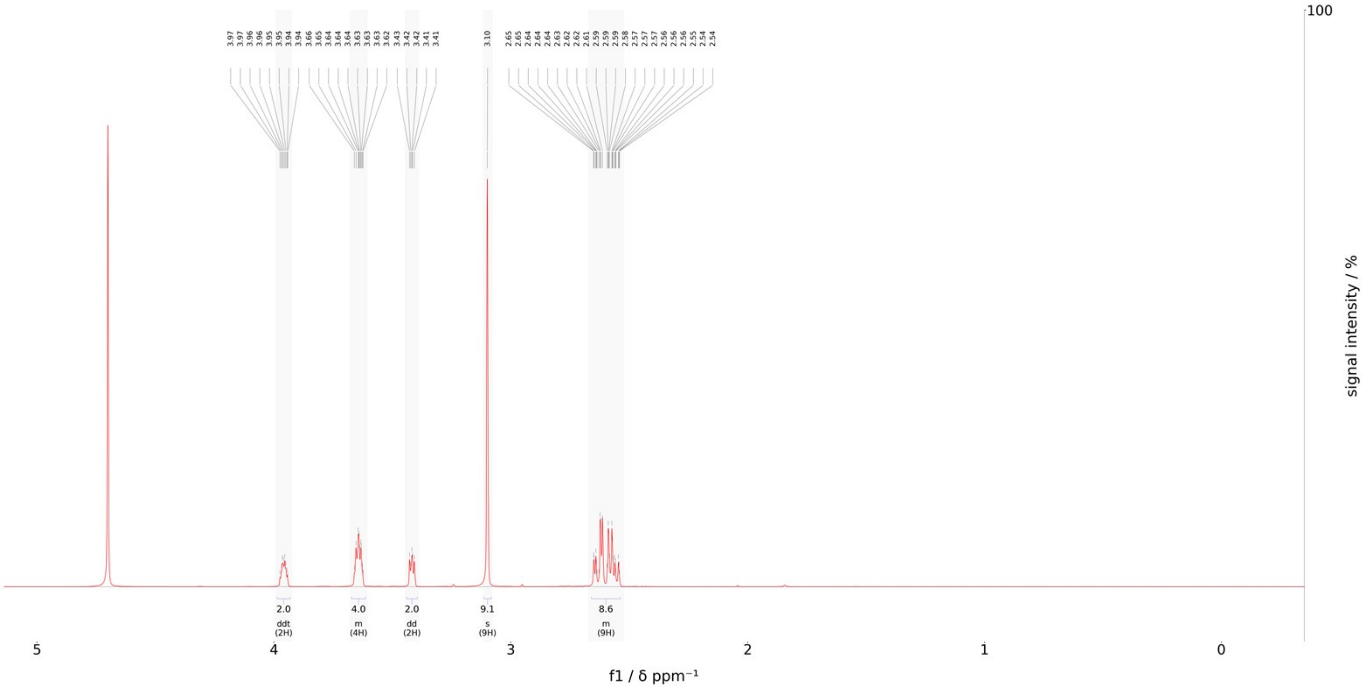

Figure S 58. ¹H NMR of ChCl:DTT(1:2) in deuterated water.

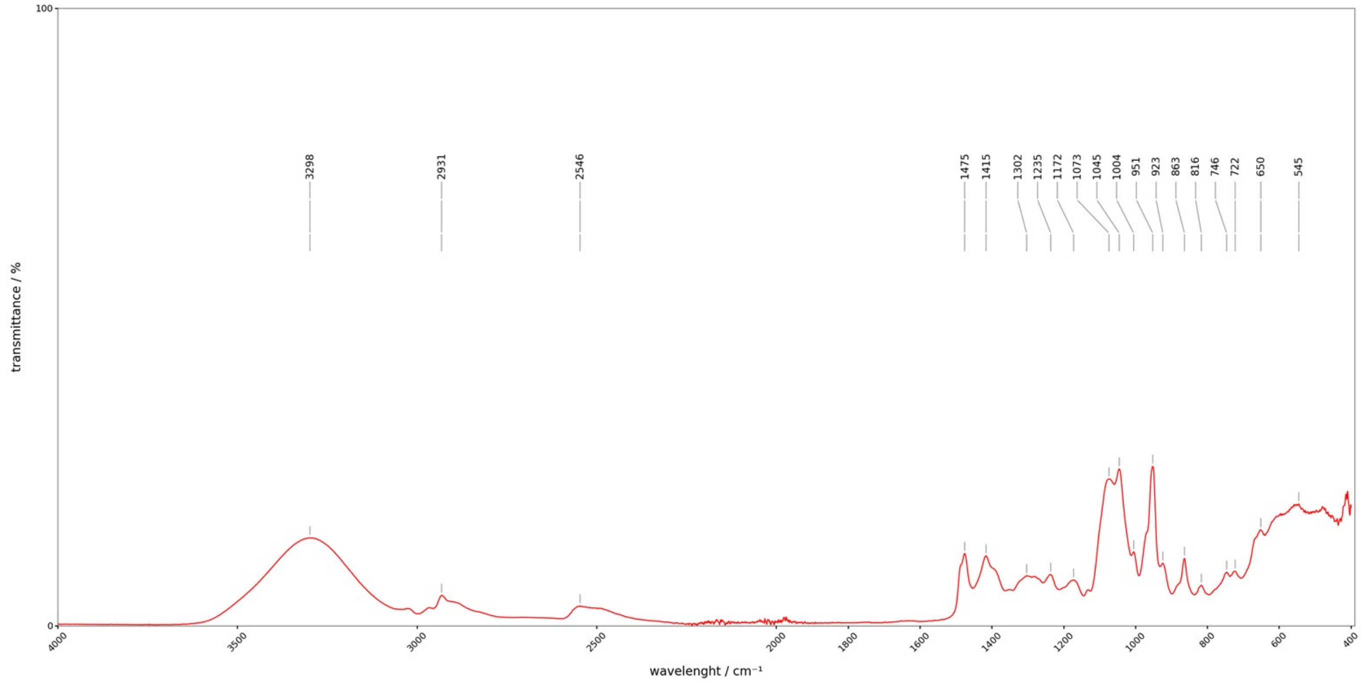

Figure S 59. IR spectra of ChCl:DTT(1:2).

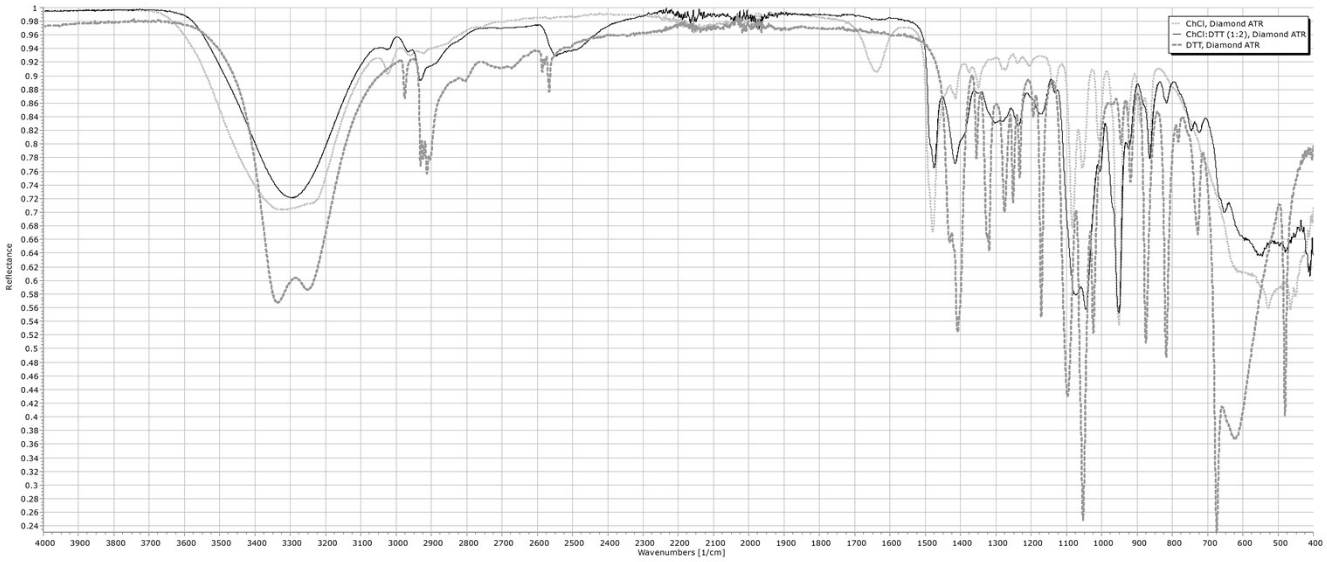

Figure S 60. IR spectra overlap of ChCl:DTT (black solid line), DTT (grey dashed line), and ChCl (light grey dotted line).

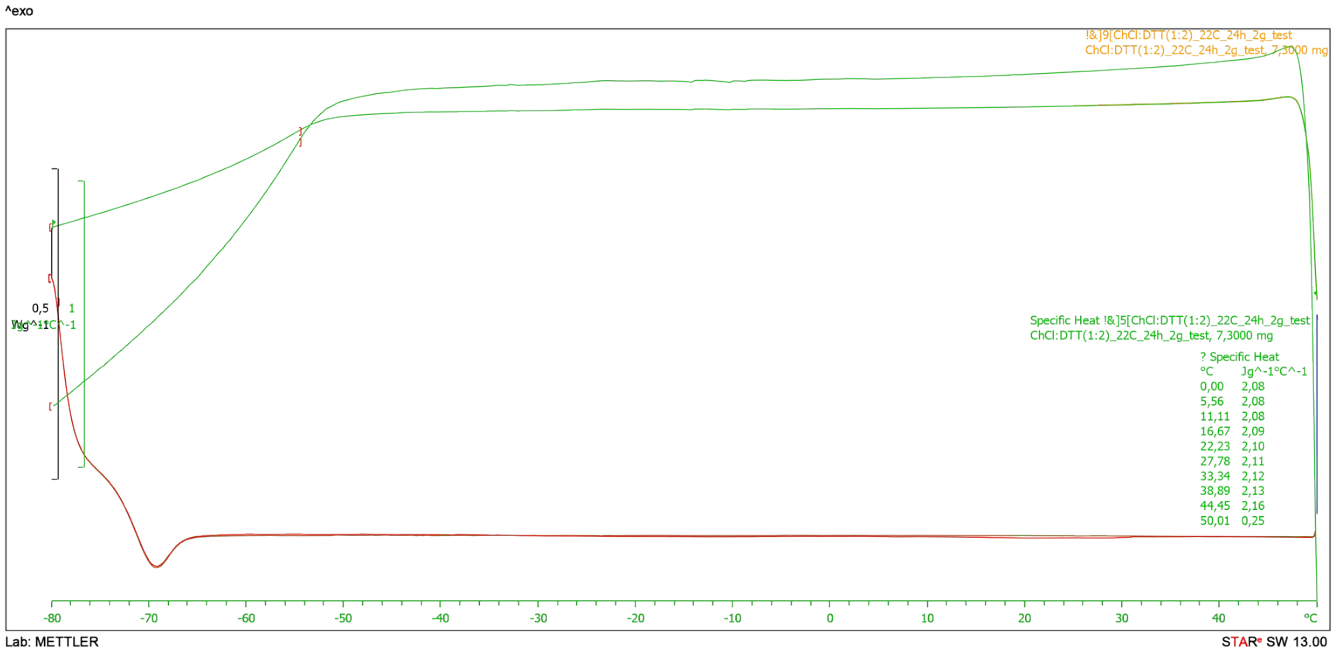

Figure S 61. DSC spectra of ChCl:DTT(1:2).

ChCl:DTT(1:3)

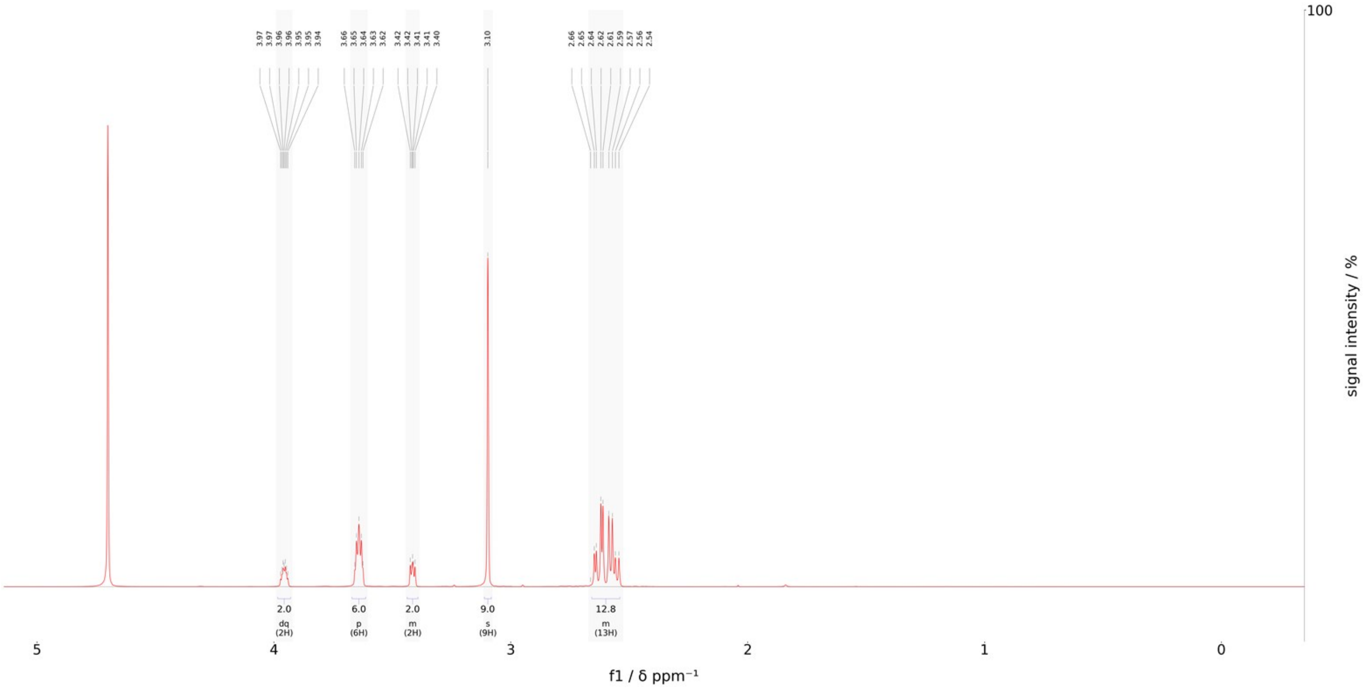

Figure S 62. ¹H NMR of ChCl:DTT(1:3) in deuterated water.

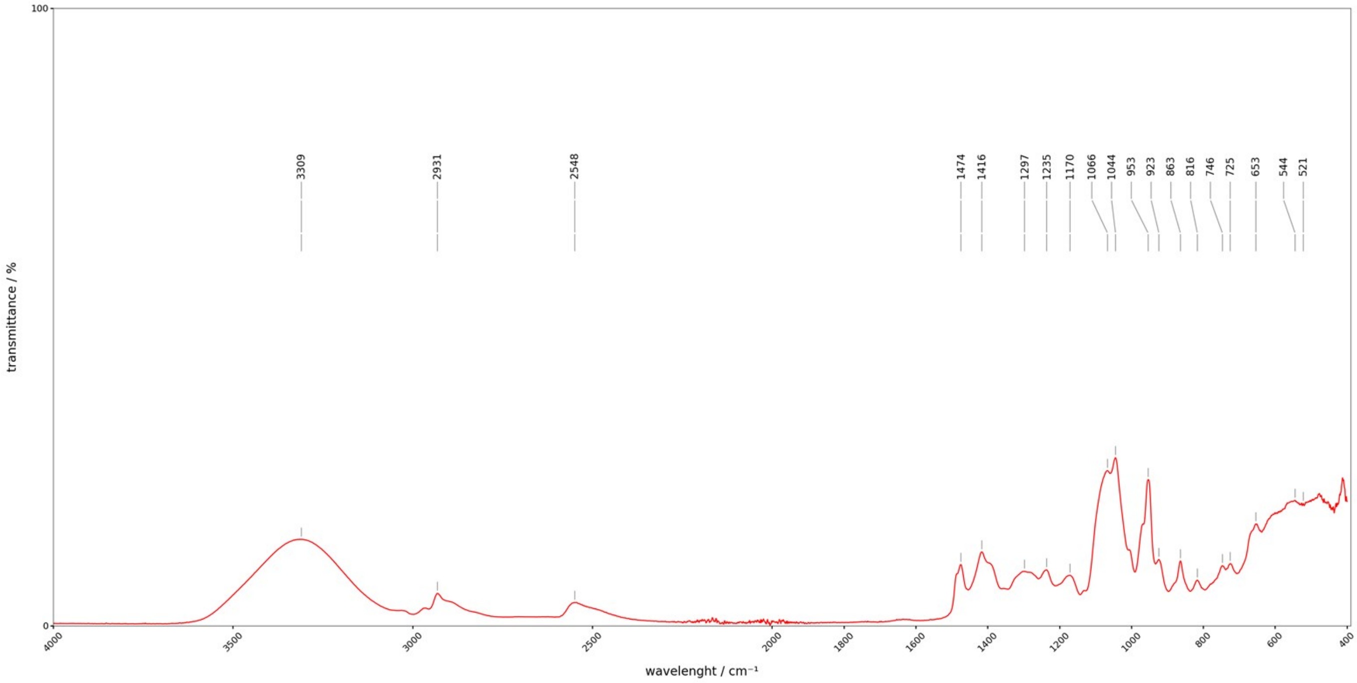

Figure S 63. IR spectra of ChCl:DTT(1:3).

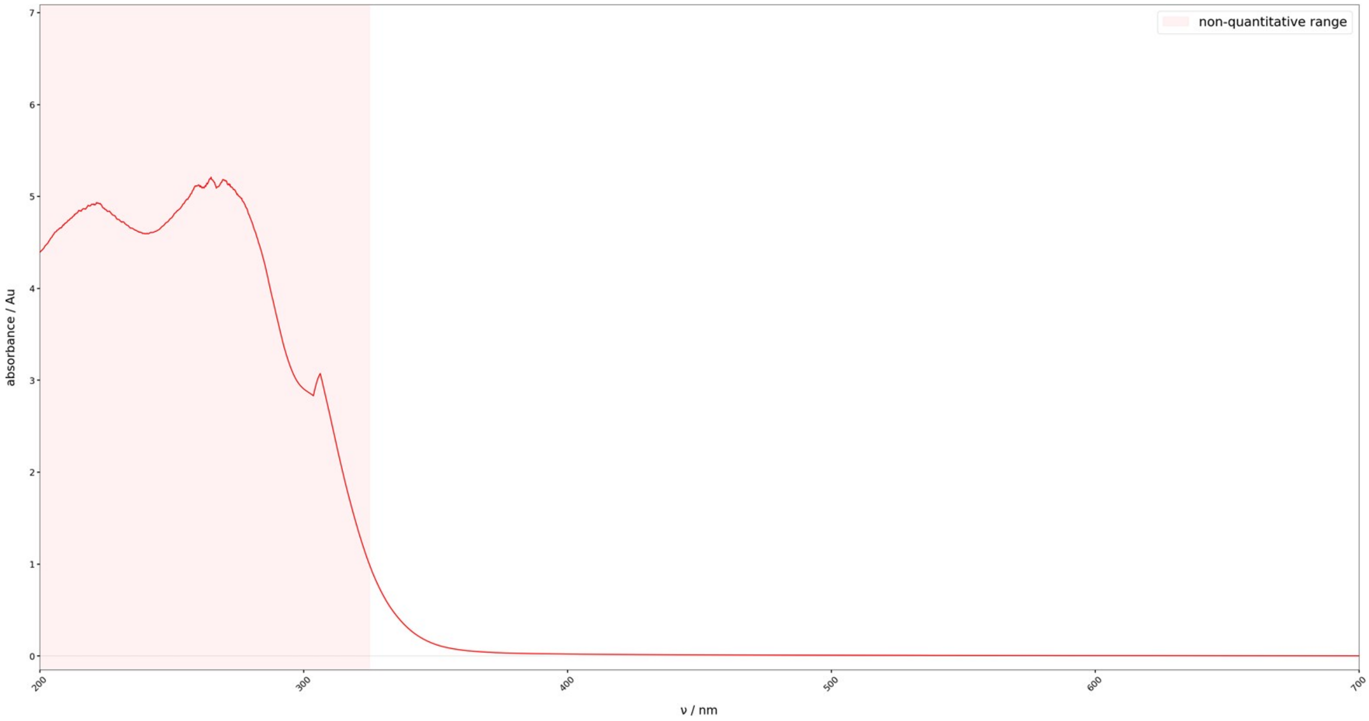

Figure S 64. UV spectra of ChCl:DTT(1:3).

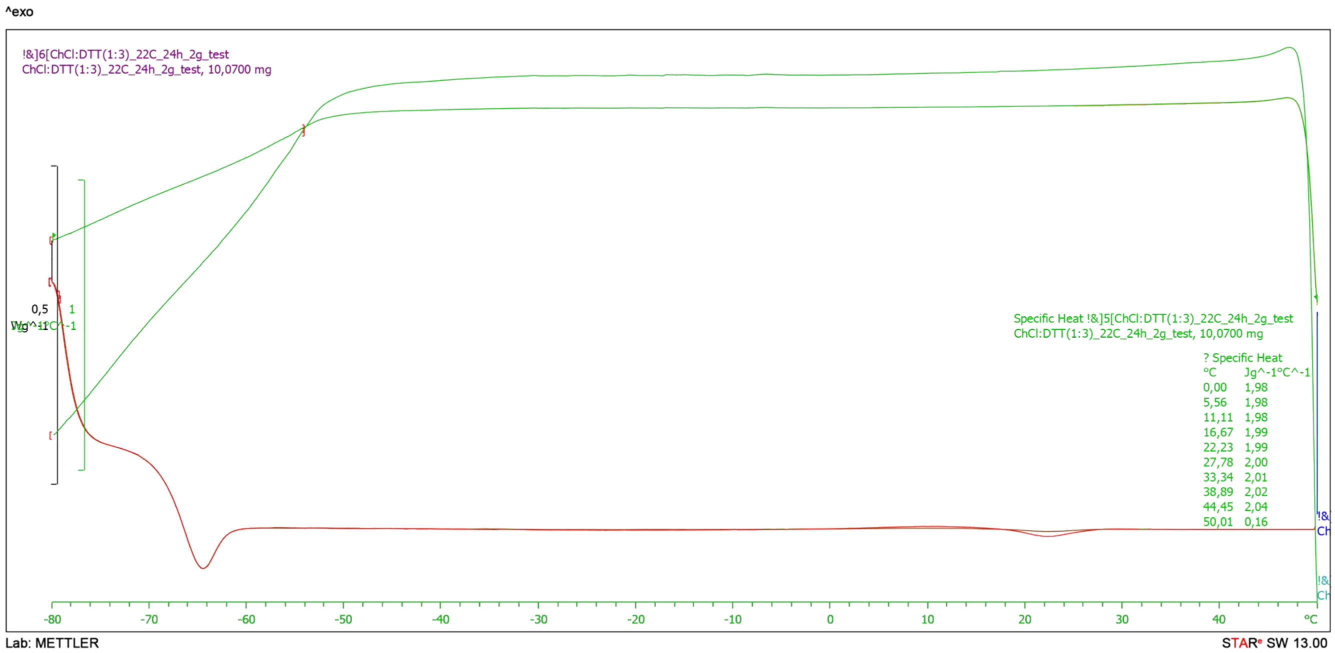

Figure S 65. DSC spectra of ChCl:DTT(1:3).

Please do not adjust margins

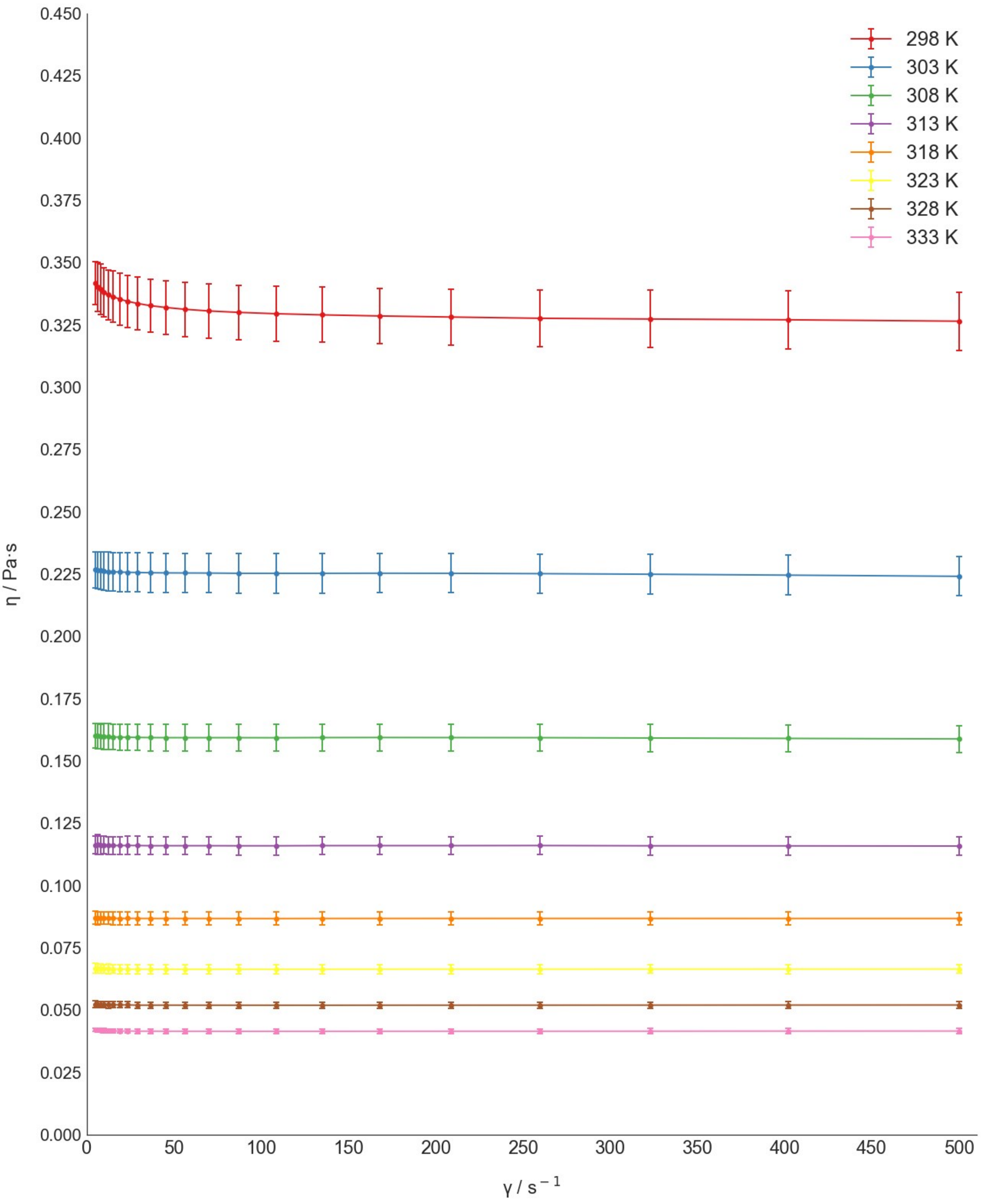

Figure S 66. Viscosity measurements for ChCl:DTT(1:3).

Please do not adjust margins
